# Arnold tongue entrainment reveals dynamical principles of the embryonic segmentation clock

**DOI:** 10.7554/eLife.79575

**Published:** 2022-10-12

**Authors:** Paul Gerald Layague Sanchez, Victoria Mochulska, Christian Mauffette Denis, Gregor Mönke, Takehito Tomita, Nobuko Tsuchida-Straeten, Yvonne Petersen, Katharina Sonnen, Paul François, Alexander Aulehla

**Affiliations:** 1 European Molecular Biology Laboratory (EMBL), Developmental Biology Unit Heidelberg Germany; 2 McGill University Montreal Canada; 3 European Molecular Biology Laboratory (EMBL), Transgenic Service Heidelberg Germany; National Centre for Biological Sciences­‐Tata Institute of Fundamental Research India; CNRS LPENS France

**Keywords:** entrainment, oscillations, somitogenesis, Mouse

## Abstract

Living systems exhibit an unmatched complexity, due to countless, entangled interactions across scales. Here, we aim to understand a complex system, that is, segmentation timing in mouse embryos, without a reference to these detailed interactions. To this end, we develop a coarse-grained approach, in which theory guides the experimental identification of the segmentation clock entrainment responses. We demonstrate period- and phase-locking of the segmentation clock across a wide range of entrainment parameters, including higher-order coupling. These quantifications allow to derive the phase response curve (PRC) and Arnold tongues of the segmentation clock, revealing its essential dynamical properties. Our results indicate that the somite segmentation clock has characteristics reminiscent of a highly non-linear oscillator close to an infinite period bifurcation and suggests the presence of long-term feedbacks. Combined, this coarse-grained theoretical-experimental approach reveals how we can derive simple, essential features of a highly complex dynamical system, providing precise experimental control over the pace and rhythm of the somite segmentation clock.

## Introduction

How do we gain insight into a complex system, which exhibits emergent properties that reflect the integration of entangled interactions and feedback regulation? As pointed out in the late 1970s by David Marr and Tomaso Poggio in their seminal paper (Marr and Poggio, 1976), understanding the complexity encountered when studying the ‘nervous system or a developing embryo’ requires the analysis at multiple levels of organization. Their core tenet is that also in biological systems, different levels of organization, while obviously causally linked, exhibit only a loose connection and importantly, can be studied and understood independently from each other.

Such observations are not specific to biology and have been made more quantitative in other fields. In physics, renormalization techniques coarse-grain degrees of freedom to obtain scale-free theories, allowing to define universality classes independent of the precise details of interactions (Cardy, 1996; Bialek, 2015). Another recent example is the parameter space compression theory, showing how complex systems (in biology or physics) can be typically reduced to simpler descriptions with few parameters (Machta et al., 2013; Proulx-Giraldeau et al., 2017; Hsu et al., 2020).

Going one step further, this suggests that one might be able to study – and control – complex systems provided we identify the essential, macro-level behavior. This is possible because only a limited number of universal descriptions exist, with defining behaviors and properties, that do not depend on the detailed implementation. A central challenge that remains is to implement these theoretical ideas to the experimental study of biological complexity.

Here, we develop a coarse-grained approach combining theory and experiments to study a cellular oscillator ensemble that constitutes the embryonic somite segmentation clock. Functionally, this clock controls the periodic formation of somites, the precursors of the vertebral column, and other tissues (Palmeirim et al., 1997; Krol et al., 2011). Molecularly, the segmentation clock comprises the oscillatory activity of several major signaling pathways, such as the Notch, Wnt, and Fgf signaling pathways, which show oscillatory dynamics with a period matching somite formation, that is, ∼2 hr in mouse embryos (Dequéant and Pourquié, 2008; Hubaud and Pourquié, 2014; Aulehla et al., 2003; Dequéant et al., 2006; Aulehla et al., 2008). More recently, segmentation clock oscillations with a period of ∼5 hr have been identified in human induced pluripotent stem cells differentiated into paraxial mesoderm, identifying a set of ∼200 oscillating genes, including targets of Notch and Wnt signaling (Chu et al., 2019; Diaz-Cuadros et al., 2020; Matsuda et al., 2020).

Strikingly, as individual oscillating cells are coupled to their neighbors via Notch-Delta signaling, the oscillations occur synchronized and wave-like activity patterns appear to periodically sweep along the embryonic anterior-posterior axis (Maroto et al., 2005; Masamizu et al., 2006; Aulehla et al., 2008; Herrgen et al., 2010; Webb et al., 2016; Yoshioka-Kobayashi et al., 2020; Rohde et al., 2021).

Adding to the complexity, these periodic spatiotemporal wave patterns are linked to an underlying spatial period gradient along the embryonic axis, that is, signaling dynamics in cells close to the posterior of the embryo oscillate faster compared to those in cells located more anteriorly. Such a period gradient linked to the segmentation clock has been identified in several species (Gomez et al., 2008; Morelli et al., 2009; Oates et al., 2012; Lauschke et al., 2013; Soroldoni et al., 2014; Tsiairis and Aulehla, 2016; Falk et al., 2022) and also in in vitro assays culturing intact or even dissociated presomitic mesoderm (PSM) (Lauschke et al., 2013; Tsiairis and Aulehla, 2016).

Of note, an analogous oscillatory system was also described during segmentation in arthropods (Clark et al., 2019) and while distinct at molecular level, it also exhibits spatiotemporal wave patterns traversing the embryo axis, again with indication of a period gradient (Sarrazin et al., 2012; El-Sherif et al., 2012; Brena and Akam, 2013; Schönauer et al., 2016).

**Figure 1.**
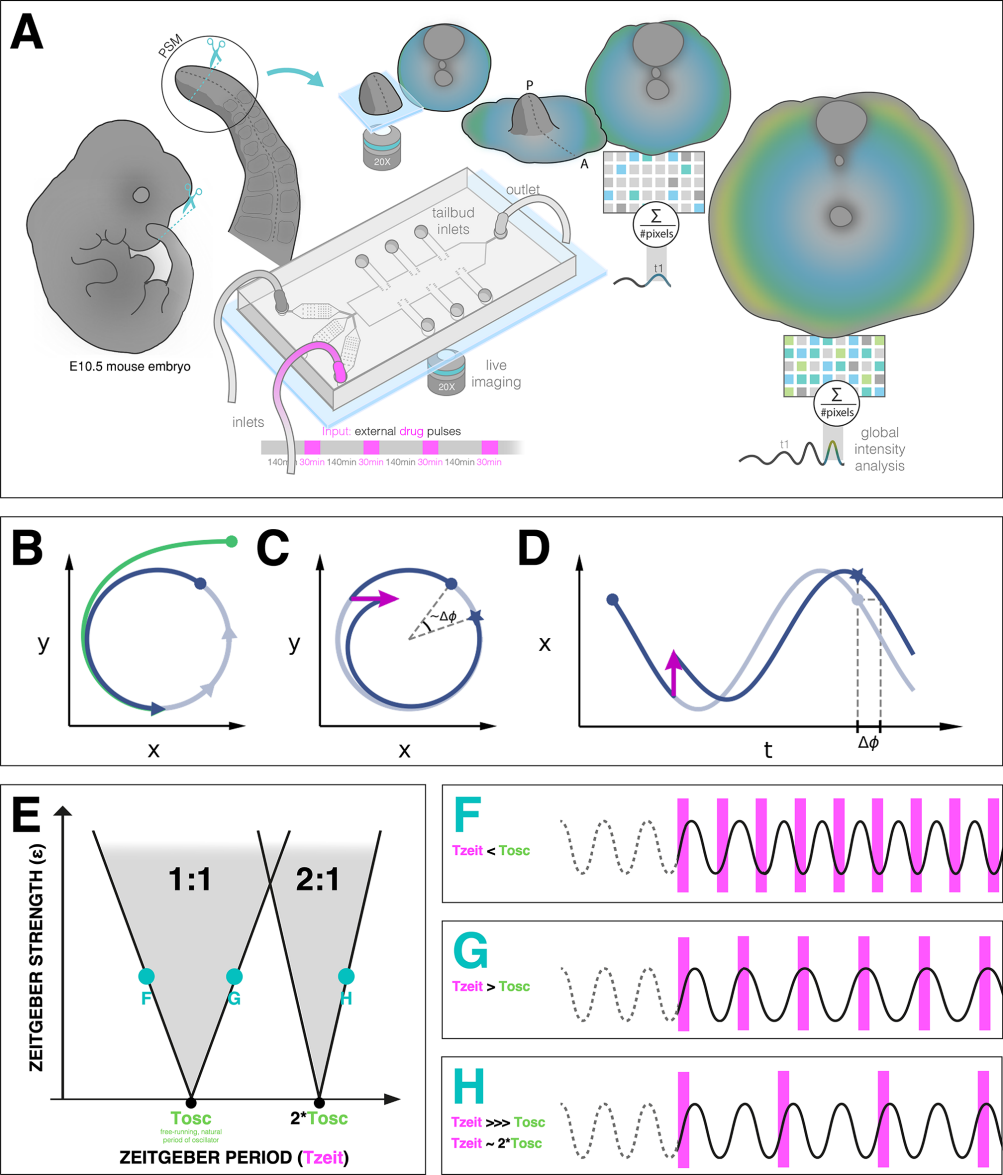
Entrainment of an embryonic oscillator with a *zeitgeber*: setup and theory. (**A**) Schematic of the experimental entrainment setup using a microfluidics device previously described in Sonnen et al., 2018, and an overview of the analysis approach used in this present study. (1) The posterior presomitic mesoderm (PSM) is recovered from E10.5 mouse embryo and used in a quasi-two-dimensional segmentation assay (‘2D-assay’) (Lauschke et al., 2013). (2) The embryonic tissue is loaded into a microfluidics device bonded to fibronectin-coated cover glass, and is imaged using a ×20 objective at 37°C and 5% CO_2_. (3) Simultaneously, the 2D-assay is subjected to periodic pulses of a drug (e.g. DAPT for Notch signaling), which serves as *zeitgeber*. (4) Using dynamic fluorescent reporter for segmentation clock genes (e.g. LuVeLu for Notch signaling), we quantify endogenous signaling oscillations during the experiment. (5) We generate a single timeseries using reporter signal from the entire sample (‘global region of interest [ROI]’) that allows to quantify the segmentation clock rate and rhythm (see validation in Figure 2 and Figure 7—figure supplement 3). Illustration by Stefano Vianello. A photo of the actual microfluidics device and its design are shown in Figure 1—figure supplement 1. (**B**) Abstract definition of phase: two different points in the plane (x,y) have the same phase if they converge on the same point on the limit cycle (indicated in gray). (**C**) Perturbations change the phase of the cycle by an increment Δ⁢ϕ (here phase is defined by the angle in the plane). (**D**) Time courses with similar perturbation as C showing the oscillations of x and phase difference Δ⁢ϕ. (**E**) Illustration of generic Arnold tongues, plotted as a function of *zeitgeber* strength (ϵ) and *zeitgeber* period ($T_{z\,e\,i\,t}$), mapping n:m entrainment where the entrained oscillator (with natural period of $T_{o\,s\,c}$) goes through n cycle/s for every m cycle/s of the *zeitgeber*. Three different points in the 1:1 and 2:1 Arnold tongues are specified with corresponding graphical illustration of an autonomous oscillator as it is subjected to *zeitgeber* with different periods ($T_{z\,e\,i\,t}$): when $T_{z\,e\,i\,t}$ is less than $T_{o\,s\,c}$ (**F**), when $T_{z\,e\,i\,t}$ is greater than $T_{o\,s\,c}$ (**G**), and when $T_{z\,e\,i\,t}$ is much much greater than $T_{o\,s\,c}$ but is close to twice of $T_{o\,s\,c}$ (**H**). Free-running rhythm of the oscillator (i.e. before perturbation) is marked by a dashed line, while solid line illustrates its rhythm during perturbation with *zeitgeber*. Magenta bars represent the *zeitgeber* pulses. Illustration by Stefano Vianello.

In this work, we coarse-grain these underlying complexities and take a dynamical systems, macro-perspective on the segmentation clock, studying it as a single phase oscillator (Figure 1A). We build on the theory of synchronization and entrainment (see below) to first perform a systematic experimental characterization of its response to perturbation. We compare the outcome to qualitative and quantitative theoretical predictions. In turn, these experimental quantifications allow to derive a phase response curve (PRC) that uniquely characterizes the dynamical properties of the segmentation clock. This new insight provides the means to understand – and control – the timing of a complex embryological patterning process.

**Figure 1—figure supplement 1.**
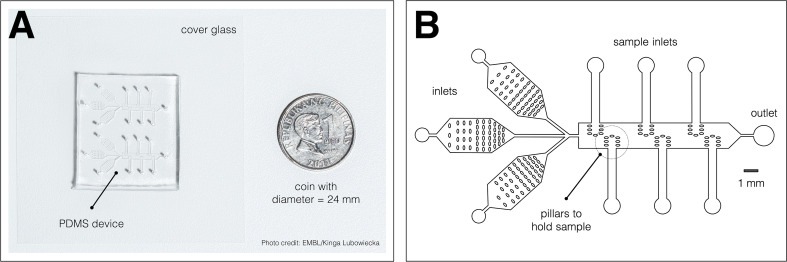
Microfluidics device. A microfluidics device for simultaneous culture, imaging, and entrainment of the segmentation clock in 2D-assays. (**A**) Photo of the chip, previously described in Sonnen et al., 2018, bonded to cover glass and a coin (diameter: 24 mm) for scale. The split layout separating the upper and lower channel systems allows simultaneous delivery of drug and DMSO control to samples on opposite sides of the same device. Photo credit: EMBL/Kinga Lubowiecka. (**B**) Design of the microfluidics chip, showing inlets for medium and drug, inlets for the samples, pillars to hold each sample, and an outlet.

### Theory of synchronization guides the experimental study of segmentation clock entrainment

Our experimental study is based on and guided by the theory of entrainment of oscillators by an external periodic signal – a subset of the general theory of synchronization (Pikovsky et al., 2001).

Entrainment is observed when an autonomous oscillator adapts its behavior to lock to an external periodic signal (called *zeitgeber* in the circadian rhythm literature). The general theoretical framework to understand entrainment requires the definition of oscillator phases (Figure 1B–D), and their response to perturbation (Kuramoto, 2003). Assuming the *zeitgeber* consists in periodic pulses, entrainment is observed when the phase of the oscillator $\phi_{e\,n\,t}$ at the time of the *zeitgeber* is constant (technically, a fixed point of the Poincaré return map [Cross and Siggia, 2005]). This defines period-locking (also termed mode-locking) (Pikovsky et al., 2001). Entrainment is not always manifested and conditions for its existence can be derived. Quantitatively, when entrainment occurs, the *zeitgeber* induces a periodic phase perturbation (or response) of the entrained oscillator, which *exactly* compensates the detuning (or period mismatch, $T_{z\,e\,i\,t}-T_{o\,s\,c}$) between the *zeitgeber* and the free-running oscillator. For this reason, when the detuning is very small, a weak external perturbation is enough to entrain an oscillator. Conversely, if the detuning is big, a strong signal and associated response is required for entrainment. One can then plot the minimal strength of the *zeitgeber* (ϵ) versus the corresponding detuning (or simply $T_{z\,e\,i\,t}$, if $T_{o\,s\,c}$ is constant): these maps are more commonly known as Arnold tongues (Figure 1E). Arnold tongues predict the period- and phase-locking behavior in oscillatory systems as different as electrical circuits (Balanov, 2008), oscillatory chemical reactions (Ito, 1979; Kai and Tomita, 1979; Buchholz et al., 1985; Makki et al., 2014), or living systems such as circadian rhythms (Granada et al., 2013).

Lastly, more complex patterns of entrainment can be observed: for instance, stable phase relationships can be established where the entrained oscillator goes through n cycles for every m cycles of the external signal, defining n:m period-locking. In that case, the instantaneous period of the oscillator matches m/n the period of the *zeitgeber* ($T_{z\,e\,i\,t}$). Corresponding Arnold tongues can be obtained, leading to a rich structure for entrainment in parameter space (Figure 1F–H).

Previously, periodic activation of Notch signaling via heat shock-driven expression of the ligand Delta was used to modulate the segmentation of PSM in zebrafish (Soza-Ried et al., 2014). In the cited study, the readout to assess the effect of the periodic perturbation relied on somite size and morphology. While this gave important insight into how morphological segmentation of the PSM and somite length are affected by a *zeitgeber*, experimental investigation on the underlying signaling dynamics upon periodic perturbation remains limited.

In this work, to experimentally apply the theory of synchronization to the segmentation clock, we make use of a microfluidics-based entrainment setup, which we had established previously in the lab (Figure 1—figure supplement 1; Sonnen et al., 2018).

We showed before that using a quasi-two-dimensional in vitro segmentation assay (hereafter referred to as a 2D-assay), which recapitulates segmentation clock dynamics and PSM patterning (Lauschke et al., 2013), the microfluidics-entrainment approach allowed us to take control of Notch and Wnt signaling oscillations, providing direct functional evidence that the oscillation phase shift between Wnt and Notch signaling is critical for PSM patterning (Sonnen et al., 2018).

## Results

### A coarse-grained, single oscillator description of the segmentation clock

**Figure 2.**
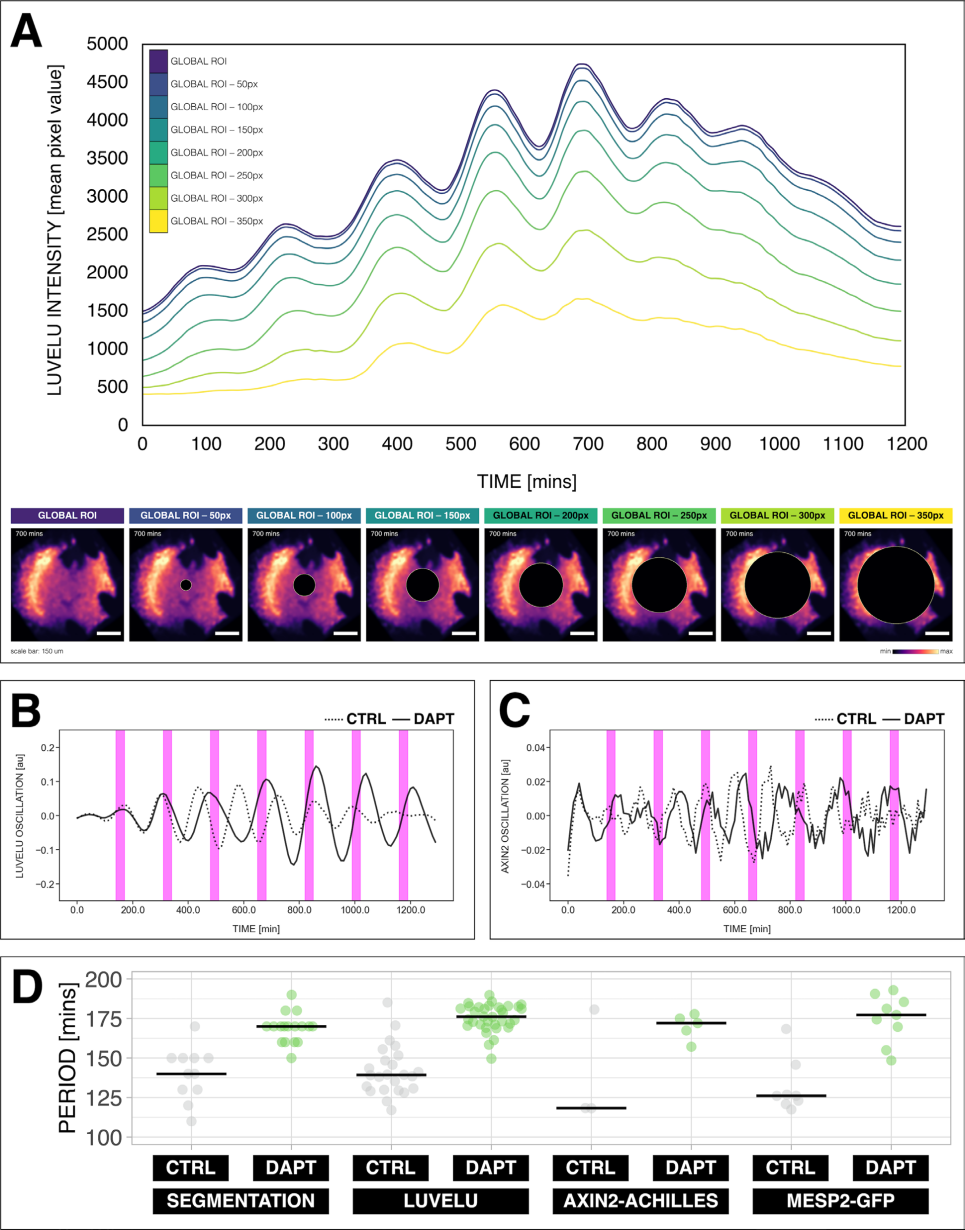
Quantifying the segmentation clock rhythm. (**A**) Comparison of measurements obtained from a region of interest (ROI) spanning the entire field (‘global ROI’) with those obtained from ROIs in which central regions of increasing size were excluded. The excluded area is represented in terms of the diameter (in pixels, px) of a circular region at the center. The corresponding timeseries are shown in the top panel and marked with different colors. Bottom panel shows the global ROI and excluded region in a snapshot of signal of LuVeLu, a dynamic reporter of Notch signaling driven from the *Lfng* promoter (Aulehla et al., 2008), in a 2D-assay at 700 min from the start of the experiment. Henceforth, timeseries is obtained using ‘global ROI’, unless otherwise specified. (**B**) Detrended timeseries of the segmentation clock (obtained using global ROI) in 2D-assays, which express the LuVeLu reporter, subjected to 170 min periodic pulses (magenta bars) of either 2 µM DAPT (in solid line) or DMSO (for control, in dashed line). Timelapse movie and corresponding timeseries from global ROI are available at https://youtu.be/fRHsHYU_H2Q. (**C**) Detrended timeseries of Axin2-linker-Achilles reporter in 2D-assays, subjected to 170 min periodic pulses (magenta bars) of either 2 µM DAPT (in solid line) or DMSO (for control, in dashed line). (**D**) Period of morphological segment boundary formation, LuVeLu oscillation, Axin2-linker-Achilles oscillation, and rhythm of Mesp2-GFP in 2D-assays subjected to 170 min periodic pulses of either 2 µM DAPT (or DMSO for control). Each sample is represented as a dot, the median is denoted as a solid horizontal line. For morphological segment boundary formation, period was determined by taking the time difference between two consecutive segmentation events (for CTRL: 17 segmentation events in six samples from four independent experiments, and for DAPT: 24 segmentation events in seven samples from five independent experiments, with p-value < 0.001) in the brightfield channel. For period quantifications based the reporters, the mean period per sample from 650 to 850 min after start of the experiment was plotted. LuVeLu: (CTRL: *n*=24 and *N*=7) and (DAPT: *n*=34 and *N*=8) with p-value < 0.001, Axin2-linker-Achilles: (CTRL: *n*=3 and *N*=1) and (DAPT: *n*=5 and *N*=1) with p-value = 0.107, Mesp2-GFP: (CTRL: *n*=8 and *N*=3) and (DAPT: *n*=9 and *N*=3) with p-value = 0.01. Data were visualized using PlotsOfData (Postma and Goedhart, 2019). To calculate the p-value, two-tailed test for absolute difference between medians was done via a randomization method using PlotsOfDifferences (Goedhart, 2019). The timeseries and corresponding period evolution during entrainment, obtained from wavelet analysis, are in Figure 2—figure supplement 1A and Figure 2—figure supplement 1B, respectively. Timelapse movies are available at https://youtu.be/edFczx_-9hM and https://youtu.be/tQeBk0_U_Qo, respectively.

To perform a systematic analysis of entrainment dynamics, we first introduced a single oscillator description of the segmentation clock. We used the segmentation clock reporter LuVeLu, which shows highest signal levels in regions where segments form (Aulehla et al., 2008). Hence, we reasoned that a global region of interest (ROI) quantification, averaging LuVeLu intensities over the entire sample, should faithfully report on the segmentation rate and rhythm, essentially quantifying ‘wave arrival’ and segment formation in the periphery of the sample. Indeed, our validation confirmed that the oscillation period based on the global ROI analysis using the LuVeLu reporter closely matched the rate of morphological segment boundary formation (Figure 2D, Figure 2—figure supplement 1). In addition, global ROI quantifications using additional reporters to measure Wnt signaling oscillations (i.e. Axin2, Figure 2C–D, Figure 2—figure supplement 1) and the segmentation marker Mesp2 (Figure 2D, Figure 2—figure supplement 1) also showed close correspondence with LuVeLu global ROI measurements of segmentation clock period.

**Figure 2—figure supplement 1.**
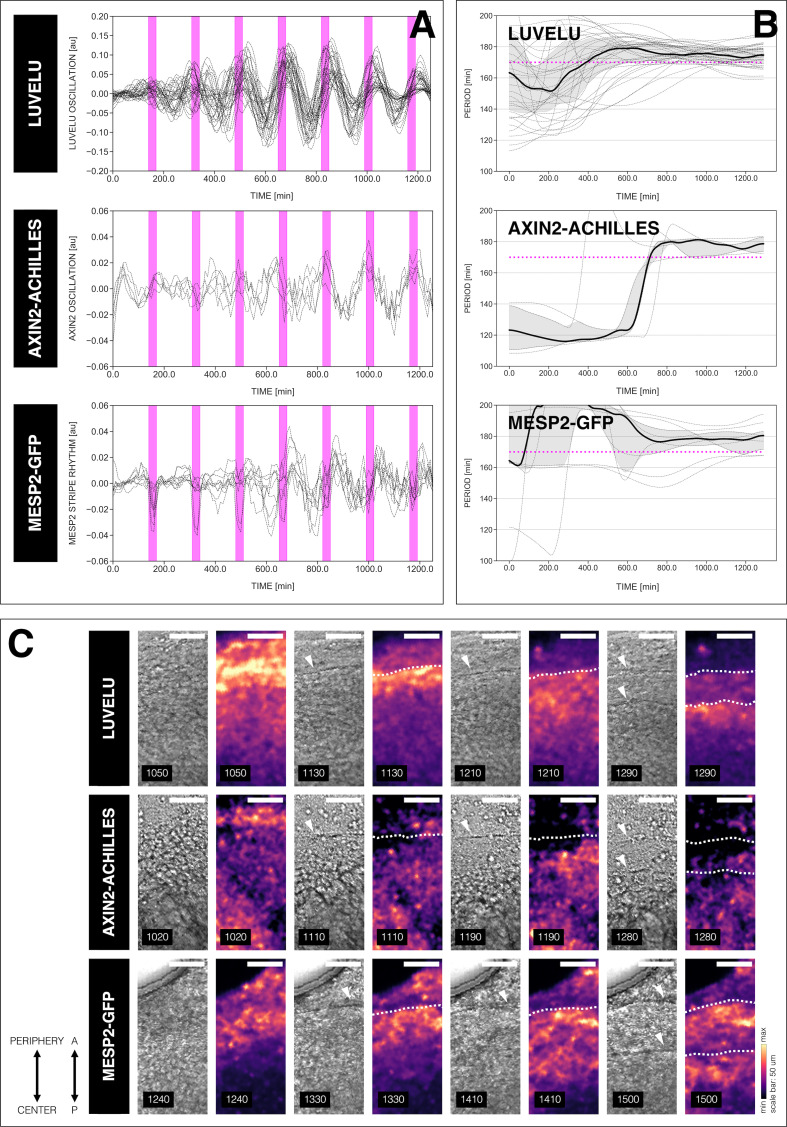
Different readouts of the segmentation clock. The rhythms of different dynamic readouts of the entrained segmentation clock match. (**A**) Detrended timeseries of the segmentation clock in 2D-assays subjected to 170 min periodic pulses of 2 µM DAPT, and expressing either LuVeLu (Aulehla et al., 2008), Axin2-linker-Achilles, or Mesp2-GFP (Morimoto et al., 2006). Periodic pulses are indicated as magenta bars and the timeseries of each sample (for LuVeLu: *n*=34 and *N*=8, for Axin2-linker-Achilles: *n*=5 and *N*=1, for Mesp2-GFP: *n*=9 and *N*=3) is marked with a dashed line. The detrended timeseries for samples expressing LuVeLu is the same as the detrended timeseries for the DAPT condition in Figure 3A. (**B**) Period evolution during entrainment, obtained from wavelet analysis. The period evolution for each sample and the median of the periods are represented here as a dashed line and a solid line, respectively. The gray shaded area corresponds to the interquartile range. Magenta dashed line marks $T_{z\,e\,i\,t}$. The period evolution plot for samples expressing LuVeLu is the same as the period evolution plot for the DAPT condition in Figure 3B. (**C**) Snapshots of segmenting regions in 2D-assays subjected to 170 min periodic pulses of 2 µM DAPT, and expressing either LuVeLu (Aulehla et al., 2008), Axin2-linker-Achilles, or Mesp2-GFP (Morimoto et al., 2006), at different time points. Segment boundaries are marked with either white arrowheads (brightfield channel) or white dashed lines (reporter channel). Samples are re-oriented so that the top is toward the periphery and the bottom is toward the center of the 2D-assay. Time is indicated as minutes elapsed from the start of the experiment. Scale bar: 50 µm. Timelapse movies of 2D-assays expressing either Axin2-linker-Achilles or Mesp2-GFP subjected to said perturbation are available at https://youtu.be/edFczx_-9hM and https://youtu.be/tQeBk0_U_Qo, respectively.

**Figure 2—video 1.** E10.5 2D-assay, expressing LuVeLu, subjected to 170 min periodic pulses of 2 µM DAPT (or DMSO for control), with corresponding timeseries obtained using a global region of interest (ROI) spanning the entire field of view, available at https://youtu.be/fRHsHYU_H2Q.

**Figure 2—video 2.** E10.5 2D-assay, expressing Axin2-linker-Achilles, subjected to 170 min periodic pulses of 2 µM DAPT (or DMSO for control) available at https://youtu.be/edFczx_-9hM.

**Figure 2—video 3.** E10.5 2D-assay, expressing Mesp2-GFP, subjected to 170 min periodic pulses of 2 µM DAPT (or DMSO for control) available at https://youtu.be/tQeBk0_U_Qo.

Hence, we conclude that LuVeLu global ROI timeseries analysis provides a valid coarse-grained quantification of the pace (period ∼ rate of segment formation) and rhythm (phase) of the segmentation clock.

### The pace of the segmentation clock can be locked to a wide range of entrainment periods

Having established a quantitative, coarse-grained readout for segmentation clock pace and rhythm, we next analyzed whether pace and rhythm can be experimentally tuned using microfluidics-based entrainment (Figure 1A, Figure 1—figure supplement 1).

**Figure 3.**
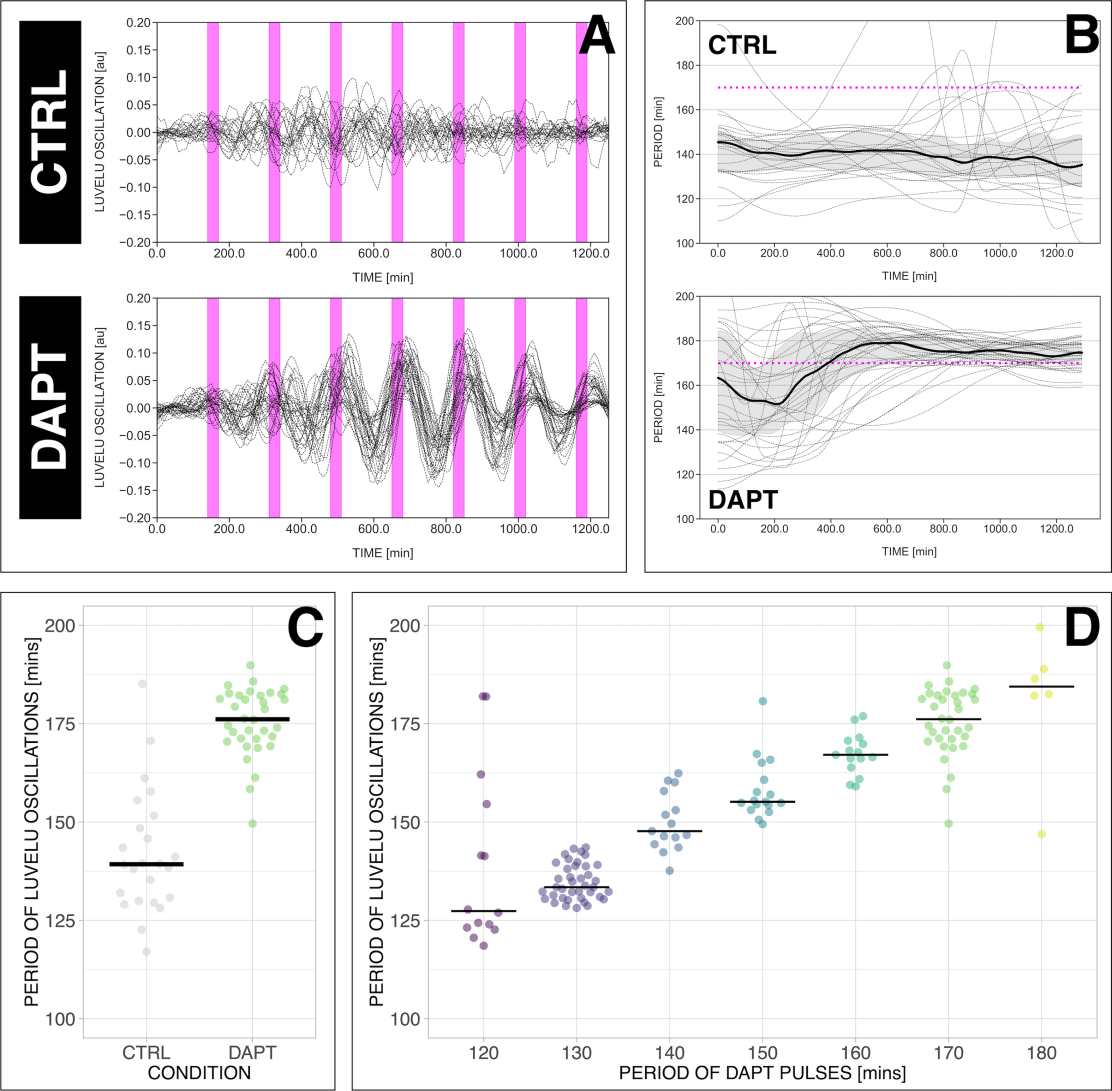
The segmentation clock can be locked to a wide range of entrainment periods. (**A**) Detrended (via sinc filter detrending) timeseries of the segmentation clock in 2D-assays subjected to 170 min periodic pulses of 2 µM DAPT (or DMSO for controls). Periodic pulses are indicated as magenta bars and the timeseries of each sample (for CTRL: *n*=24 and *N*=7, for DAPT: *n*=34 and *N*=8) is marked with a dashed line. (**B**) Period evolution during entrainment, obtained from wavelet analysis. The period evolution for each sample and the median of the periods are represented here as a dashed line and a solid line, respectively. The gray shaded area corresponds to the interquartile range. Magenta dashed line marks $T_{z\,e\,i\,t}$. (**C**) Mean period from 650 to 850 min after start of the experiment of samples subjected to 170 min periodic pulses of 2 µM DAPT (or DMSO for controls), with p-value <0.001. Each sample is represented as a dot, while the median of all samples is denoted as a solid horizontal line. This plot is the same as the plot for the LuVeLu condition in Figure 2D. (**D**) Mean period from 650 to 850 min after start of the experiment of samples entrained to periodic pulses of 2 µM DAPT. Each sample is represented as a dot, while the median of all samples is denoted as a solid horizontal line. The period of the DAPT pulses is specified (for 120 min: *n*=14 and *N*=3, for 130 min: *n*=39 and *N*=10, for 140 min: *n*=15 and *N*=3, for 150 min: *n*=17 and *N*=4, for 160 min: *n*=15 and *N*=3, for 170 min: *n*=34 and *N*=8, for 180 min: *n*=6 and *N*=1). Data were visualized using PlotsOfData (Postma and Goedhart, 2019), and a summary is provided in Table 1. A similar plot including each condition’s respective control is in Figure 3—figure supplement 1. The analysis of period and wavelet power across time is summarized in Figure 3—figure supplement 2. To calculate the p-value, two-tailed test for absolute difference between medians was done via a randomization method using PlotsOfDifferences (Goedhart, 2019).

First, we tested whether the segmentation clock can be entrained to periods different and far from the endogenous period of ∼140 min, which we refer to as its free-running, natural period or $T_{o\,s\,c}$. To address this question, we modified the entrainment period from 120 to 180 min, while keeping the drug concentration and the pulse duration (i.e. 30 min/cycle) constant. Our results show that, while controls cycled close to $T_{o\,s\,c}$ (Figure 3A–C, Figure 3—figure supplement 1), the segmentation clock rhythm in DAPT-entrained samples closely adjusted to $T_{z\,e\,i\,t}$ over the specified range (Figure 3D, Figure 3—figure supplement 1, Figure 3—figure supplement 2). Hence, we were able to speed up and slow down the pace of the segmentation clock system using entrainment.

**Figure 3—figure supplement 1.**
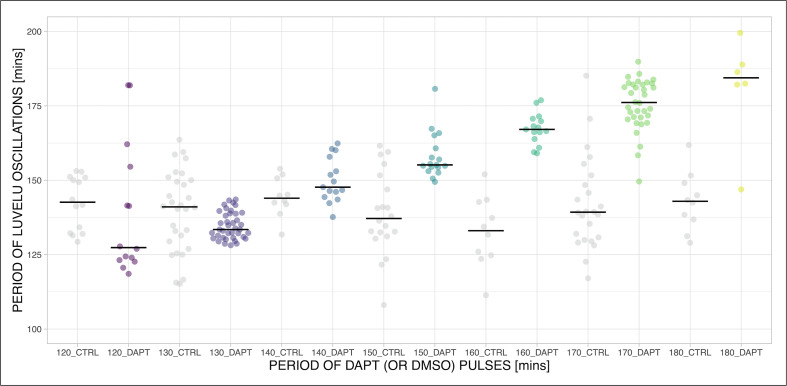
Speeding up and slowing down the period of the segmentation clock. The period of the segmentation clock can both be sped up and slowed down by modulating the period of DAPT pulses. Mean period from 650 to 850 min after start of the experiment of samples subjected to periodic pulses of 2 µM DAPT (or DMSO for controls). Each sample is represented as a dot, while the median of all samples is denoted as a solid horizontal line. The period of the DAPT (or DMSO) pulses is specified. 120 min: (CTRL: *n*=14 and *N*=3) and (DAPT: *n*=14 and *N*=3) with p-value = 0.064, 130 min: (CTRL: *n*=30 and *N*=8) and (DAPT: *n*=39 and *N*=10) with p-value = 0.003, 140 min: (CTRL: *n*=10 and *N*=3) and (DAPT: *n*=15 and *N*=3) with p-value = 0.272, 150 min: (CTRL: *n*=20 and *N*=4) and (DAPT: *n*=17 and *N*=4) with p-value = 0.001, 160 min: (CTRL: *n*=10 and *N*=3) and (DAPT: *n*=15 and *N*=3) with p-value <0.001, 170 min: (CTRL: *n*=24 and *N*=7) and (DAPT: *n*=34 and *N*=8) with p-value <0.001, 180 min: (CTRL: *n*=10 and *N*=2) and (DAPT: *n*=6 and *N*=1) with p-value = 0.001. Data were visualized using PlotsOfData (Postma and Goedhart, 2019). To calculate p-values, two-tailed test for absolute difference between medians was done via a randomization method using PlotsOfDifferences (Goedhart, 2019).

**Figure 3—figure supplement 2.**
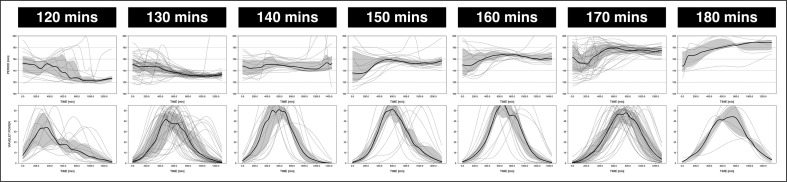
Locking of the segmentation clock to the period of the DAPT pulses. The period of the segmentation clock becomes locked to the period of the DAPT pulses. The period and wavelet power of the oscillations, obtained via wavelet analysis, are plotted across time. Each sample and their median are represented here as a dashed line and a solid line, respectively. The gray shaded area corresponds to the interquartile range. The period of the 2 µM DAPT pulses is specified. 120 min: *n*=14 and *N*=3, 130 min: *n*=39 and *N*=10, 140 min: *n*=15 and *N*=3, 150 min: *n*=17 and *N*=4, 160 min: *n*=15 and *N*=3, 170 min: *n*=34 and *N*=8, 180 min: *n*=6 and *N*=1. The period evolution plots for the 130 and 170 min conditions are the same as the period evolution plots for the 2 µM condition in Figure 4—figure supplement 1A and Figure 4B, respectively.

Notably, period-locking was less precise (i.e. higher standard deviation as shown in Table 1) with 120 and 180 min *zeitgeber* periods, a possible indication that the limit of entrainment range is approached at these conditions.

**Table 1.** Summary statistics on period-locking of the segmentation clock in E10.5 2D-assays to periodic pulses of 2 µM DAPT. This table summarizes the median, 95% confidence interval (CI) of the median, mean, standard deviation (SD), and standard error of the mean (SEM) of the segmentation clock in 2D-assays subjected to periodic pulses of 2 µM DAPT. These summary statistics were determined using PlotsOfData (Postma and Goedhart, 2019). A plot of these data is shown in Figure 3D.

Pulse period	*n*	*N*	Median, min	95% CI of median, min	Mean, min	SD, min	SEM, min
120 min	14	3	127.36	123.16–148.03	139.39	22.28	6.18
130 min	39	10	133.44	132.21–135.97	134.75	4.56	0.74
140 min	15	3	147.68	146.10–153.02	150.00	7.42	1.98
150 min	17	4	155.14	154.33–160.74	158.18	7.77	1.94
160 min	15	3	167.09	166.16–169.84	167.33	5.28	1.41
170 min	34	8	176.14	173.06–181.20	175.56	8.52	1.48
180 min	6	1	184.43	164.54–194.21	181.06	17.88	8.00

**Figure 4.**
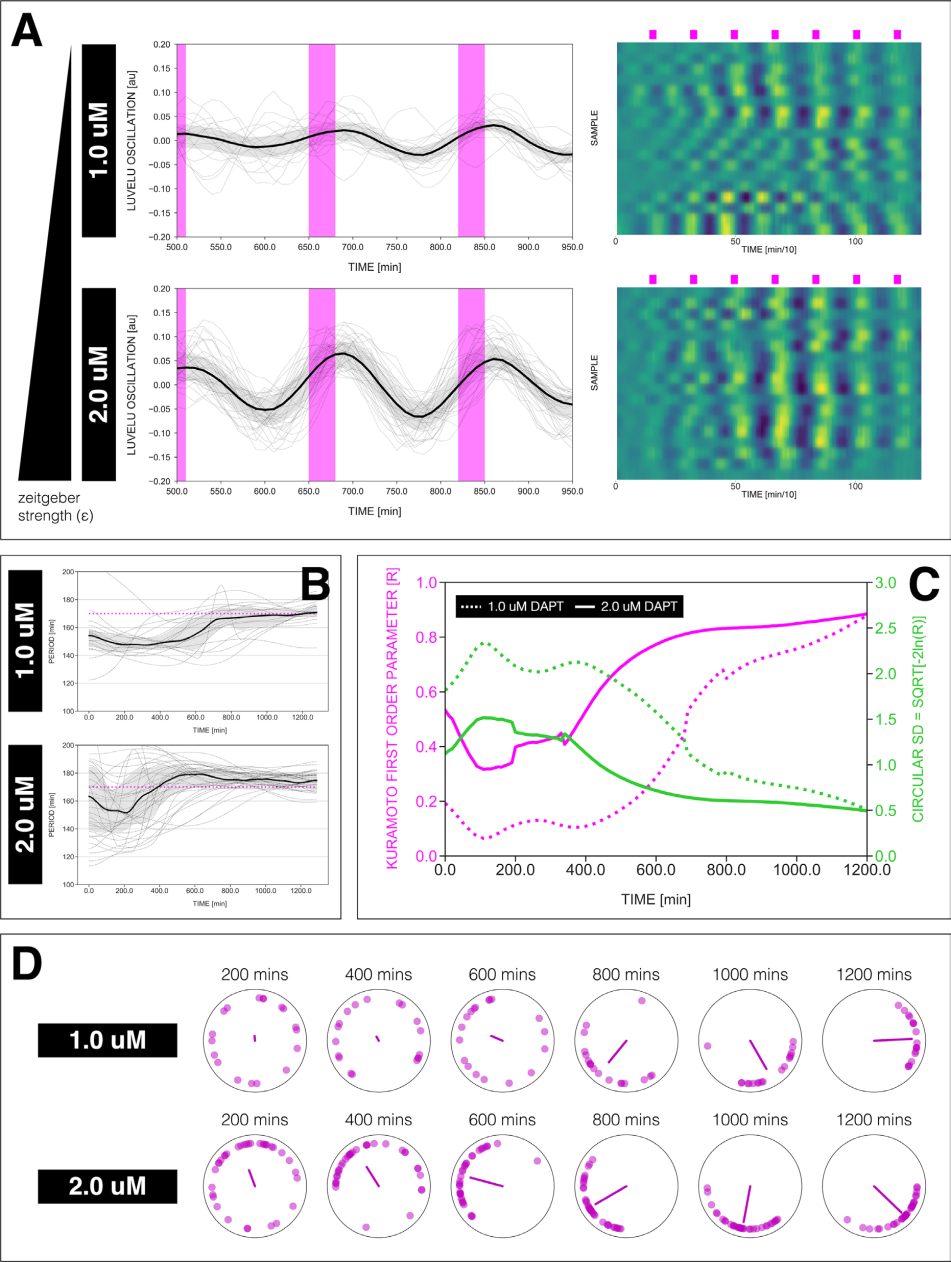
Effect of varying DAPT concentrations on entrainment dynamics. (**A**) Left: Detrended (via sinc filter detrending) timeseries of the segmentation clock in 2D-assays entrained to 170 min periodic pulses of either 1 or 2 µM DAPT, zoomed in from 500 to 950 min. Periodic pulses are indicated as magenta bars and the timeseries of each sample (for 1 µM: *n*=18 and *N*=5, for 2 µM: *n*=34 and *N*=8) is marked with a dashed line. The median of the oscillations is represented here as a solid line, while the gray shaded area denotes the interquartile range. Right: Detrended (via sinc filter detrending) timeseries of the segmentation clock in 2D-assays entrained to 170 min periodic pulses of either 1 µM (*n*=18 and *N*=5) or 2 µM (*n*=18 and *N*=8) DAPT represented as heatmaps, generated using PlotTwist (Goedhart, 2020). Periodic pulses are indicated as magenta bars. Each row corresponds to a sample. (**B**) Period evolution during entrainment, obtained from wavelet analysis. The period evolution for each sample and the median of the periods are represented here as a dashed line and a solid line, respectively. The gray shaded area corresponds to the interquartile range. The plot for the 2 µM condition is the same as the plot for the DAPT condition in Figure 3B. Magenta dashed line marks $T_{z\,e\,i\,t}$. (**C**) Evolution of first Kuramoto order parameter (R) in magenta and circular standard deviation (c⁢i⁢r⁢c⁢S⁢D) in green over time, showing change in coherence of multiple samples during the experiment. An R equal to 1.0 means that samples are in-phase. c⁢i⁢r⁢c⁢S⁢D is equal to $\sqrt{-2\,\text{ln}\,R}$. (**D**) Polar plots at different timepoints showing phase of each sample and their first Kuramoto order parameter, represented as a magenta dot along the circumference of a circle and a magenta line segment at the circle’s center, respectively. A longer line segment corresponds to a higher first Kuramoto order parameter, and thus to more coherent samples. The direction of the line denotes the vectorial average of the sample phases. Time is indicated as minutes elapsed from the start of the experiment.

We also tested the effect of varying DAPT concentration, as means of changing *zeitgeber* strength (ϵ in Figure 1), on entrainment dynamics. Synchronization theory predicts that *zeitgeber* strength correlates with the time it takes to reach period-locking (Pikovsky et al., 2001; Granada and Herzel, 2009). To test this prediction, we entrained samples with periodic DAPT pulses at fixed intervals of 170 min and varied drug concentration. We indeed found that the time needed to show period-locking was shortened in samples using higher DAPT concentrations (Figure 4A–B, Figure 4—figure supplement 1A). Additionally, as expected, higher drug concentrations also resulted in more robust entrainment, indicated by the quantifications of the first Kuramoto order parameter R, a measure for in-phase synchrony between samples (Figure 4C–D, Figure 4—figure supplement 1B, Figure 4—figure supplement 2) (see definition in *Appendix 1*, notice that R is the modulus of the mean field variable used in Riedel-Kruse et al., 2007).

**Figure 4—figure supplement 1.**
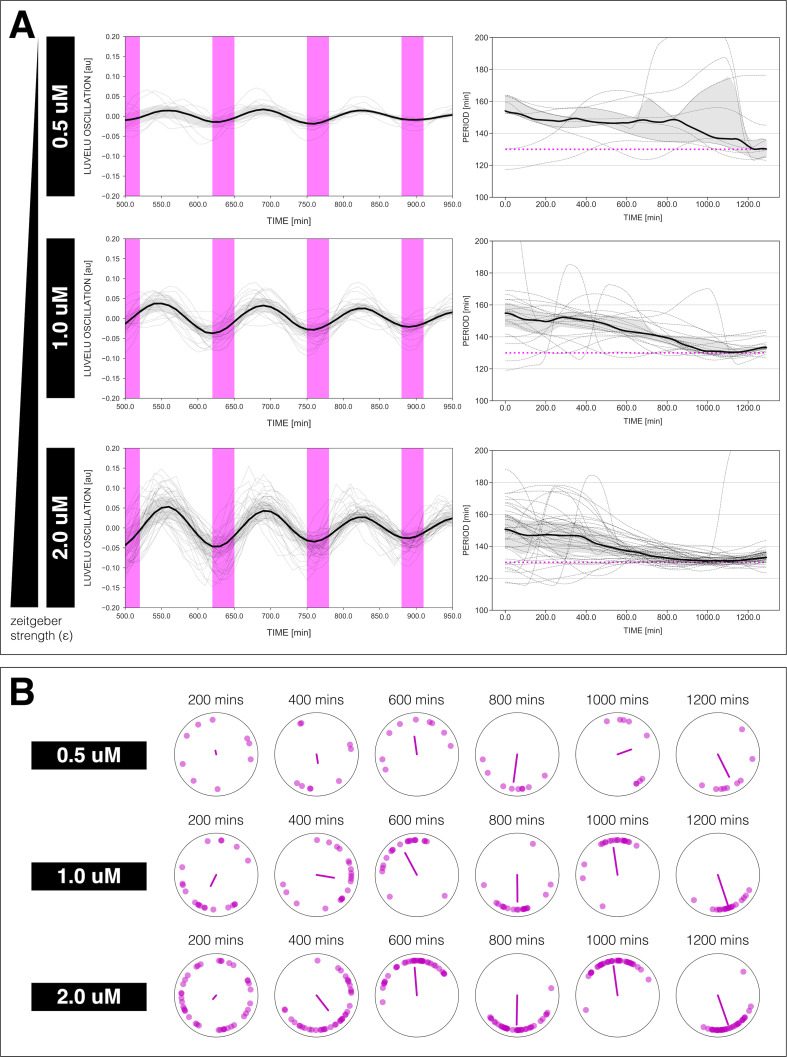
Changing the concentration of DAPT affects entrainment. Changing the concentration of DAPT, equivalent to changing *zeitgeber* strength, affects entrainment of the segmentation clock to 130 min periodic DAPT pulses. (**A**) Left: Detrended (via sinc filter detrending) timeseries of the segmentation clock in 2D-assays entrained to 130 min periodic pulses of either 0.5, 1, or 2 µM DAPT, zoomed in from 500 to 950 min. Periodic pulses are indicated as magenta bars and the timeseries of each sample (for 0.5 µM: *n*=9 and *N*=2, for 1 µM: *n*=20 and *N*=4, for 2 µM: *n*=39 and *N*=10) is marked with a dashed line. The median of the oscillations is represented here as a solid line, while the gray shaded area denotes the interquartile range. Right: Period evolution during entrainment, obtained from wavelet analysis. The period evolution for each sample and the median of the periods are represented here as a dashed line and a solid line, respectively. The gray shaded area corresponds to the interquartile range. Magenta dashed line marks $T_{z\,e\,i\,t}$. (**B**) Polar plots at different timepoints showing phase of each sample and their first Kuramoto order parameter, represented as a magenta dot along the circumference of a circle and a magenta line segment at the circle’s center, respectively. A longer line segment corresponds to a higher first Kuramoto order parameter, and thus to more coherent samples. The direction of the line denotes the vectorial average of the sample phases. Time is indicated as minutes elapsed from the start of the experiment.

**Figure 4—figure supplement 2.**
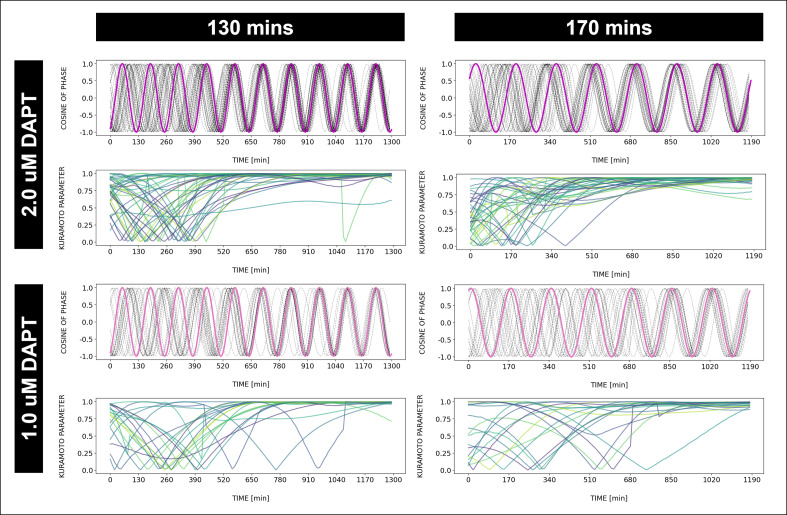
Dynamics and variability of entrainment. Dynamics and variability of entrainment. We represent the *zeitgeber* signal as a continuous uniformly increasing phase (‘*zeitgeber time*’) with period $T_{zeit}$. The initial condition for $\phi_{z\,e\,i\,t}$ is chosen so that the *zeitgeber* phase at the moment of the last pulse is matching the experimental entrainment phase for each $T_{z\,e\,i\,t}$. We plot the corresponding cos⁡ϕ⁢(t) for each sample (dotted lines) and the *zeitgeber* phase (solid lines). To quantify how well each sample is following the *zeitgeber*, we compute the Kuramoto parameter: $R_{j}\,\left(t\right)=\frac{1}{2}\,\left|\exp i\,\phi_{j}\,\left(t\right)+\exp i\,\phi_{z\,e\,i\,t}\,\left(t\right)\right|$, where $\phi_{j}\,\left(t\right)$ is the phase of sample j. Convergence to 1 of the Kuramoto parameter $R_{j}$ indicates the establishment of a stable phase relation between the sample and *zeitgeber* pulses, indicative of entrainment. As seen from the plots, at lower concentration of DAPT samples take a longer time on average to reach entrainment.

### Higher-order entrainment of the segmentation clock

**Figure 5.**
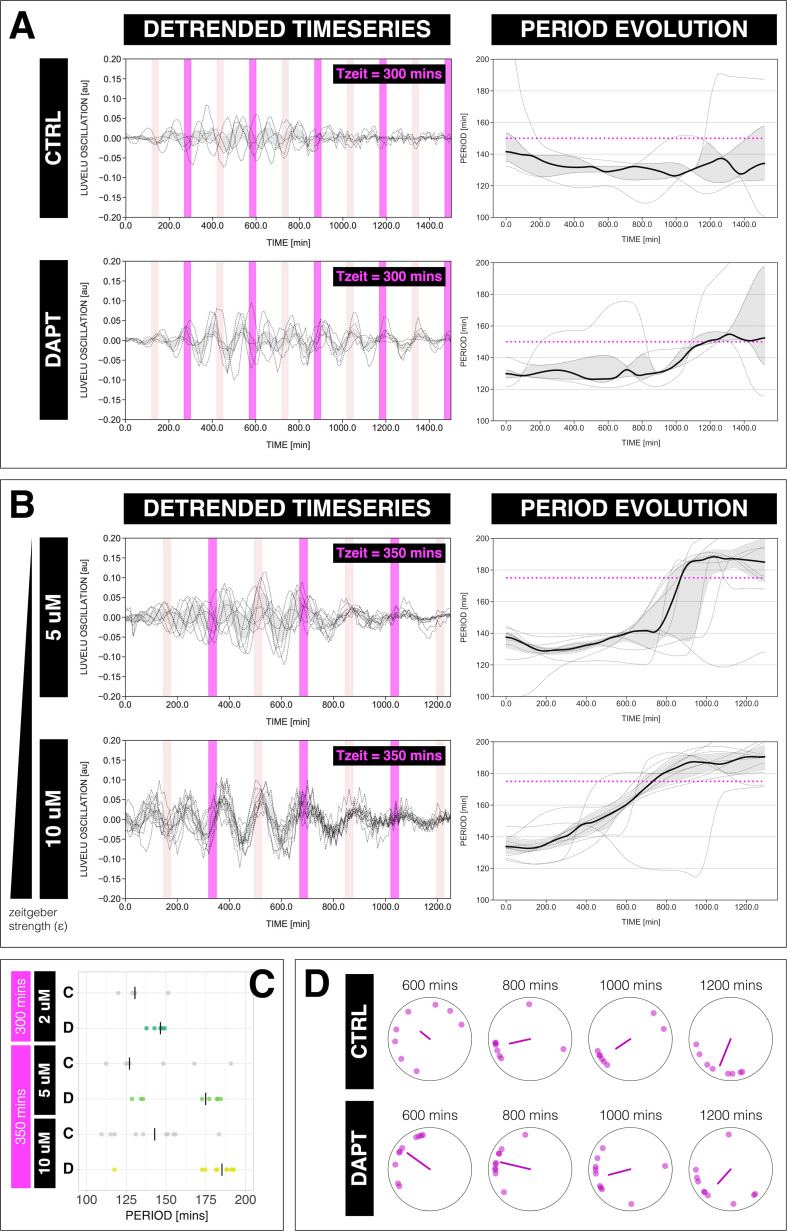
The segmentation clock can be entrained to a higher order. (**A**) Left: Detrended (via sinc filter detrending) timeseries of the segmentation clock in 2D-assays subjected to 300 min periodic pulses of 2 µM DAPT (or DMSO for controls). Periodic pulses are indicated as magenta bars and the timeseries of each sample (for CTRL: *n*=5 and *N*=1, for DAPT: *n*=5 and *N*=1) is marked with a dashed line. The gray shaded area denotes the interquartile range. Hypothetical pulses at half the *zeitgeber* period are indicated as light pink bars. Right: Period evolution during entrainment, obtained from wavelet analysis. The period evolution for each sample and the median of the periods are represented here as a dashed line and a solid line, respectively. The gray shaded area corresponds to the interquartile range. Magenta dashed line marks $0.5\,T_{z\,e\,i\,t}$. (**B**) Left: Detrended (via sinc filter detrending) timeseries of the segmentation clock in 2D-assays subjected to 350 min periodic pulses of either 5 µM DAPT or 10 µM DAPT. Periodic pulses are indicated as magenta bars and the timeseries of each sample (for 5 µM DAPT: *n*=8 and *N*=2, for 10 µM DAPT: *n*=10 and *N*=2) is marked with a dashed line. The gray shaded area denotes the interquartile range. Hypothetical pulses at half the *zeitgeber* period are indicated as light pink bars. Right: Period evolution during entrainment, obtained from wavelet analysis. The period evolution for each sample and the median of the periods are represented here as a dashed line and a solid line, respectively. The gray shaded area corresponds to the interquartile range. Magenta dashed line marks $0.5\,T_{z\,e\,i\,t}$. (**C**) Mean period either from 1000 to 1150 min (for $T_{z\,e\,i\,t}=300$ min) or from 800 to 950 min (for $T_{z\,e\,i\,t}=350$ min) after start of the experiment of samples subjected to periodic pulses of DAPT (or DMSO for controls). Each sample is represented as a dot, while the median of all samples is denoted as a solid vertical line. For 300 min 2 µM DAPT: (CTRL: *n*=5 and *N*=1) and (DAPT: *n*=5 and *N*=1) with p-value = 0.191, for 350 min 5 µM DAPT: (CTRL: *n*=7 and *N*=2) and (DAPT: *n*=8 and *N*=2) with p-value = 0.049, for 350 min 10 µM DAPT: (CTRL: *n*=10 and *N*=2) and (DAPT: *n*=10 and *N*=2) with p-value = 0.016. The period and concentration of the DAPT pulses are specified. Data were visualized using PlotsOfData (Postma and Goedhart, 2019). To calculate p-values, two-tailed test for absolute difference between medians was done via a randomization method using PlotsOfDifferences (Goedhart, 2019). (**D**) Polar plots at different timepoints showing the phase of each sample in (**A**) and the first Kuramoto order parameter, represented as a magenta dot along the circumference of a circle and a magenta line segment at the circle’s center, respectively. A longer line segment corresponds to a higher first Kuramoto order parameter, and thus to more coherent samples. The direction of the line denotes the vectorial average of the sample phases. Time is indicated as minutes elapsed from the start of the experiment.

In theory, a non-linear oscillator should be amenable not only to 1:1 entrainment, but also to higher-order n:m entrainment, in which n cycles of the endogenous oscillation lock to m cycles of the *zeitgeber* (Pikovsky et al., 2001; Mitarai et al., 2013). In practice, demonstration of higher-order entrainment is challenging due to the narrow permissive parameter region, that is, Arnold tongues are progressively narrower away from the 1:1 regime. Strikingly, we found experimental evidence for higher-order 2:1 entrainment (Figure 5). Samples entrained with either 300 or 350 min pulses showed evidence of 2:1 entrainment (Figure 5A–C), that is, the segmentation clock oscillated twice per each *zeitgeber* pulse and hence the segmentation clock rhythm adjusted to a period very close to 150 min for $T_{z\,e\,i\,t}=300$ min and approaching 175 min for $T_{z\,e\,i\,t}=350$ min (Figure 5C). We also found evidence for phase-locking during 2:1 entrainment, that is, a narrowing of the phase distribution among the individual samples (Figure 5D).

### The segmentation clock establishes a stable entrainment phase relative to the *zeitgeber*

**Figure 6.**
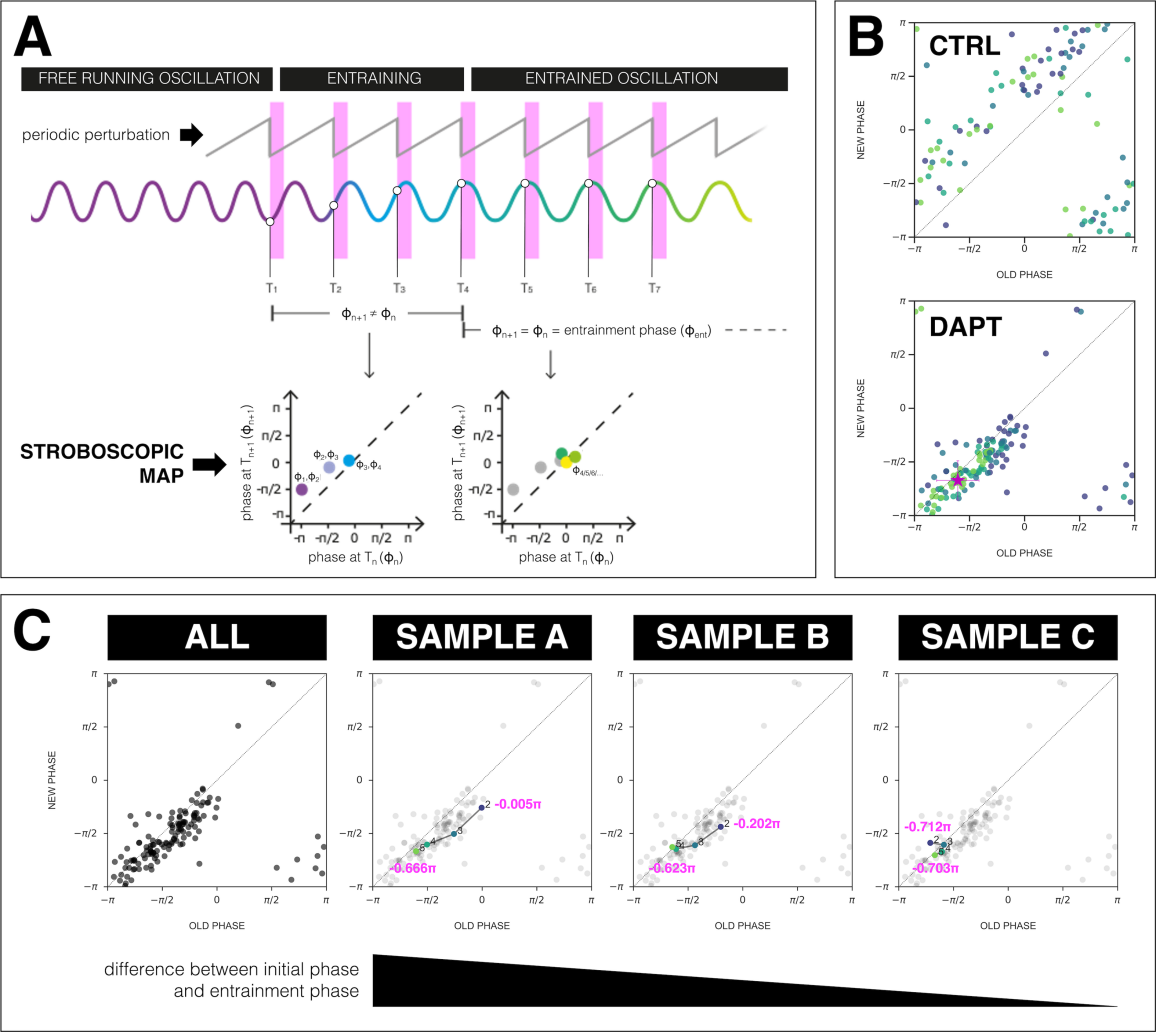
The segmentation clock establishes a stable phase relationship with the *zeitgeber*. (**A**) Schematic of how to generate a stroboscopic map, where the phase of the segmentation clock just before a DAPT pulse (old phase, $\phi_{n}$) is iteratively plotted against its phase just before the next pulse (new phase, $\phi_{n+1}$).The position of each point in a stroboscopic map thus denotes a stepwise change in phase of the segmentation clock as it undergoes entrainment to the *zeitgeber*. Upon entrainment and phase-locking, the new phase is equal to the old phase, thus marking a point that lies on the diagonal of the stroboscopic map. This point on the diagonal is the entrainment phase ($\phi_{e\,n\,t}$). Illustration by Stefano Vianello. (**B**) Stroboscopic map of samples subjected to 170 min periodic pulses of 2 µM DAPT (or DMSO for controls). Colors mark progression in time, from purple (early) to yellow (late). Note that while in control samples, points remain above the diagonal (reflecting that endogenous oscillations run faster than $T_{z\,e\,i\,t}=170$ min as shown in Figure 3B–C), in entrained samples, the measurements converge toward a point on the diagonal (i.e. the entrainment phase, $\phi_{e\,n\,t}$), showing phase-locking. This localized region marks the entrainment phase ($\phi_{e\,n\,t}$). This is highlighted with a magenta star, which corresponds to the centroid $\left(x_{c},y_{c}\right)$. The centroid was calculated from the vectorial average of the phases of the samples at the end of the experiment, where *x*_*c*_ = vectorial average of old phase, *y*_*c*_ = vectorial average of new phase. The spread of the points in the region is reported in terms of the circular standard deviation ($\sqrt{-2\,\text{ln}\,R}$, where R is the first Kuramoto order parameter). (**C**) Stroboscopic maps of the segmentation clock entrained to 170 min periodic pulses of 2 µM DAPT (*n*=34 and *N*=8), for all samples (ALL) and for three individual samples (SAMPLE A, SAMPLE B, SAMPLE C). The numbers and colors (from purple to yellow) denote progression in time. The initial phase (old phase at point 2) and the entrainment phase (new phase at point 5) of each sample are specified.

We next analyzed the phase-locking behavior between the segmentation clock and the *zeitgeber*. According to dynamical systems theory, phase-locking, that is, the entrainment phase ($\phi_{e\,n\,t}$), can be characterized as an attractor, that is, it is a stable fixed point (Cross and Siggia, 2005; Izhikevich, 2006). To quantify the entrainment phase, we plotted the data as stroboscopic maps (Figure 6A), which take a snapshot of the segmentation clock phase at regular intervals based on *zeitgeber* pulses (Isomura et al., 2017; Cross and Siggia, 2005; Izhikevich, 2006). Stroboscopic maps enable determination of $\phi_{e\,n\,t}$ as a stable fixed point that lies on the diagonal, where there is phase-locking. Plotting the stroboscopic maps shows that in samples entrained to periodic pulses of DAPT the segmentation clock phase dynamics converge to a region on the diagonal, which marks $\phi_{e\,n\,t}$ (Figure 6B). Such convergence toward a stable entrainment phase is further highlighted by looking at consecutive pulses and the corresponding phase trajectories of individual samples. As exemplified in Figure 6C and as previously theoretically predicted (Granada and Herzel, 2009; Granada et al., 2009), at the same *zeitgeber* strength and *zeitgeber* period, faster (or slower) convergence toward this fixed point (i.e. entrainment) was achieved when the initial phase of the endogenous oscillation ($\phi_{i\,n\,i\,t}$) was closer (or farther) to $\phi_{e\,n\,t}$.

### The phase of entrainment varies according to *zeitgeber* period

One fundamental property of entrained oscillatory systems is that its phase of entrainment varies as a function of the detuning, that is, the period mismatch between the endogenous oscillator and the entrainment period (Pittendrigh and Bruce, 2015; Aschoff and Pohl, 1978; Rémi et al., 2010; Bordyugov et al., 2015; Granada et al., 2013). In addition, theoretical studies have supported a ‘180 degree’ rule (Granada et al., 2013), stating that the phase of entrainment varies by half a cycle (180° or π) within the range of permissible *zeitgeber* entrainment periods, $T_{z\,e\,i\,t}$ (Aschoff and Pohl, 1978; Granada et al., 2013).

**Figure 7.**
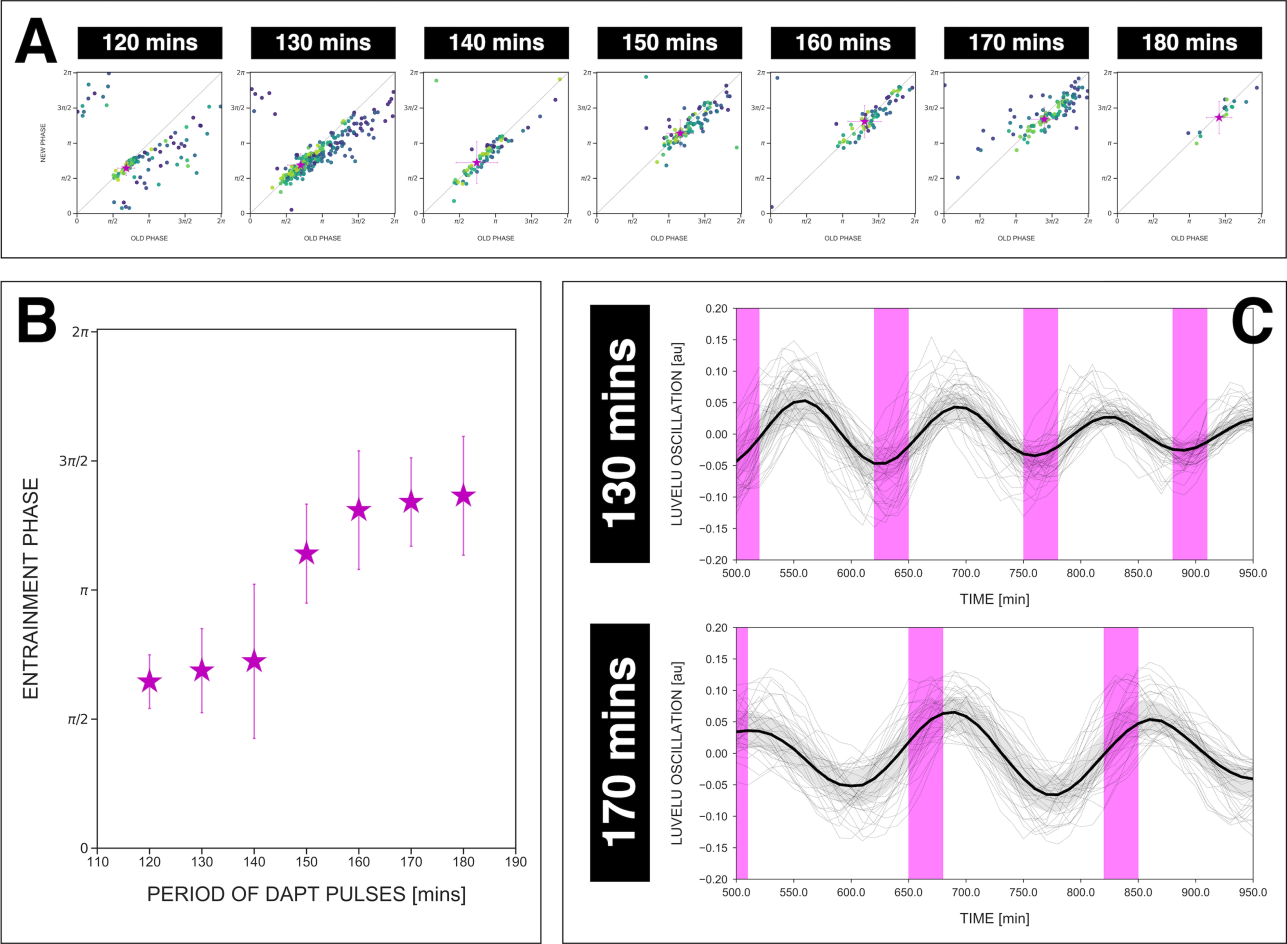
The entrainment phase varies according to *zeitgeber* period within a range of π. (**A**) Stroboscopic maps for different periods of DAPT pulses (i.e. *zeitgeber* periods) placed next to each other. In these maps, only samples that were phase-locked by the end of the experiment are considered (for 120 min: *n*=13/14 and *N*=3/3, for 130 min: *n*=38/39 and *N*=10/10, for 140 min: *n*=10/15 and *N*=3/3, for 150 min: *n*=16/17 and *N*=4/4, for 160 min: *n*=11/15 and *N*=3/3, for 170 min: *n*=28/34 and *N*=8/8, for 180 min: *n*=4/6 and *N*=1/1). Here, a sample was considered phase-locked if the difference between its phase at the time of the final drug pulse considered and its phase one drug pulse before is less than π/8. The localized region close to the diagonal in each map marks the entrainment phase ($\phi_{e\,n\,t}$) for that *zeitgeber* period. This is highlighted with a magenta star, which corresponds to the centroid $\left(x_{c},y_{c}\right)$. The centroid was calculated from the vectorial average of the phases of the samples at the end of the experiment, where *x*_*c*_ = vectorial average of old phase, *y*_*c*_ = vectorial average of new phase. The spread of the points in the region is reported in terms of the circular standard deviation ($\sqrt{-2\,\text{ln}\,R}$, where R is the first Kuramoto order parameter). The *zeitgeber* period is indicated above the maps. Colors mark progression in time, from purple to yellow. Stroboscopic maps of all samples and their respective controls are shown in Figure 7—figure supplement 1. Drug concentration and drug pulse duration were kept constant between experiments at 2 µM and 30 min/cycle, respectively. Phase 0 is defined as the peak of the oscillation. (**B**) Entrainment phase ($\phi_{e\,n\,t}$) at different *zeitgeber* periods, each calculated from the vectorial average of the phases of phase-locked samples at the time corresponding to last considered DAPT pulse. The spread of $\phi_{e\,n\,t}$ between samples is reported in terms of the circular standard deviation ($\sqrt{-2\,\text{ln}\,R}$, where R is the first Kuramoto order parameter). (**C**) Detrended (via sinc filter detrending) timeseries of the segmentation clock in 2D-assays entrained to either 130 or 170 min periodic pulses of 2 µM DAPT, zoomed in from 500 to 950 min. Periodic pulses are indicated as magenta bars and the timeseries of each sample (for 130 min: *n*=39 and *N*=10, for 170 min: *n*=34 and *N*=8) is marked with a dashed line. The median of the oscillations is represented here as a solid line, while the gray shaded area denotes the interquartile range. The detrended timeseries for the 130 and 170 min conditions are the same as the detrended timeseries for the 2 µM condition in Figure 4—figure supplement 1A and Figure 4A, respectively. The full detrended timeseries for the 170 min condition can be seen in Figure 3A.

To test these theoretical predictions, we quantified $\phi_{e\,n\,t}$ across a wide range of detuning, that is, from 120 to 180 min *zeitgeber* period. We found that $\phi_{e\,n\,t}$, based on the centroid localization close to the diagonal in the stroboscopic maps, gradually changed its position as *zeitgeber* period, that is, detuning, was varied (Figure 7A, Figure 7—figure supplement 1). From 120 to 180 min entrainment periods, $\phi_{e\,n\,t}$ systematically shifted from ∼π/2 to ∼3⁢π/2 (Figure 7A–B), spanning a range of almost π (Figure 7B–C). Hence, as predicted by theory, our results show that the segmentation clock phase of entrainment varies as a function of the detuning relative to *zeitgeber* pulses.

**Figure 7—figure supplement 1.**
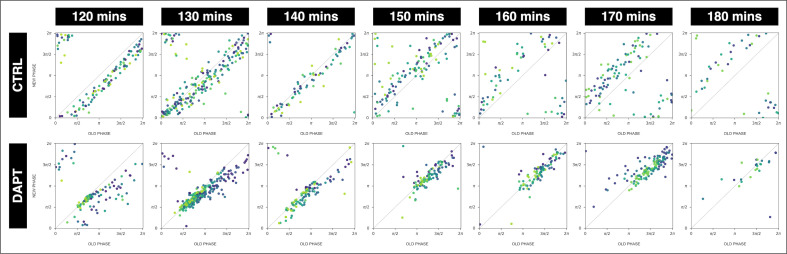
Stable phase relationship with the DAPT pulses. The segmentation clock establishes a stable phase relationship with the periodic DAPT pulses. Stroboscopic maps summarizing phase dynamics when the segmentation clock was subjected to periodic pulses of 2 µM DAPT (or DMSO for controls). The period of the DAPT (or DMSO) pulses is specified. Colors mark progression in time, from purple to yellow. 120 min: (CTRL: *n*=14 and *N*=3) and (DAPT: *n*=14 and *N*=3), 130 min: (CTRL: *n*=30 and *N*=8) and (DAPT: *n*=39 and *N*=10), 140 min: (CTRL: *n*=10 and *N*=3) and (DAPT: *n*=15 and *N*=3), 150 min: (CTRL: *n*=20 and *N*=4) and (DAPT: *n*=17 and *N*=4), 160 min: (CTRL: *n*=10 and *N*=3) and (DAPT: *n*=15 and *N*=3), 170 min: (CTRL: *n*=24 and *N*=7) and (DAPT: *n*=34 and *N*=8), 180 min: (CTRL: *n*=10 and *N*=2) and (DAPT: *n*=6 and *N*=1).

**Figure 7—figure supplement 2.**
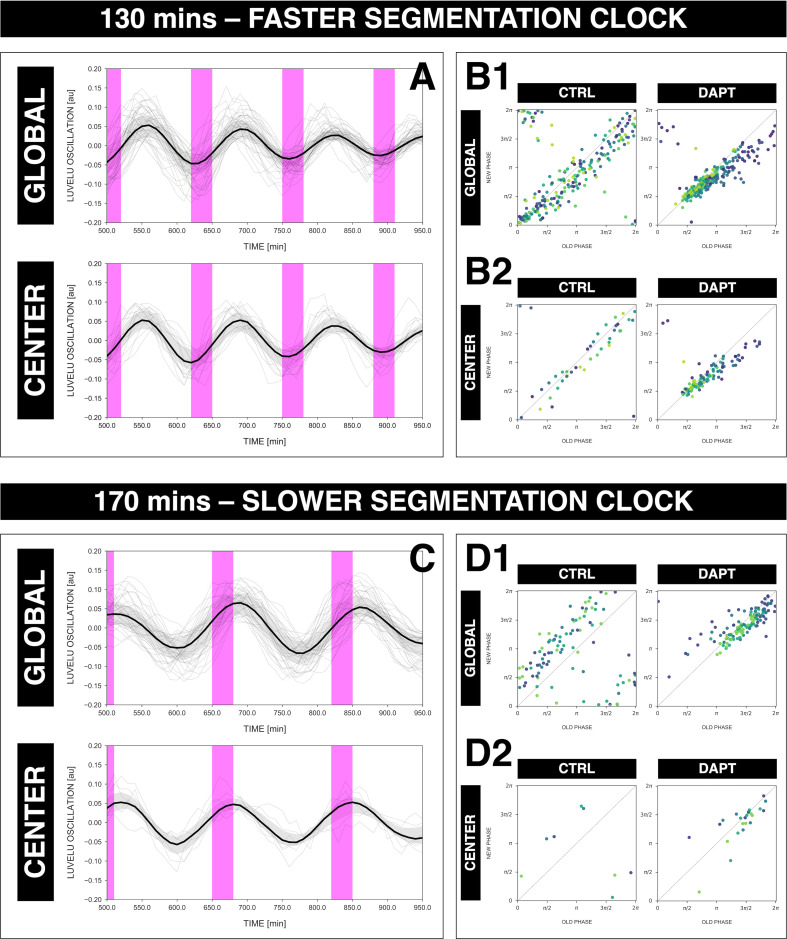
Rhythm of the segmentation clock matches center of 2D-assays. The rhythm of the segmentation clock matches the rhythm of oscillations in the center of 2D-assays (i.e. the posterior presomitic mesoderm [PSM]) regardless whether the clock is sped up or slowed down. (**A**) Detrended timeseries (via sinc filter detrending) of either the segmentation clock (global region of interest [ROI], ‘GLOBAL’) or oscillations in the center of 2D-assays (‘CENTER’) entrained to 130 min periodic pulses of 2 µM DAPT, zoomed in from 500 to 950 min. Periodic pulses are indicated as magenta bars and the timeseries of each sample (for GLOBAL: *n*=39 and *N*=10, for CENTER: *n*=15 and *N*=7) is marked with a dashed line. The median of the oscillations is represented here as a solid line, while the gray shaded area denotes the interquartile range. The plot for GLOBAL is the same as that for the 130 min condition in Figure 7C. (**B**) Stroboscopic maps of either the segmentation clock GLOBAL, (**B1**) or oscillations in the center of 2D-assays CENTER, (**B2**) subjected to 130 min periodic pulses of 2 µM DAPT, with their respective controls (subjected to periodic pulses of DMSO). Colors mark progression in time, from purple to yellow. (**C**) Detrended timeseries (via sinc filter detrending) of either the segmentation clock (GLOBAL) or oscillations in the center of 2D-assays (CENTER) entrained to 170 min periodic pulses of 2 µM DAPT, zoomed in from 500 to 950 min. Periodic pulses are indicated as magenta bars and the timeseries of each sample (for GLOBAL: *n*=34 and *N*=8, for CENTER: *n*=6 and *N*=5) is marked with a dashed line. The median of the oscillations is represented here as a solid line, while the gray shaded area denotes the interquartile range. The plot for GLOBAL is the same as that for the 170 min condition in Figure 7C. (**D**) Stroboscopic maps of either the segmentation clock GLOBAL, (**D1**) or oscillations in the center of 2D-assays CENTER, (**D2**) subjected to 170 min periodic pulses of 2 µM DAPT, with their respective controls (subjected to periodic pulses of DMSO). Colors mark progression in time, from purple to yellow.

**Figure 7—figure supplement 3.**
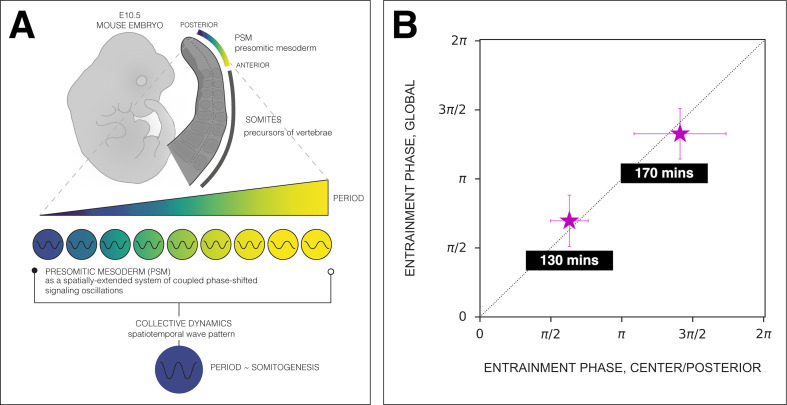
Phase of the segmentation clock matches center of 2D-assays. The phase of oscillations in the center of 2D-assays (∼posterior presomitic mesoderm [PSM]) matches the phase of the segmentation clock. (**A**) Scheme of the phase-shifted oscillations along the AP axis of the PSM and its correlation to the segmentation clock. Illustration by Stefano Vianello. (**B**) Comparison of the phase relationship between the periodic DAPT pulses and either the segmentation clock rhythm (measured in global region of interest [ROI], ‘GLOBAL’) or the rhythm in the center of 2D-assays (equivalent to posterior PSM, ‘CENTER/POSTERIOR’). The entrainment phase was calculated from the vectorial average of the phases of all samples at the time corresponding to the last DAPT pulse considered. The spread of sample phases is reported in terms of the circular standard deviation ($\sqrt{-2\,l\,n\,R}$, where *R* is the first Kuramoto order parameter). 130 min: (GLOBAL: *n*=39 and *N*=10) and (CENTER: *n*=15 and *N*=7), 170 min: (GLOBAL: *n*=34 and *N*=8) and (CENTER: *n*=6 and *N*=5).

It was previously documented that the period of tissue segmentation matches the period of oscillations localized in the posterior PSM, that is, center of of the 2D-assays (Tsiairis and Aulehla, 2016; Soza-Ried et al., 2014). We were then curious whether or not the dynamics from our coarse-grained analysis match that for oscillations in the center of the 2D-assays, and if the behavior of oscillations in this localized region also exhibits dependence to *zeitgeber* period. To study this, we compared the timeseries acquired from (a) global ROI spanning the entire field of view and from (b) a circular ROI restricted at the center of the 2D-assays (‘center ROI’, radius: 25 pixels, 1 pixel = 1.38 µm). Upon comparison, we noted that the oscillations at the center of 2D-assays adjusted its rhythm to the *zeitgeber* with strong correspondence to the coarse-grained oscillator (Figure 7—figure supplement 2), regardless of whether the segmentation clock was sped up (Figure 7—figure supplement 2A–B) or slowed down (Figure 7—figure supplement 2C–D). Moreover, the phase-locking between the *zeitgeber* and either the segmentation clock or the oscillations at the center of the samples are similar (Figure 7—figure supplement 2B1–B2, D1–D2, Figure 7—figure supplement 3). Concomitantly, for oscillations in the center of 2D-assays (i.e. corresponding to the posterior PSM), the entrainment phase at $T_{z\,e\,i\,t}=130$ min is different to the entrainment phase at $T_{z\,e\,i\,t}=170$ min (Figure 7—figure supplement 3B), here too exemplifying the effect of *zeitgeber* period on the phase of entrainment.

### PRCs derivation and period change

Building on our finding that the phase of entrainment varies as a function of detuning (Figure 7B), our goal was to extract the quantitative information embedded in this dynamic behavior. This is possible since the phase of entrainment dynamics reflect, at a quantitative level, the fundamental properties of a dynamical system that responds to external perturbations. This behavior, in turn, can usually be captured with a single function, the PRC (Izhikevich, 2006; Granada et al., 2013). The PRC describes the change of phase induced by a perturbation, and a priori depends on both the nature of the perturbation received and the phase of the cycle. Because of the direct dependence of the phase of entrainment dynamics on the PRC, our goal was to use the experimental data to gain insight into the segmentation clock PRC.

We follow Izhikevich, 2006, to define a generalized PRC not tied to the shape or duration of the perturbation. Without loss of generality, we then model perturbations in form of pulses of amplitude ϵ (Pikovsky et al., 2001) and write:

(1) $$PRC(\phi_{pulse},\epsilon )=\phi_{afterpulse}(\phi_{pulse},\epsilon )-\phi_{pulse}$$

where $\phi_{\text{pulse}}$ is the phase of the segmentation clock *on the cycle* at the moment of the pulse, and $\phi_{\text{after pulse}}$ the phase after the pulse (which can be defined even if the system transiently moves outside of the limit cycle, via isochrons, see Winfree, 1967). Amplitude ϵ is a dimensionless parameter, quantifying *zeitgeber* strength in the mathematical formalism we develop below (see *Appendix 1, section Arnold tongue computations* to see how we relate ϵ to DAPT concentrations). If, following a perturbation, the oscillator relaxes quickly toward the limit cycle, one can use the PRC to compute response to periodic pulses with period $T_{z\,e\,i\,t}$. The sequence of phases at each pulse is then given by the stroboscopic map, introduced in Figure 6A:

(2) $$\phi_{n+1}=\left(\phi_{n}+\text{PRC}(\phi_{n},\epsilon )+2\pi \frac{T_{zeit}}{T_{osc}}\right)\;mod\;2\pi$$

and 1:1 entrainment occurs when this stroboscopic map converges toward a single fixed point $\phi_{e\,n\,t}$ for a given $T_{z\,e\,i\,t}$. When $T_{z\,e\,i\,t}$ is varied, different phases of entrainment $\phi_{e\,n\,t}\,\left(T_{z\,e\,i\,t}\right)$ are observed, here plotted in Figure 7B.

To derive the PRC directly from the experimental stroboscopic maps, we invert Equation 2 into:

(3) $$\text{PRC}\,\left(\phi_{n},\epsilon \right)=\left(\phi_{n+1}-\phi_{n}-2\,\pi \,\frac{T_{z\,e\,i\,t}}{T_{o\,s\,c}}\right)\;\mathrm{mod}\;2\,\pi$$

By estimating $\phi_{n},\phi_{n+1}$ for a given $T_{z\,e\,i\,t}$, one can estimate the PRC. The advantage of this approach is that it allows to estimate the PRC at any observed phase at the pulse $\phi_{n}$ , even far from the entrainment phase $\phi_{e\,n\,t}$. Notice, however, that phases far from $\phi_{e\,n\,t}$ will be only sampled over the first few pulses (so with possibly much noise) while conversely, $\phi_{e\,n\,t}$ will be quickly oversampled (and as such better defined statistically).

**Figure 8.**
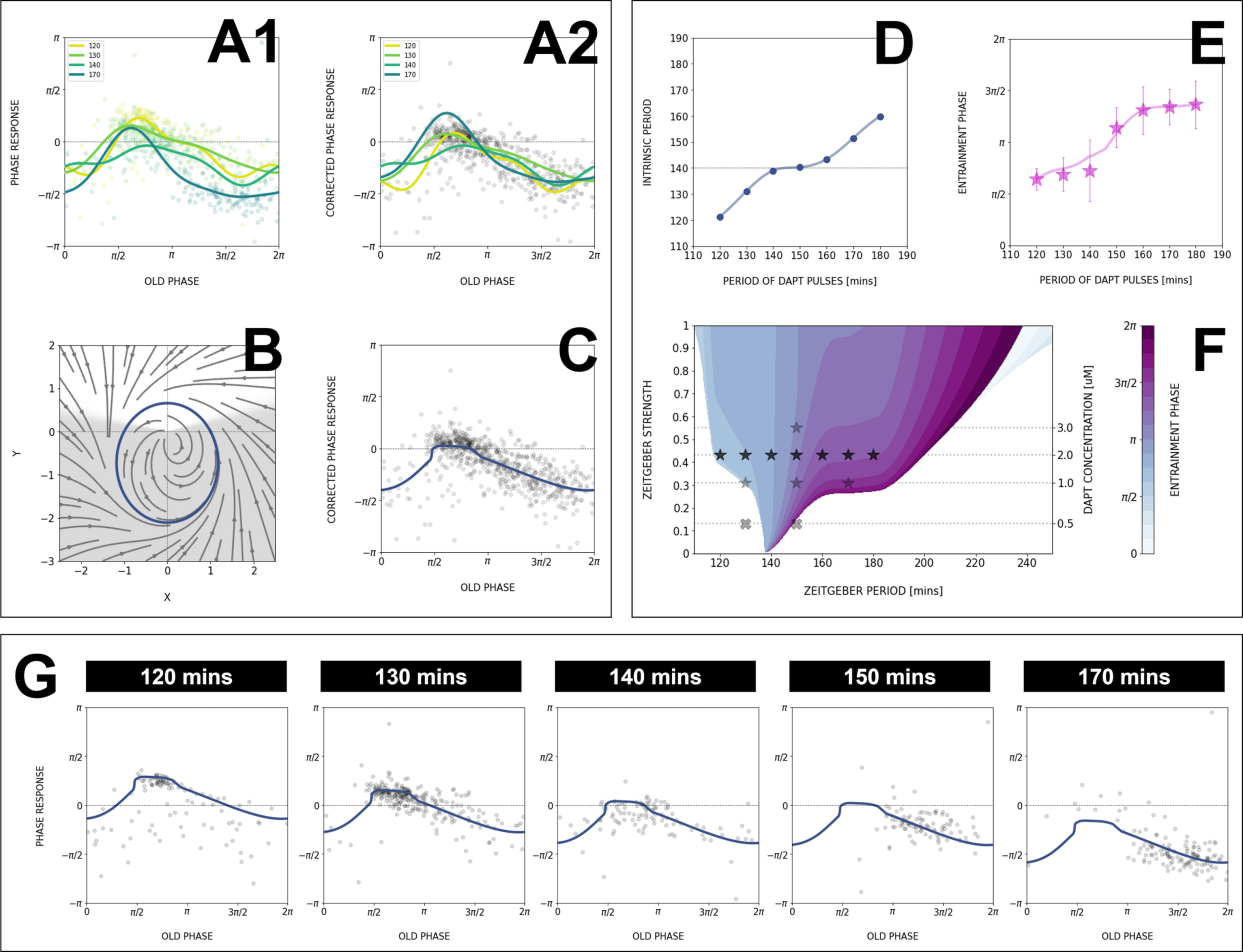
Modeling the segmentation clock entrainment response. (**A**) Phase response curve (PRC) from the data for different *zeitgeber* periods. (1) PRCs calculated at different $T_{z\,e\,i\,t}$ (points) and Fourier series fitted to them (lines). (2) Original PRCs are shifted vertically to collapse the data points on one curve. (**B**) Oscillator model, optimized by fitting the vertically shifted PRC. The limit cycle is an ellipse (blue) with eccentricity λ=0.5, the region with speeding up $s_{*}=5.6$ is shaded. (**C**) Optimized model PRC (line) overlaid to the vertically shifted data points. (**D**) Modeled intrinsic period $T_{o\,s\,c}$ as a function of entrainment period $T_{z\,e\,i\,t}$. Points were inferred from the PRCs data in A1 by matching the detuning and entrainment phase $\phi_{e\,n\,t}$, the function was interpolated with cubic splines. (**E**) Entrainment phase $\phi_{e\,n\,t}$ from the model with changing intrinsic period (line) agrees with the experimental data (points and error bars). Experimental data are the same as those in Figure 7B. (**F**) 1:1 Arnold tongue and isophases calculated with the model. Stars correspond to experimental data $\left(T_{z\,e\,i\,t},\epsilon \right)$ with observed entrainment, in agreement with $\phi_{e\,n\,t}$ data for different DAPT concentrations (Figure 8—figure supplement 4A). Black stars represent the experiments used for optimizing the model. Crosses correspond to experimental conditions with no entrainment. (**G**) Fit of the model (solid lines) overlaid with experimental phase differences (dots) at different periods $T_{z\,e\,i\,t}$.

Figure 8A1 shows the PRC computed from the data as well as Fourier series fits for different entrainment periods. PRCs computed for different entrainment periods have a similar shape, with similar locations for maxima and minima. Strikingly, those PRCs are not sinusoidal but essentially 0 or strongly negative, an unusual situation from a dynamical systems theory standpoint associated with special classes of oscillators (see more details below and in Discussion). However, contrary to theoretical predictions, the inferred PRCs at different entrainment periods do not overlap and appear shifted vertically as $T_{z\,e\,i\,t}$ is changed.

**Figure 8—figure supplement 1.**
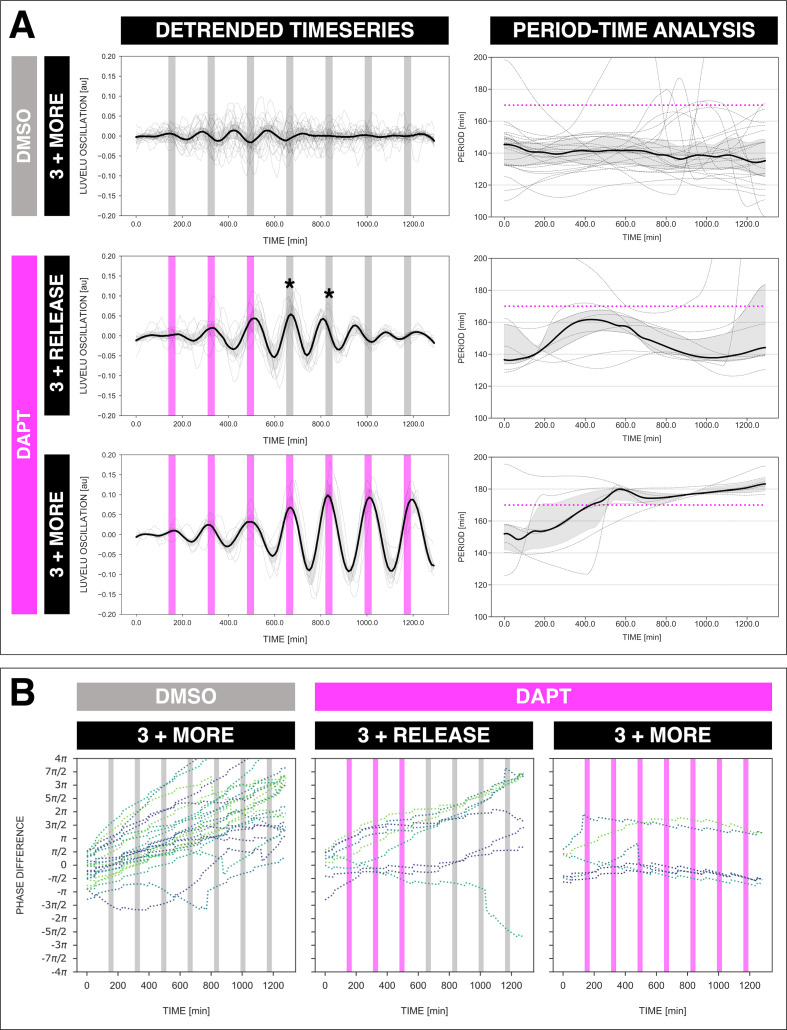
The segmentation clock keeps its adjusted rhythm a few cycles after release from the *zeitgeber.* The segmentation clock keeps its adjusted rhythm even a few cycles after release from DAPT pulses. (**A**) Left: Detrended (via sinc filter detrending) timeseries of the segmentation clock in 2D-assays subjected to 170 min periodic pulses of DMSO (gray bars) and/or 2 µM DAPT (magenta bars). The timeseries of each sample (for continuous DMSO pulses: *n*=24 and *N*=7, for three DAPT pulses and then release: *n*=9 and *N*=2, for continuous DAPT pulses: *n*=6 and *N*=2) is marked with a dashed line. The median of the oscillations is represented here as a solid line, while the gray shaded area denotes the interquartile range. Right: Period evolution during entrainment, obtained from wavelet analysis. The period evolution for each sample and the median of the periods are represented here as a dashed line and a solid line, respectively. The gray shaded area corresponds to the interquartile range. Magenta dashed line marks $T_{z\,e\,i\,t}$. Data for the continuous DMSO pulses are the same as the controls in Figure 3A–B. (**B**) Phase difference between the segmentation clock and the drug pulses. Note that a phase of −π/2 is equivalent to a phase of 3*π*/2, and a phase of 0 is equivalent to a phase of 2*π*. Periodic pulses of DMSO and 2 µM DAPT are indicated as gray bars and as magenta bars, respectively. Each sample within each condition is marked with different colors.

**Figure 8—figure supplement 2.**
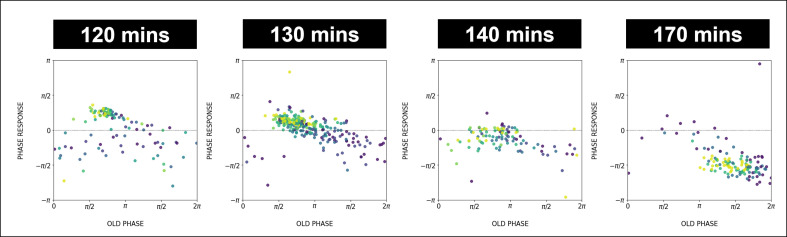
Phase response as a function of time. We use the color code for pulse number similar to Figure 6 (from purple for early pulses to yellow for later pulses). In the phase response curve (PRC) from the 120 min (170 min) experiment later data points are located higher (lower) than earlier data points, consistent with the change of the intrinsic period of oscillations during entrainment. The adjustment of the intrinsic period decreases the detuning, causing the vertical shift of the phase response curve.

**Figure 8—figure supplement 3.**
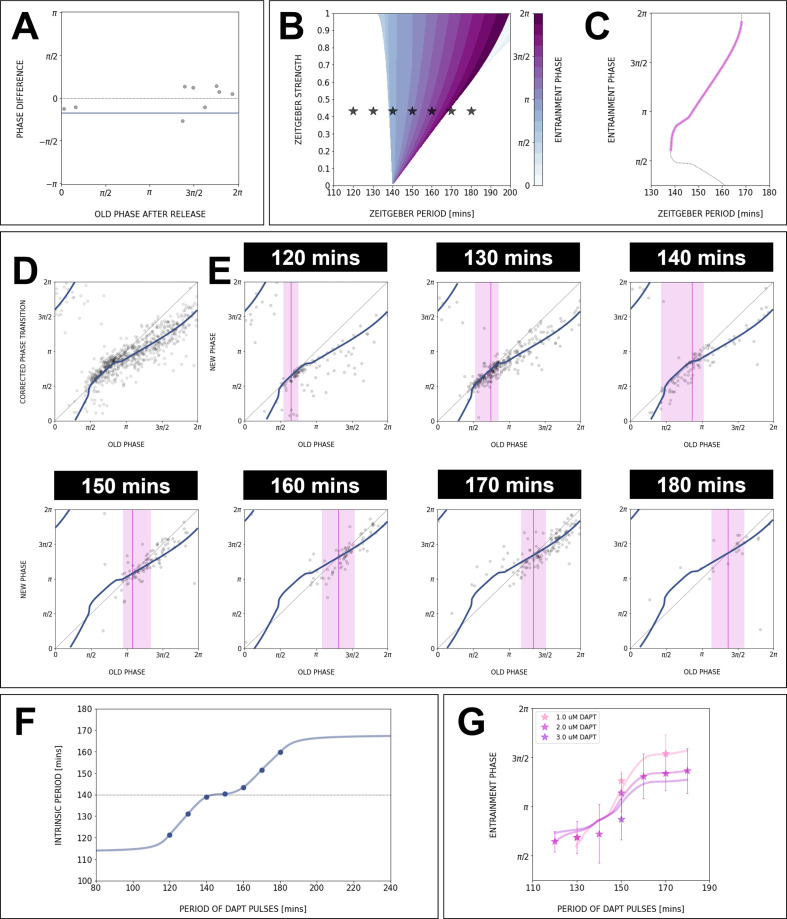
The model captures phase response curve (PRC)/phase transition curve (PTC), stroboscopic maps, and entrainment phase. Data for the PRC/PTC, stroboscopic maps, and entrainment phase can be fully captured with our model and changing intrinsic period. (**A**) Stroboscopic map data from release experiments allows to estimate the intrinsic oscillation period during entrainment. With $T_{z\,e\,i\,t}=170$ min and $T_{o\,s\,c}=150$ min, the phase difference between the end and the beginning of the first full cycle with no perturbations is on average 0 (2⁢π). The data points are from different release experiments with $T_{z\,e\,i\,t}=170$ min. If the natural period of 140 min is used, the average phase difference is -0.17⁢π (solid line). (**B, C**) Constant intrinsic period $T_{o\,s\,c}$ is not consistent with experimental data: (**B**) The 1:1 Arnold tongue, computed using our model and $T_{o\,s\,c}=140$ min, is much narrower than the experimental entrainment range. Stars correspond to experimental conditions ($T_{o\,s\,c}$, A) where entrainment was observed. (**C**) Entrainment phase $\phi_{e\,n\,t}\,\left(T_{z\,e\,i\,t}\right)$ numerically computed with the optimized PRC from Figure 8C, assuming constant $T_{o\,s\,c}=$ 140 min (which also corresponds to a cross-section of the isophases in B). Clearly, the slope of the curve is much higher than in the data. For comparison, we also plot the Figure 8C PRC rotated by π/2, showing perfect overlap. (**D**) PTC of the optimized model (equivalent to the PRC shown in Figure 8C). (**E**) Using the PTC from D, we fit the stroboscopic maps data for all periods $T_{z\,e\,i\,t}$ by choosing the $T_{o\,s\,c}(T_{z\,e\,i\,t}$) that gives the right detuning. The narrow magenta lines indicate the entrainment phase, the fixed point of each fitted stroboscopic map. The shaded magenta regions show the experimental range for entrainment phase. (**F**) Extrapolation of the intrinsic oscillator period $T_{o\,s\,c}$ as a function of zeitgeber period $T_{z\,e\,i\,t}$. (**G**) Cross-sections of the isophases in Figure 8F, calculated with the model and the extrapolated curve for $T_{o\,s\,c}\,\left(T_{z\,e\,i\,t}\right)$, give excellent agreement with the entrainment phase data for different concentrations of DAPT.

**Figure 8—figure supplement 4.**
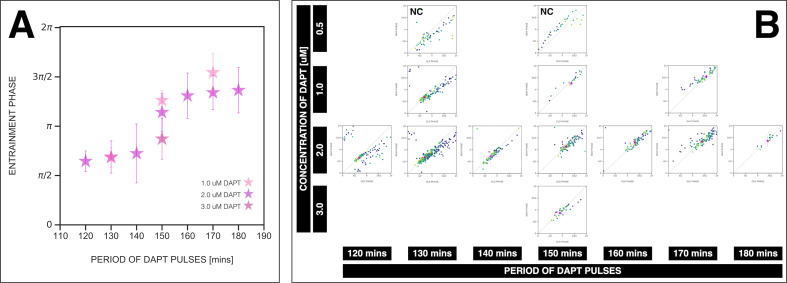
Zeitgeber period and strength affect the entrainment phase. Zeitgeber period and zeitgeber strength affect the entrainment phase of the segmentation clock. (**A**) Entrainment phase at different periods of DAPT pulses (i.e. zeitgeber period) and different drug concentrations (i.e. zeitgeber strength). Entrainment phase ($\phi_{e\,n\,t}$) was calculated from the vectorial average of the phases of phase-locked samples at the time corresponding to last considered DAPT pulse. A sample was considered phase-locked if the difference between its phase at the time of the final drug pulse considered and its phase one drug pulse before is less than π/8 – for 120 min: (2 µM: *n*=13/14 and *N*=3/3), for 130 min: (1 µM: *n*=17/20 and *N*=4/4), (2 µM: *n*=38/39 and *N*=10/10), for 140 min: (2 µM: *n*=10/15 and *N*=3/3), for 150 min: (1 µM: *n*=3/4 and *N*=1/1), (2 µM: *n*=16/17 and *N*=4/4), (3 µM: *n*=5/5 and *N*=1/1), for 160 min: (2 µM: *n*=11/15 and *N*=3/3), for 170 min: (1 µM: *n*=12/18 and *N*=4/5), (2 µM: *n*=28/34 and *N*=8/8), for 180 min: (2 µM: *n*=4/6 and *N*=1/1). The spread of $\phi_{e\,n\,t}$ between samples is reported in terms of the circular standard deviation ($\sqrt{-2\,\text{ln}\,R}$, where R is the first Kuramoto order parameter). Colors mark concentration of DAPT. Drug pulse duration was kept constant at 30 min/cycle. (**B**) Stroboscopic maps for different values of *zeitgeber* period and *zeitgeber* strength placed next to each other. The localized region close to the diagonal in each map marks $\phi_{e\,n\,t}$ for that condition. This is highlighted with a magenta star, which corresponds to the centroid of the said region. The centroid $\left(x_{c},y_{c}\right)$ was calculated from the vectorial average of the phases of phase-locked samples at the end of the experiment, where *x*_*c*_ = vectorial average of old phase, *y*_*c*_ = vectorial average of new phase. The spread of the points in the region is reported in terms of the circular standard deviation ($\sqrt{-2\,\text{ln}\,R}$, where R is the first Kuramoto order parameter). The period of the DAPT pulses and the concentration of DAPT are indicated. Colors mark progression in time, from purple to yellow. NC means not considered (in plotting A) because no/small fraction of samples was phase-locked – for 0.5 µM 130 min: *n*=4/9 and *N*=2/2, for 0.5 µM 150 min: *n*=0/6 and *N*=0/1. Stroboscopic maps for these NC conditions include all samples, unlike the other conditions where only phase-locked samples are plotted. Data for 2 µM condition is the same as that in Figure 7A–B.

Such vertical shifts in PRCs as a function of entrainment period have been previously observed in so-called ‘overdrive suppression’ for cardiac cell oscillators (Kunysz et al., 1995). Shifts occur when the entrainment signal impacts the biochemical control of the system, leading to a change of intrinsic period (here $T_{o\,s\,c}$). Here, such change of intrinsic period can not be directly measured experimentally, as the system is entrained to another period, however, we found several lines of experimental and theoretical evidence, in addition to the vertical PRC shift, in full agreement with such an effect.

First, the slope of the $\phi_{e\,n\,t}\,\left(T_{z\,e\,i\,t}\right)$ curve in Figure 7B is unusual: PRC theory predicts that for high and low detuning this curve should have vertical slopes. Here, we observed plateaus in the data, which can be simply explained if the intrinsic period changes (see mathematical explanation in *Appendix 1*). In addition, we performed entrainment release experiments, where the segmentation clock is first entrained, then released (Figure 7—figure supplement 1): we observed a slow recovery over several cycles to a period matching control samples, compatible with a transient change of the intrinsic period. This effect was confirmed by a more precise study of the phase return map after release (Figure 8—figure supplement 3A). Combined, the inference of the PRC based on entrainment quantification data thus reveals two properties: a highly asymmetrical, mainly negative PRC and second, a change of the intrinsic period during entrainment.

### ERICA model and Arnold tongue

To gain understanding of these findings from a theory perspective, we next build a minimal model of our data.

First, we take the PRC computed at $T_{z\,e\,i\,t}=140$ min to be the ‘reference’ curve, since this $T_{z\,e\,i\,t}$ coincides with the natural period of the process. We can then estimate for each entrainment period the period shift most consistent with the data, to obtain a single, period-independent PRC in Figure 8A2 (see *Appendix 1, section Theoretical methods*). From there, we build upon the simplest non-linear phase-oscillator, that is, the classical Radial Isochron Cycle (RIC). To modulate its sine-form PRC, which is incompatible with our data, we perturb it into an Elliptic Radial Isochron Cycle with Acceleration or ERICA.

ERICA is designed to have radial isochrons, meaning that the phase (and the PRC) can be analytically computed in the entire plane. ERICA then allows for the speeding up of the cycle for some angle range (parameter $s_{*}$) or for changes of the limit cycle shape into an ellipse of increasing eccentricity (parameter λ). Both high values of λ and $s_{*}$ generate high negative asymmetry in the PRC (see *Appendix 1, section Theoretical methods*; the full justification of the ERICA construction, the study of its multiple properties and its connection to biological oscillators will be described elsewhere). We then used Monte Carlo (MC) optimization to find parameters best fitting the experimental PRC. The results from this MC optimization put the oscillator far from the standard RIC oscillator (see cycle and corresponding flow in Figure 8B), consistent with strongly negative PRCs, with a moderate value of perturbation ϵ=0.4, high value of λ=0.5 (indicating a strong elliptical shape), and high value of $s_{*}=5.6$ over half a cycle (indicative of a relaxation type dynamics in x with a slow decay phase followed by a fast reset). Figure 8C compares the PRC of this optimized model with the multiple data points, showing excellent agreement. In addition, we combined the ERICA framework with a simple fit for intrinsic period change (Figure 8D) to account for the experimental phase transition curves, as illustrated in Figure 8G (see also Figure 8—figure supplement 2).

With the ERICA model at hand, we derived numerically the Arnold tongues of the system and the phase/detuning curves for all entrainment parameters (Figure 8E–F). We notice that the Arnold tongues are heavily skewed toward the right, meaning that the system can be more easily slowed down than sped up, consistent with the negative PRC shape. Remarkably, while we build the model using only one entrainment drug concentration, we can also largely explain data obtained at other concentrations (Figure 8F). In particular, the Arnold tongues/our model predict a specific change of the entrainment phase as the entrainment strength is varied (Figure 8—figure supplement 3G). The comparison to experimental data (Figure 8—figure supplement 4) shows that this prediction is, qualitatively, verified. More generally, having the PRC and a minimal, coarse-grained model that captures the essential dynamical features of the segmentation clock during entrainment, including the change in intrinsic period, enables predictable control over the pace and the rhythm of the segmentation clock using the entrainment strategy.

## Discussion

In this work, we used a coarse-graining, entrainment approach to gain new insights into the properties of the segmentation clock from dynamical systems’ perspective. We mode-lock the segmentation clock to various entrainment periods and use the information about the dynamic phase-locking behavior to derive the somite segmentation clock PRC from the experimental data.

### A coarse-graining approach captures essential dynamical features using a simple ‘one oscillator’ phase description

Given the complexity underlying the somite segmentation clock, comprised of several, interconnected, signaling pathways with countless molecular interactions, it was a priori unclear whether a simple ‘one oscillator’ phase description and a perturbation approach would capture the essential dynamical characteristics at the systems level. Another potential difficulty arises from the fact that cellular oscillators define a phase gradient, controlling the segmentation pattern (Lauschke et al., 2013). For all those reasons, one could imagine that no single phase could describe the system’s behavior, a general concern for systems of interacting oscillators already mentioned by Winfree, 1967.

Remarkably, one key finding of this work is that indeed, using a systems-level single oscillator phase description, we observe a consistent entrainment behavior, that is, period-locking with convergence toward a well-defined entrainment phase, as predicted from the oscillator phase response theory. It is important to point out that theoretical predictions are non-trivial and quantitative, such as the higher order 2:1 entrainment (Figure 5) and the dependence of the entrainment phase on detuning (Figure 7A–B). Given these experimental findings we conclude that the coarse-graining approach and the description of the entire system using a single oscillator phase is justified and enables to extract the essential dynamical properties, which are captured by a mathematical model including the system’s PRC.

### Asymmetrical segmentation clock PRC compatible with SNIC bifurcation

Insight into the system’s PRC allows to infer about the nature and characteristics of the oscillatory system, independent of its specific molecular realization (Izhikevich, 2006). For instance, excitable systems can be functionally categorized with their PRC, that is, in neuroscience, Class I excitable systems have PRCs with constant sign (Izhikevich, 2006). The canonical example of (Class I) oscillators with constant-sign PRCs is the quadratic fire and integrate model (Izhikevich, 2006), which can be used to model neuronal spiking.

In contrast, Class II excitable systems exhibit sinusoidal PRCs, examples include many biological systems such as the circadian clock (Granada et al., 2009).

This difference in PRC reflects fundamental properties of the underlying dynamics, including the associated bifurcations: Class I systems are associated with infinite period bifurcations, a typical example being the saddle-node on invariant cycle (SNIC) bifurcation. For such bifurcations, the period can be tuned and can become arbitrarily long depending on the proximity to the bifurcation, and oscillations present relaxation-type dynamics (i.e. combination of slow dynamics with fast resetting). The simplest example, the quadratic fire and integrate model, is a one variable x oscillator, where x goes from -∞ to +∞ in a finite time before resetting to -∞ (Izhikevich, 2006). The PRC for this oscillator is ‘naturally’ of constant sign because there is only one variable, which can only advance (or delay) in response to a constant signal. On the other hand, Class II systems are associated with Hopf bifurcations (Izhikevich, 2006), have a fixed period, and more sinusoidal dynamics close to the bifurcation.

Remarkably, the segmentation clock PRC that we computed here shares features of systems close to an infinite period bifurcation, that is, a highly asymmetrical, constant-sign PRC with an extended flat region. Consistent with this, the analytical ERICA model that we developed to capture the experimental PRCs in a wide range of conditions exhibits relaxation-type dynamics (Figure 8B, slow phase in white and fast resetting phase in gray). It is nevertheless important to point out that a given PRC can result from different oscillators, that is, a constant sign does not prove the existence of a SNIC oscillator. For instance, delayed oscillators close to a Hopf bifurcation can show PRCs with an (almost) constant sign (Kotani et al., 2012). Infinite period can also be obtained with saddle homoclinic bifurcations (Izhikevich, 2006). However, such models are mathematically more complex, for instance delays include long-term memory in the system, and homoclinic bifurcations require the existence of other attractors outside of the cycle.

In agreement with SNIC oscillators, the segmentation clock does show a tunable period and a slowing down behavior at multiple levels: it is known that the overall rate of segmentation slows down over developmental time, a feature described in several species. In addition, the oscillations also slow down along the embryonic axis, that is, a period gradient is present within the PSM, reflecting tunability of oscillations at cellular level, as PSM cells progress toward differentiation and eventually stop oscillations. How the tunability at cellular level is linked to the system-level change in segmentation clock rate is not understood (see also below for macro- versus micro-scale comparison). However, our findings of mostly negative PRCs might reflect that the segmentation clock has a natural bias of slowing down the oscillations.

From a network standpoint, biological networks with tunable periods have been associated with specific structural features, combining negative and positive feedback loops (Tsai et al., 2008). Positive feedback in particular puts oscillators close to a multistable regime, leading to relaxation oscillations (Ferrell et al., 2011). SNICs appear naturally when the regulatory logic of a system moves from a negative feedback oscillator to multistability (Jutras-Dubé et al., 2020), reflecting network modularity. Such modularity and robustness with changes in regulatory logic is a hallmark of developmental plasticity (West-Eberhard, 2003) and hence one would anticipate to find SNIC oscillators to be abundant in developing systems. First examples are indeed emerging, such as during *Caenorhabditis elegans* development (Meeuse et al., 2020).

### Findings not predicted by the theory of PRC

We also made several unexpected findings, not predicted by the theory of PRC.

First, we found evidence that during entrainment, not only does the segmentation clock adjust its observed period to the *zeitgeber* pulses, but also changes its intrinsic period in the direction of the *zeitgeber* rhythm. Hence, during entrainment that slows down the clock (i.e. 170 min), we find evidence that the intrinsic period lengthens (not just the observed rhythm), while during entrainment that speeds up the clock (i.e. 120 min), we find evidence that the intrinsic period decreased. Again, such a change of intrinsic period is not predicted in entrained systems. The simplest explanation could be that the drug pulses change the period of the oscillator by changing some biochemical parameter in the system, similar to overdrive suppression in cardiac cells (Kunysz et al., 1995). However, as stated above, we do not find evidence for a consistent slowing down or speeding up effect: the intrinsic period is decreased for short entrainment cycles and increased for longer entrainment cycles. This rather suggests the existence of feedbacks of the clock on itself, leading to higher-order adaptations beyond the rapid Notch phase response. One can only speculate on the mechanisms underlying such adaptation, but this is compatible with the idea that two interacting oscillators control the intrinsic period. Here, it is possible that entraining Notch with a *zeitgeber* modifies the Wnt oscillation period, which in turn feeds back on Notch on a longer time scale. So the internal period change that we see might in fact come from the induced change of Wnt period. We have shown previously that Wnt and Notch oscillators are coupled but are not phase-locked, with functional impact on tissue patterning (Sonnen et al., 2018). Alternatively, a similar role could also be played by the long inter- or intracellular delays in the system, postulated in multiple theoretical works (Lewis, 2003; Morelli et al., 2009; Ares et al., 2012). Such delays could effectively couple multiple cycles, changing clock parameters (such as the period) beyond the instantaneous phase response. More experimental and theoretical work is needed to explore these ideas.

Second, a striking outcome we obtained was that even after entrainment, a spatial period gradient and phase waves emerged over time (Appendix 1—figure 1). Put differently, while the overall system is entrained, as evidenced by a control of the timing of morphological segmentation and of segmentation clock rhythm, the underlying cellular oscillators show a divergent, yet, coherent response, that is, a spatial period gradient emerges within the tissue. A recent entrainment model of coupled PSM oscillators (Juul et al., 2018) does not account for the emergence of a period gradient during entrainment. This unexpected behavior hence awaits further experimental and theoretical analysis. In particular, the role of intra- and intercellular coupling needs to be addressed to reveal how the macro-scale behavior relates to the underlying cellular scale oscillations.

### Conclusions

Our work demonstrates how, despite all the molecular and functional complexities, coarse-graining and theory can be used to effectively take control of complex biological processes. A *molecular* mechanism is not needed to exert control, as long as we have a *mathematical* one, one that captures the essential features of a system at a meaningful coarse-grained level.

We also aim to illustrate the potential of an integrated, theoretical-experimental approach to complex biological systems: while from theoretical viewpoint, the fact that entrainment phase varies as detuning is altered is a mathematical necessity and hence ‘obvious’, this outcome is not at all intuitive, a priori, from an experimental-observational viewpoint. Theory guides experimentation, that is, Figure 1 Theory (Phillips, 2015), leading to new insight and understanding of a complex biological phenomenon.

Future studies are needed to gain further insight into the response to other perturbation regimes. In addition, it will be essential to perform the analysis both at the macro-scale, as done in this study, but also extending it to the cellular scale, that is, taking spatial differences, such as the period gradient, fully into account. We consider that such a dynamical systems’ theoretical-experimental approach is a promising and, as we show here, feasible way forward with the goal in categorizing the segmentation clock in its universality class.

## Materials and methods

Please refer to the *Appendix 1* for detailed materials and methods.

## Figures and Tables

**Appendix 1—figure 1.**
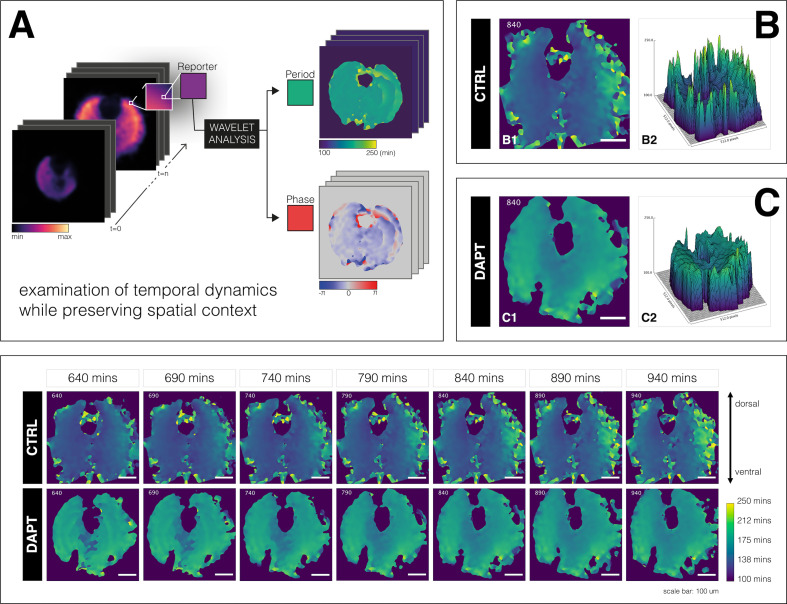
A spatial period gradient emerges in the tissue even upon entrainment of the segmentation clock. Left: Schematic of pipeline to generate period and phase movies using pixel-by-pixel wavelet analysis (**A**). Illustration by Stefano Vianello. Right: Snapshot of period wavelet movie of control subjected to 170 min periodic pulses of DMSO (**B**) or entrained sample subjected to 170 min periodic pulses of 2 µM DAPT (**C**), taken at 840 min after the start of experiment. Period is either shown using heatmap (**B1,C1**) or as a surface plot (**B2,C2**). Bottom: Snapshots of period wavelet movie of samples in (**B**) and (**C**) at different time points. Time is indicated as minutes elapsed from the start of the experiment. Sample is rotated so that the dorsal side is up. Timelapse of period wavelet movies and corresponding surface plots are available at https://youtu.be/3Y53OhXacKI.

Appendix 1—figure 1—video 1. Period gradient of LuVeLu in E10.5 2D-assay subjected to 170 min periodic pulses of 2 µM DAPT (or DMSO for control) available at https://youtu.be/3Y53OhXacKI.

Appendix 1—figure 1—video 2. E10.5 2D-assay, expressing LuVeLu, subjected to 170 min periodic pulses of DMSO control, with corresponding period and phase wavelet movies, available at https://github.com/PGLSanchez/EMBL-files/blob/master/MOVIES/SO_2.0D_170mins_CTRL.avi.

Appendix 1—figure 1—video 3. E10.5 2D-assay, expressing LuVeLu, subjected to 170 min periodic pulses of 2 µM DAPT, with corresponding period and phase wavelet movies, available at https://github.com/PGLSanchez/EMBL-files/blob/master/MOVIES/SO_2.0D_170mins_DAPT.avi.

Appendix 1—figure 1—video 4. E10.5 2D-assay, expressing LuVeLu, subjected to 130 min periodic pulses of DMSO control, with corresponding period and phase wavelet movies, available at https://github.com/PGLSanchez/EMBL-files/blob/master/MOVIES/SO_2.0D_130mins_CTRL.avi.

Appendix 1—figure 1—video 5. E10.5 2D-assay, expressing LuVeLu, subjected to 130 min periodic pulses of 2 µM DAPT, with corresponding period and phase wavelet movies, available at https://github.com/PGLSanchez/EMBL-files/blob/master/MOVIES/SO_2.0D_130mins_DAPT.avi.

**Appendix 1—figure 2.**
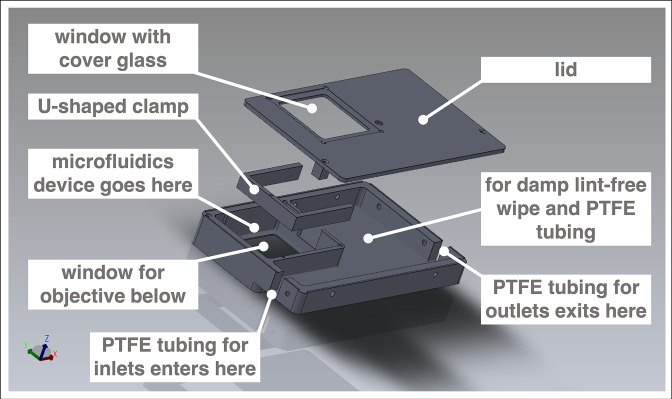
Customized box to mount microfluidics device on microscope for simultaneous culture, entrainment, and live imaging. Computer-aided design (CAD) of metal box customized to hold a microfluidics device bonded to a 70 mm × 70 mm cover glass. The box fits the stage of an LSM 780 laser-scanning microscope (Carl Zeiss Microscopy). Design by Katharina Sonnen, the EMBL Mechanical Design Office, and the EMBL Mechanical Workshop.

**Appendix 1—figure 3.**
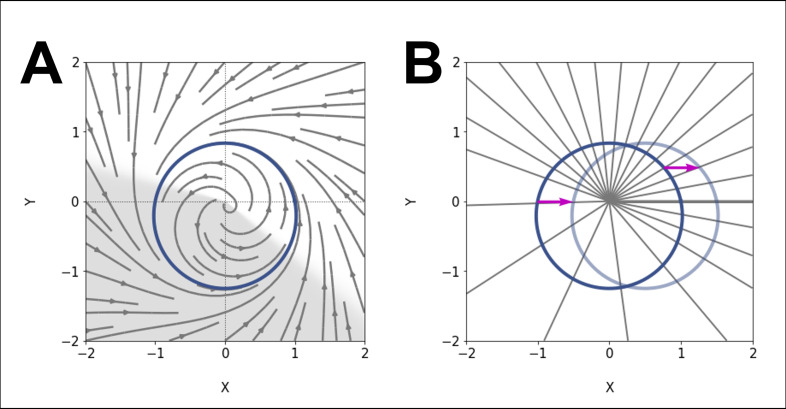
ERICA model. (**A**) Our model consists of an elliptic limit cycle (blue) with increased angular velocity in one sector (shaded region). (**B**) The effect of such acceleration is to change the spacing of isochrons (radial lines). We compute the phase response curve (PRC) of the model by introducing a perturbation (arrow) at points on the limit cycle and looking at the starting and ending isochrons.

**Appendix 1—figure 4.**
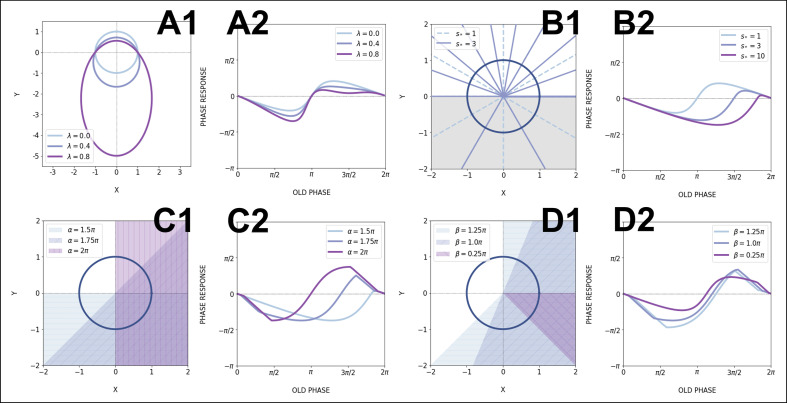
Effects of model parameters on the phase response curve (PRC). (**A**) Effect of eccentricity λ: (**A1**) Increasing λ makes the limit cycle more elongated, while keeping the upper focus at the origin. (**A2**) The positive part of the PRC is then flattened; for all PRCs here $s_{*}=1$ (no acceleration). (**B**) Effect of speeding up $s_{*}$: (**B1**) When $s_{*}$ is increased, the spacing between isochrons in the sped up region increases, so this region contains less and less isochrons. Shown here is the case when the acceleration happens in the lower half-plane (α=1.5⁢π, β=π), which is shaded in gray. (**B2**) The effect on the PRC is to shrink the portion where the oscillator is sped up, thus emphasizing the other part of the curve. Here, λ=0. (**C, D**) Effects of the sped up sector parameters α,β: the location and width of the sector determine which parts of the PRC get rescaled. (**C1**) The shaded regions are sectors with different values of α and β=π. (**C2**) The corresponding PRCs with,λ=0.$s_{*}=10$. (**D1**) Sped up sectors located at α=15/8⁢π with different widths β. (**D2**) The corresponding PRCs for the case λ=0.$s_{*}=10$. All PRCs in this figure were computed with perturbation amplitude A=0.6.

## Data Availability

Source data and Python codes are made available via GitHub (https://github.com/PGLSanchez/EMBL_OscillationsAnalysis [copy archived at swh:1:rev:bd4a258570e7b7b5f0c179bc976f0d45988efa1a] and https://github.com/PGLSanchez/EMBL-files).

## Additional files

MDAR checklist

## Appendix 1

**Appendix 1—key resources table:**

Reagent type (species) or resource	Designation	Source or reference	Identifiers	Additional information
Genetic reagent (*Mus musculus*)	LuVeLu	Aulehla et al.	DOI:10.1038/ncb1679	
Genetic reagent (*Mus musculus*)	Mesp2-GFP	Morimoto et al.	DOI:10.1016/j.ydbio.2006.08.043	
Genetic reagent (*Mus musculus*)	Axin2-linker- Achilles	This work		see Materials and methods > Mouse lines
Chemical compound, drug	Bovine serum albumin (BSA)	Equitech-Bio	BAC62	
Chemical compound, drug	DMEM/F12	Cell Culture Technologies	Customized	No glucose, glutamate, L-glutamine, and phenol red
Chemical compound, drug	1 M HEPES	Gibco	15630-106	
Chemical compound, drug	10,000 U/mL PenStrep	Gibco	15140-122	
Chemical compound, drug	45% glucose	Sigma	G8769	
Chemical compound, drug	200 mM L-glutamine	Gibco	25030-081	
Chemical compound, drug	Fibronectin	Sigma-Aldrich	F1141	
Chemical compound, drug	Cascade Blue	Invitrogen	C-3239	
Chemical compound, drug	DMSO	Sigma-Aldrich	D8418	
Chemical compound, drug	DAPT	Sigma-Aldrich	D5942-5MG	Stock concentration in DMSO: 10 mM
Commercial assay, kit	SYLGARD 184 silicone kit	Dow	1673921	9:1 (w/w) elastomer base: curing agent
Software, algorithm	Microscopy Pipeline Constructor (MyPiC)	Politi et al., 2018	DOI:10.1038/nprot.2018.040	https://github.com/manerotoni/mypic/
Software, algorithm	Fiji	Schindelin et al.	DOI:10.1038/nmeth.2019	
Software, algorithm	Matplotlib	Hunter	DOI:10.1109/MCSE.2007.55	
Software, algorithm	NumPy	van der Walt et al.	DOI:10.1109/MCSE.2011.37	
Software, algorithm	pandas	McKinney	DOI:10.25080/Majora-92bf1922-00a	
Software, algorithm	scikit-image	van der Walt et al.	DOI:10.7717/peerj.45310.7717/peerj.453	
Software, algorithm	SciPy	Virtanen et al.	DOI:10.1038/s41592-019-0686-2	
Software, algorithm	seaborn	Waskom et al.	DOI:10.5281/zenodo.12710	
Software, algorithm	PlotTwist	Goedhart	DOI:10.1371/journal.pbio.3000581	https://huygens.science.uva.nl/PlotTwist/
Software, algorithm	PlotsOfData	Postma and Goedhart	DOI:10.1371/journal.pbio.3000202	https://huygens.science.uva.nl/PlotsOfData/
Software, algorithm	PlotsOfDifferences	Goedhart	DOI:10.1101/578575	https://huygens.science.uva.nl/PlotsOfDifferences/
Software, algorithm	pyBOAT	Mönke, 2020a	DOI:10.1101/2020.04.29.067744	https://github.com/tensionhead/pyBOAT/
Software, algorithm	Spatial pyBOAT (SpyBOAT)	Mönke, 2020b		https://github.com/tensionhead/SpyBOAT/
Software, algorithm	Entrainment Analysis Jupyter notebook	This work, Sanchez, 2022		EntrainmentAnalysis in https://github.com/PGLSanchez/EMBL_OscillationsAnalysis/
Other	Cover glass	Marienfeld	0107999 098	70 mm × 70 mm, 1.5H, see Materials and methods > Preparations for microfluidics- based entrainment of 2D-assays
Other	PTFE tubing	APT	AWG24T	Inner diameter: 0.6 mm, see Materials and methods > Preparations for microfluidics- based entrainment of 2D-assays
Other	Syringe needle	BD Microlance 3	300900	22G×1.25" – Nr. 12, see Materials and methods > Preparations for microfluidics- based entrainment of 2D-assays
Other	3 mL syringe	BD Luer-Lok	309658	Diameter: 8.66 mm, see Materials and methods > Preparations for microfluidics- based entrainment of 2D-assays
Other	10 mL syringe	BD Luer-Lok	300912	Diameter: 14.5 mm, see Materials and methods > Preparations for microfluidics- based entrainment of 2D-assays
Other	Programmable pump	World Precision Instruments	AL-400	see Materials and methods > Setting up automated pumping
Other	LSM 780 laser-scanning microscope	Carl Zeiss Microscopy		see Materials and methods > Confocal microscopy

### Materials and methods

#### Mouse lines

For most of the experiments in this study, we used a transgenic mouse line expressing a dynamic Notch signaling reporter driven from the *Lfng* promoter, more commonly known as LuVeLu. The generation of LuVeLu was previously described (Aulehla et al., 2008). Briefly, the expression of Venus, an improved version of YFP (Nagai et al., 2002), was driven from a 2 kb fragment of the *Lfng* promoter (Morales et al., 2002; Cole et al., 2002). The locus was flanked by *Lfng* 3’-UTR and a modified PEST domain (Rogers et al., 1986) to destabilize the reporter mRNA and protein, respectively. For experiments visualizing Mesp2 during entrainment, we used the Mesp2-GFP mouse line generated by Morimoto et al., 2006. Axin2-linker-Achilles, a mouse line expressing Axin2 fused with a GSAGS linker to Achilles, a fast-maturing variant of YFP (Yoshioka-Kobayashi et al., 2020), was generated in-house. To generate the knock-in alleles, we targeted the stop codon of endogenous Axin2 locus with vector containing the reporter sequence coding for Achilles and a selection cassette. This targeting vector was constructed as follows: linker-Achilles-loxP-PGK Neo-loxP. The selection cassette was flanked by loxP- sites for eventual Cre-mediated excision. Axin2-linker-Achilles knock-in reporter line was generated by standard gene targeting techniques using R1 embryonic stem cells. Briefly, chimeric mice were obtained by C57BL/6 blastocyst injection and then outbred to establish the line through germline transmission. The Achilles/pRSETB plasmid was a gift from the lab of Atsushi Miyawaki at RIKEN Center for Brain Science (RIKEN-CBS) in Japan. Mice were kept in an outbred background and were housed in the EMBL Laboratory Animal Resources (LAR). All animal experiments were conducted under veterinarian supervision and after project approval by European Molecular Biology Laboratory, following the guidelines of the European Commission, Directive 2010/63/EU and AVMA Guidelines 2007.

#### Media preparation

On the day of the experiment, dissection medium and culture medium were freshly prepared as indicated in Appendix 1—table 2. Culture medium was filter sterilized using a PVDF filter (pore size: 0.22 µm, Merck). Both dissection medium and culture medium were equilibriated to 37°C for at least 15 min, and were kept in a 37°C incubator under 5% CO_2_ until use.

**Appendix 1—table 1.** Recipe for media preparation. Formulations specified here are for the preparation of approximately 50 mL of medium. Special DMEM/F12* used in these media does not contain glucose, L-glutamine, sodium pyruvate, and phenol red. Culture medium is filter sterilized after preparation.

Component	Dissection medium	Culture medium
BSA (Equitech-Bio, BAC62)	0.5 g	0.02 g
DMEM/F12* (Cell Culture Technologies)	50 mL	50 mL
1 M HEPES (Gibco, 15630-106)	1 mL	–
10,000 U/mL PenStrep (Gibco, 15140-122)	–	500 µL
45% Glucose (Sigma, G8769)	44.4 µL	44.4 µL
200 mM L-Glutamine (Gibco, 25030-081)	500 µL	500 µL

#### Mouse dissection and embryo recovery

Female mice were sacrificed on 10.5 dpc (days post coitum) via cervical dislocation. The skin on their ventral side (belly area) was wiped with 70% ethanol, and an incision was made using a clean pair of surgical scissors. The uterine horns were harvested and were washed once with dissection medium. In dissection medium, under a stereo microscope (Leica M80), the deciduae were cut open using clean forceps and the embryos were recovered. The embryos were again washed with fresh dissection medium and their tails were clipped using forceps. Clipped tails were then transferred to a new dish with fresh dissection medium. The volume of dissection medium in the dish was kept to minimum to lessen autofluorescence, which could interfere with subsequent screening. The tails were then screened for presence of the reporter-of-interest (e.g. LuVeLu) using a stereo fluorescence microscope. The tailbud was cut from the rest of the tail and was immediately transferred to pre-equilibriated culture medium that was supplemented with HEPES (170 µL of 1 M HEPES in 10 mL culture medium). These isolated embryonic tissues were then directly loaded in the microfluidics device.

#### Preparations for microfluidics-based entrainment of 2D-assays

We used in this study a microfluidics-based experimental entrainment platform with a general protocol elaborated in a recent publication (van Oostrom et al., 2021). The PDMS microfluidics device was made using standard soft lithography (El Debs et al., 2012) and as previously described (Sonnen et al., 2018). The ratio of Sylgard 184 silicone elastomer base (Dow) to curing agent (Dow) was 9:1 (w/w). PDMS chip was attached to cover glass (70 mm × 70 mm, 1.5H, Marienfeld 0107999 098) via plasma bonding. UV irradiation of PTFE tubing and PDMS device PTFE tubing (inner diameter: 0.6 mm, APT AWG24T) and syringe needles (22 G 1 1/4 – Nr. 12), as summarized in Appendix 1—table 3, were prepared a day prior to actual microfluidics-based entrainment experiment. In addition to one PDMS microfluidics device (Figure 1—figure supplement 1), each experiment required four 3 m PTFE tubing (each with syringe needle inserted in one end) for the drug/medium inlets, two 1 m PTFE tubing (each with syringe needle inserted in one end) for the outlets, and twenty-four 1 cm plugs made from cut PDMS-filled PTFE tubing for the sample inlets and unused drug/medium inlets. These were all sterilized under UV for at least 20 min.

**Appendix 1—table 2.** List of PTFE tubing needed for a microfluidics-based entrainment experiment. For the first two items, a syringe needle is to be inserted inside one end of each tubing. Plugs are made from cut PDMS-filled PTFE tubing, and are used to seal inlets after sample loading. Controls are already taken into account in the specified quantities.

Item	With needle?	Quantity	Use
3 m PTFE tubing	Y	4	Drug/medium inlet
1 m PTFE tubing	Y	2	Outlet
1 cm plug	N	24	Sample inlet+unused drug/medium inlet

##### Fibronectin-coating of PDMS device and overnight soaking in buffer

While waiting, 5 mL of PenStrep (Gibco, 15140-122) was added to 495 mL of ×1 PBS (PBS + PenStrep); 5.6 mL of the buffer was set aside to prepare fibronectin solution, while the rest was poured in a glass dish. After UV irradiation, the sterilized PDMS device was immersed in the PBS + PenStrep and bubbles were removed by flushing the channels with buffer. PDMS-filled PTFE plugs were also immersed in PBS + PenStrep. To prepare the fibronectin solution, 280 µL of fibronectin (Sigma-Aldrich, F1141) was added to the set aside 5.6 mL PBS + PenStrep. At least 2.5 mL of fibronectin solution was loaded into a 3 mL syringe (diameter: 8.66 mm, BD Luer-Lok REF 309658). A UV-irradiated needle, which was earlier inserted into a 1-meter PTFE tubing, was attached to the filled syringe. The tubing was then inserted into an outlet in the PDMS device, carefully avoiding introduction of bubbles. Fibronectin was flowed (flow rate: 50 µL/hr) into the PDMS device at room temperature overnight.

##### Preparation of syringes containing drug/medium

For a microfluidics-based experiment with periodic pulses of drug, four 10 mL syringes (diameter: 14.5 mm, BD Luer-Lok REF 300912) were filled with either the drug, DMSO control, or culture medium (see Appendix 1—table 2). Components of solution in each of these syringes are specified in Appendix 1—table 4. Drug used in this study was DAPT (Sigma-Aldrich, D5942-5MG). To prepare 10 mM stock of DAPT, 5 mg DAPT (MW = 432.46 g/mol) was dissolved in 1156.2 µL DMSO (Sigma-Aldrich, D8418). This was aliquoted and stored at –20°C until use.

**Appendix 1—table 3.** List of syringe containing drug/medium for a microfluidics-based entrainment experiment. Formulations specified here are for any drug with final concentration *X* µM. For recipe to prepare culture medium, please refer to Appendix 1—table 2.

Component	Syringe A	Syringe B	Syringe C	Syringe D
	DRUG	CONTROL	MEDIUM	MEDIUM
Drug (10 mM in DMSO)	*X* µL	–	–	–
DMSO (Sigma-Aldrich, D8418)	–	*X* µL	–	–
Cascade Blue (Invitrogen, C-3239)	2 µL	2 µL	–	–
Culture medium (see Appendix 1—table 2)	Dilute to 10 mL	Dilute to 10 mL	10 mL	10 mL

##### Degassing drug/medium and PDMS device

After coating and overnight soaking, the PTFE tubing was cut away from the needle and was immediately immersed in the buffer. The dish containing immersed PDMS device, with attached tubing for the two outlets, and plugs were placed inside a vacuum desiccator chamber. The plunger of each syringe containing the drug/medium was pulled to maximum. The syringes were then also placed in the vacuum chamber, almost vertically, with the plunger resting on the desiccator. These were degassed under vacuum for at least 1.5 hr.

#### Loading embryonic tissues in microfluidics device and mounting for live imaging

Before mouse dissection and recovery of mouse embryos, syringes containing the degassed drug/medium were each connected to a UV-irradiated 3 m PTFE tubing via attached syringe needle, and were carefully mounted on programmable pumps (World Precision Instruments, AL-400) next to the microscope. Gas in the tubing was displaced with drug/medium by careful pushing of the syringes’ plunger. Pumps were turned on and flow rate was set to 900 μL/hr. The microscope was then equilibriated to 37°C and 5% CO_2_. After recovery of embryonic tissues, using a pipette (i.e. P200 for 2D-assays and P1000 for intact PSM), each sample was carefully loaded into the microfluidics device, which was already coated with fibronectin, immersed in a buffer of PBS and PenStrep, and degassed. Each sample inlet was plugged with a PDMS-filled PTFE tubing immediately after sample loading. Unused inlets, if any, were also plugged. Flow rate of drug/medium was set to 20 μL/hr and the tubings were carefully inserted into the drug/medium inlets in the PDMS device while it is immersed in buffer. The tubings connected to the syringes with medium were inserted first, and the tubing connected to the syringe with the drug was inserted last. A 15 min timer was started after insertion of the drug tubing. PDMS device was then removed from the buffer and excess liquid on the cover glass was removed carefully with lint-free wipes (Kimberly-Clark). The PDMS device, with more than 1 m of attached tubings, was carefully placed inside a pre-equilibriated microfluidics holder (EMBL Mechanical Workshop, Appendix 1—figure 2) customized to fit the 70 mm × 70 mm cover glass (Marienfeld 0107999 098) and some PTFE tubing. Putting some of the tubing inside the microfluidics holder was necessary to equilibriate the drug/medium to desired environmental conditions before they are perfused into the microfluidics device. The cover glass was secured in place with grease and a U-shaped metal clamp. The microfluidics holder was then carefully mounted on the stage of the microscope, and the end of the outlet tubings were placed in a beaker. Fifteen minutes after insertion of the drug tubing in the PDMS device, each pump was tilted on its side and was equilibriated for another 15 min. Afterward, the pump mounting the syringes with the drug and DMSO control was turned off, and the pump mounting the syringes with the medium was set to flow rate of 60 µL/hr. Samples were equilibrated at these conditions for at least 30 min before start of imaging/entrainment.

#### Setting up automated pumping

Entrainment via periodic pulses of drug was performed through alternate perfusion of medium and drug into the microfluidics device. Perfusion of drug/medium was done using syringes mounted on programmable syringe pumps (World Precision Instruments, AL-400). Diameter of syringe (14.5 mm for 10 mL syringe, BD Luer-Lok REF 300912) was accordingly set to match a defined flow rate and a specified volume of solution to be perfused with the duration of perfusion. Standard pumping programs of medium and drug/control are summarized in Appendix 1—table 5, respectively, considering an entrainment experiment with $T_{z\,e\,i\,t}$ = 170 min.

#### Confocal microscopy

For most of the experiments here, samples were imaged in an LSM 780 laser-scanning microscope (Carl Zeiss Microscopy) fitted with an incubation chamber (EMBL Mechanical Workshop). A Plan-Apochromat ×20 air objective with a numerical aperture (NA) of 0.8 (Carl Zeiss Microscopy) was used for imaging, and the zoom was set to 0.6. Three z-stacks (spacing: 8 µm) were scanned for each sample every 10 min to acquire timelapse movies. Imaging of multiple samples in multiple locations was done with a motorized stage, controlled using Zen Black software (Carl Zeiss Microscopy), and automated using a VBA macro developed by Antonio Politi (Politi et al., 2018), which is available at https://github.com/manerotoni/mypic. The dimension of the images was 512 pixels × 512 pixels, with a pixel size of 1.38 µm and bit depth of 16 bit. Detection of drug pulses, using Cascade Blue (excited with 405 nm) added to the solution, was also done every 10 min with lower image resolution: 1 z-stack, 32 pixels × 32 pixels (pixel size = 22.14 µm). Imaging and automated pumping of drug/medium through the microfluidics device were started simultaneously.

**Appendix 1—table 4.** Pumping program of medium for entrainment to 170 min periodic pulses of drug.

Phase	Function	Rate	Volume	Remark
PH:01	LP:ST			
PH:02	RATE	60 µL/hr	140 µL	Perfuse medium for 140 min
PH:03	LP:ST			
PH:04	PS:60			Pause for 60 s
PH:05	LP:30			Loop PH:04 30 times (pause for 30 min=60 s × 30)
PH:06	LP:30			Loop PH:02 to PH:05 30 times (total: 30 periodic pulses)
PH:07	STOP			

**Appendix 1—table 5.** Pumping program of drug/control for entrainment to 170 min periodic pulses of drug.

Phase	Function	Rate	Volume	Remark
PH:01	LP:ST			
PH:02	LP:ST			
PH:03	LP:ST			
PH:04	PS:60			Pause for 60 s
PH:05	LP:70			Loop PH:04 70 times (pause for 70 min=60 s × 70)
PH:06	LP:02			Loop PH:04 to PH:05 2 times (pause 140 min=70 mins × 2)
PH:07	RATE	60 µL/hr	30 µL	Perfuse drug/control for 30 min
PH:08	LP:30			Loop PH:02 to PH:07 30 times (total: 30 periodic pulses)
PH:09	STOP			

#### Data analysis

Details on the coarse-graining strategy and analyses of entrainment dynamics are specified here.

##### Extracting timeseries from global intensity of 2D-assays for subsequent analyses

To extract the timeseries corresponding to the segmentation clock (Figure 2, Figure 1—figure supplement 1A), global intensity analysis of timelapse fluorescence imaging was done using Fiji (Schindelin et al., 2012). Z-stacks were first projected based on maximum intensity (in Fiji: Image > Stacks > Z Project). Subsequently, the timeseries was obtained by plotting the z-axis profile (in Fiji: Image > Stacks > Plot Z-axis Profile). Timeseries of replicate samples were compiled in a .txt file for subsequent analyses. To extract timeseries corresponding to signaling oscillations localized at the center of 2D-assays, samples that were centered in the field of view were considered. A 50 pixel × 50 pixel oval ROI was specified at the center (*x*=256 and *y*=256) of a re-oriented (and registered, if necessary) 2D-assay timelapse file (in Fiji: Edit > Selection > Specify). Timeseries was extracted after specifying the center ROI (in Fiji: Image > Stacks > Plot Z-axis Profile). Timeseries of replicate samples were compiled in a .txt file for subsequent analyses. Definition of Kuramoto order parameter We follow the definition from Pikovsky et al., 2001: introducing the mean field variable

(4) $$Z=\frac{1}{N}\,\sum_{k=1}^{N}e^{i\,\phi_{k}}=R\,e^{i\,\Theta }$$

(see also Riedel-Kruse et al., 2007), the first Kuramoto order parameter is defined by

(5) R=|Z|

##### Monitoring period-locking and phase-locking

Entrainment was evaluated based on period-locking and phase-locking of the signaling oscillations to the periodic drug pulses. Oscillatory components were extracted from timeseries using a wavelet analysis workflow that was developed by Gregor Mönke, which was recently implemented as a Python-based standalone software (Mönke et al., 2020c, available at https://github.com/tensionhead/pyBOAT; Mönke, 2020a). In this workflow, timeseries was first detrended using a sinc filter and then subjected to continuous wavelet transform. The *Morlet wavelet* is used as the mother wavelet (Mönke et al., 2020c). Time-resolved frequency analysis was done by cross-correlating the signal to wavelet functions of known frequencies, generating a power spectrum. A high power score was assigned to wavelets that correlated well with the signal relative to white noise. Instantaneous period and phase were extracted upon evaluation of the power spectrum along a ridge tracing wavelet with maximum power for every timepoint. Phase dynamics of signaling oscillations, upon subjecting them to periodic perturbation, were analyzed using stroboscopic maps (Balanov, 2008; Cross and Siggia, 2005; Isomura et al., 2017). Briefly, the phase difference (Δ⁢ϕ) was defined as

$$\Delta \,\phi =\phi \,\left(t+T_{z\,e\,i\,t}\right)-\phi \,\left(t\right)$$

where t is the time of perturbation and $T_{z\,e\,i\,t}$ is the *zeitgeber* period (i.e. one cycle after time = t). ϕ⁢(t) and $\phi \,\left(t+T_{z\,e\,i\,t}\right)$ denote the old_phases and their corresponding new_phases, respectively. The stroboscopic maps were then plotted as new_phases versus old_phases (for scheme, please refer to Figure 6A). The centroid, marking the entrainment phase ($\phi_{e\,n\,t}$), was determined considering only phase-locked samples (where the difference between a sample’s phase at the time of the final drug pulse considered and its phase one drug pulse before is less than π/8). This was quantified from the average phases of the final (old_phase,new_phase) pairs considered, and the circular standard deviation (circSD) was calculated using the formula:

$$c\,i\,r\,c\,S\,D=\sqrt{-2\,\ln R}$$

where R is the first Kuramoto order parameter. As wavelets only partially overlap the signal at the edges of the timeseries, resulting in deviations from true phase values (Mönke et al., 2020c), the first and last pulse pairs were not considered in the generation of stroboscopic maps. Polar plots were also generated summarizing the instantaneous phase of replicate samples and their first Kuramoto order parameter as shown in Figure 4D, Figure 5D, and Figure 4—figure supplement 1B. The Python code is available as a Jupyter notebook (.ipynb) at https://github.com/PGLSanchez/EMBL_OscillationsAnalysis/tree/master/EntrainmentAnalysis (Sanchez, 2022; copy archived at swh:1:rev:bd4a258570e7b7b5f0c179bc976f0d45988efa1a). This code uses Matplotlib (Hunter, 2007), NumPy (van der Walt et al., 2011; Harris et al., 2020), pandas (McKinney, 2010), scikit-image (van der Walt et al., 2014), SciPy (Virtanen et al., 2020), and seaborn (Waskom et al., 2014).

Detrended timeseries of replicate samples were in some part of the study represented as a heatmap using PlotTwist (Goedhart, 2020), as shown in Figure 4A. Average periods (from 650 to 850 min after start of experiment) were meanwhile plotted using PlotsOfData (Postma and Goedhart, 2019), as shown in Figure 3C–D and Figure 3—figure supplement 1. These apps are available at https://huygens.science.uva.nl/PlotTwist/ and at https://huygens.science.uva.nl/PlotsOfData/, respectively. To calculate p-values, two-tailed test for absolute difference between medians was done via a randomization method using PlotsOfDifferences (Goedhart, 2019). While not recommended, p-values were also calculated similarly for conditions with *n*<10. PlotsOfDifferences is available at https://huygens.science.uva.nl/PlotsOfDifferences/.

### Generating wavelet movies

Period and phase wavelet movies were generated using the Wavelet Processing and Export workflow developed by Gregor Mönke, which runs on the EMBL cluster and is implemented in Galaxy with technical assistance from Jelle Scholtalbers (EMBL Genome Biology Computational Support). This workflow extracts timeseries of every pixel in a timelapse movie and subjects them to sinc filter-based detrending and subsequent continuous wavelet transform (Mönke et al., 2020c). This results in extraction of instantaneous period and phase of each pixel, recovering period and phase wavelet movies corresponding to the input timelapse movie (for scheme, please refer to Appendix 1—figure 1A). The workflow is available at https://github.com/tensionhead/SpyBOAT, (Mönke, 2020b).and can be used via a public Galaxy server at https://usegalaxy.eu/root?tool_id=/spyboat. Settings used to generate wavelet movies in this study were: sigma of 8.0, sample interval of 10 min, period range from 100 to 250 min, and number of periods analyzed of 151.

### Examining period gradient in 2D-assays

Re-oriented 2D-assays (dorsal side up) and their corresponding period and phase wavelet movies were used for the analyses. Temporal evolution of the period gradient during the course of entrainment experiments was evaluated from the period wavelet movies of the 2D-assays. As the period wavelet movies also contained wavelet transformations for pixels in the background, a binary mask was first created to differentiate pixels corresponding to signal. To create the mask, re-oriented (and registered, if necessary) timelapse movies of 2D-assays were blurred using a Gaussian blur (in Fiji: Process > Filters > Gaussian Blur [sigma radius: 8, scaled units in µm]). Then, signal was specified by thresholding (in Fiji: Image > Adjust > Threshold [Default method, dark bakground]). After thresholding, pixels corresponding to the signal were assigned a value of 255, while those corresponding to the background were assigned a value of 0. If opposite, the values were inverted (in Fiji: Edit > Invert). Then, the binary mask (signal = 1 and background = 0) was created by dividing all values by 255 (in Fiji: Process > Math > Divide [value: 255]), and was used to mask the period wavelet movie.

### Theoretical methods

#### PRC correction and computation

PRCs at different entrainment periods appear shifted with respect to one another. To correct for this, we fitted the PRCs at entrainment periods $T_{z\,e\,i\,t}=\left(120,130,140,170\right)$ min (those for which we have enough experimental data points to build a continuous curve) using three Fourier modes (Figure 8A1) and manually chose the phase shift optimizing their overlap for Figure 8A2. The shift values used were (-0.45,-0.25,0,0.45) for $T_{z\,e\,i\,t}=\left(120,130,140,170\right)$ min, respectively. The data points shown in Figure 8C are used for MC optimization (see next section). Our choice was later validated by recomputation of PRCs and comparison with data in Figure 8G. We also show the corresponding PTC and stroboscopic maps in Figure 8—figure supplement 3D-E.

#### Model

Our model (Appendix 1—figure 3A) is based on simple modifications of the classical Poincaré oscillator, otherwise called RIC model. The main motivation is to find the simplest possible model able to reproduce the general shape of the experimental PRC, with both a flat and a negative region, while being analytical with simple isochrons. The full description and analytical calculations for this model will be described elsewhere, here we outline the basic equations and some general properties of the model. For PRC computations, we will assume that a perturbation is a horizontal shift of magnitude ϵ (Appendix 1—figure 3B).

To flatten the PRC for half the cycle, we first modify the limit cycle of the standard RIC model into an ellipse, keeping the origin as one of the foci. This is done through the introduction of a parameter λ, so that the corresponding equation of the ellipse is

(6) $$r_{c\,y\,c\,l\,e}=\frac{1}{1+\lambda \,\sin\theta }$$

combined with radial equidistant isochrons, $\dot{\theta }=1$, where θ is the polar angle. This is however not enough to define the entire flow in the plane since one needs to specify how r converges on this cycle. We thus impose a radially uniform convergence rate equal to 1 for the radius, which leads to the following differential equation in polar coordinates:

(7) $$\dot{r}={\dot{r}}_{c\,y\,c\,l\,e}+r\,\left(r_{c\,y\,c\,l\,e}-r\right)$$

where ${\dot{r}}_{c\,y\,c\,l\,e}=-\lambda \,\frac{r\,x}{{\left(r+\lambda \,y\right)}^{2}}$ (again assuming $\dot{\theta }=1$). This leads to the following equations in cartesian coordinates:

(8) $$\dot{x}=\left(\frac{r}{r+\lambda \,y}-r-\frac{\lambda \,x}{{\left(r+\lambda \,y\right)}^{2}}\right).x-y=d\,x_{\lambda }\,\left(x,y\right)$$

$\dot{y}=x+\left(\frac{r}{r+\lambda \,y}-r-\frac{\lambda \,x}{{\left(r+\lambda \,y\right)}^{2}}\right).y=d\,y_{\lambda }\,\left(x,y\right)$(9)

We name this model Elliptic Radial Isochron Cycle, or ERIC. An important feature of ERIC is that the angle in the plane is the phase of the oscillator, in particular since the phase is defined by the planar angle, the PRC following a horizontal perturbation of size ϵ toward the right can be computed in a straightforward way and is:

(10) $$P\,R\,C_{\epsilon ,\lambda }\,\left(\theta \right)=\cot^{-1}\left(\epsilon \,\left(\csc\left(\theta \right)+\lambda \right)+\cot\left(\theta \right)\right)-\theta$$

As said above, the effect of introducing λ is to smoothly flatten the PRC over half of the cycle, as illustrated in Appendix 1—figure 4A.

We then introduce a second modification, allowing us to rescale the portion of the cycle where the PRC is flattened. We modify the equations by introducing a ‘speeding factor’ s so that

(11) $$\dot{\theta }=s\,\left(\theta \right)$$

This will keep isochrons radial but will change their spacing. With the elliptic limit cycle, this leads to the differential equation in cartesian coordinates

(12) $$\begin{array}{cc} \dot{x}=s\,\left(\theta \right)\,d\,x_{\lambda }\,\left(x,y\right) & \end{array}$$

(13) $$\begin{array}{cc} \dot{y}=s\,\left(\theta \right)\,d\,y_{\lambda }\,\left(x,y\right) & \end{array}$$

We name this class of model ERICA. For simplicity, and to keep the system analytical, we restricted ourselves first to s functions linear by piece, that is, $s=s_{*}>1$ for one sector and s=1 otherwise. The sped up sector is centered at angle α and has width β, so that the modified period of the cycle is $T_{s_{*}}=\beta /s_{*}+\left(2\,\pi -\beta \right)$. It is also convenient to define the rescaled angular velocity $\omega_{s_{*}}=2\,\pi /T_{s_{*}}=\frac{2\,\pi \,s_{*}}{\beta +\left(2\,\pi -\beta \right)\,s_{*}}$. From there, the phase of the cycle as a function of the angle θ in the plane is the simple linear transformation (defining $\theta_{0}=\alpha -\beta /2$):

(14) $$\phi_{s}\,\left(\theta \right)=\omega_{s_{*}}\,\theta =\theta \,\frac{2\,\pi \,s_{*}}{\beta +\left(2\,\pi -\beta \right)\,s_{*}}$$

for $0<\theta <\theta_{0}$,

(15) $$\phi_{s}\,\left(\theta \right)=\omega_{s_{*}}\,\left(\theta_{0}+\frac{\theta -\theta_{0}}{s_{*}}\right)=\left(\theta_{0}+\frac{\theta -\theta_{0}}{s_{*}}\right)\,\frac{2\,\pi \,s_{*}}{\beta +\left(2\,\pi -\beta \right)\,s_{*}}$$

for $\theta_{0}<\theta <\theta_{0}+\beta$, and

(16) $$\phi_{s}\,\left(\theta \right)=\omega_{s_{*}}\,\left(\theta_{0}+\frac{\beta }{s_{*}}+\theta -\left(\theta_{0}+\beta \right)\right)=\left(\theta_{0}+\frac{\beta }{s_{*}}+\theta -\left(\theta_{0}+\beta \right)\right)\,\frac{2\,\pi \,s_{*}}{\beta +\left(2\,\pi -\beta \right)\,s_{*}}$$

for $\theta_{0}+\beta <\theta <2\pi$. Those functions ensure that the rate of phase evolution in sector $\theta_{0}<\theta <\theta_{0}+\beta$ is $1/s_{*}$ times the rate in the other sectors (compare θ coefficients in Equations 14-16), that angle 0 in θ is phase $\phi_{s}=0$, and that phase is continuous so that $\phi_{s}\,\left(\theta_{0}\right)=\omega_{s_{*}}\,\theta_{0}$ and $\phi_{s}\,\left(\theta_{0}+\beta \right)=\left(\theta_{0}+\beta /s_{*}\right)\,\omega_{s_{*}}$. Notice also that for θ=2⁢π we get from Equation 16 $\phi_{s}\,\left(2\,\pi \right)=\left(\frac{\beta }{s_{*}}+2\,\pi -\beta \right)\,\frac{2\,\pi \,s_{*}}{\beta +\left(2\,\pi -\beta \right)\,s_{*}}=2\,\pi$ as expected after one full cycle.

A full study of the possible behaviors of the ERICA model will be published elsewhere. Appendix 1—figure 4 illustrates different shapes of PRC obtained by varying $\lambda ,s_{*}$, α, and β independently, for various choices of sped up sectors.

For optimization purpose, it is easier to numerically compute the PRC in the following way. We consider an ensemble of angles $\theta_{i}$ linearly spaced on the interval [0,2⁢π]. We then compute the corresponding position on the ERIC limit cycle, and compute numerically the angle $\theta_{i}^{\to \epsilon }$ of the point at distance ϵ on the right. By definition of ERICA, the PRC for each index i is then

(17) $$P\,R\,C^{m\,o\,d\,e\,l}\,\left(\phi_{s}\,\left(\theta_{i}\right)\right)=\phi_{s}\,\left(\theta_{i}^{\to \epsilon }\right)-\phi_{s}\,\left(\theta_{i}\right)$$

We checked that this PRC coincides both with the one computed from the integration of the ODEs (simulating the full stroboscopic map procedure) and with the analytical expression. To fit the experimental PRC, we run MC simulations to optimize parameters ϵ, λ, $s_{*}$, sped up sector location α and width β, and the location of the zero-phase reference point on the cycle $\phi_{0}$. We minimize $\chi^{2}$:

(18) $$\chi^{2}=\sum_{i}\frac{{\left(P\,R\,C_{i}^{c\,o\,r\,r}-P\,R\,C_{i}^{m\,o\,d\,e\,l}\right)}^{2}}{\sigma^{2}},$$

where $P\,R\,C_{i}^{c\,o\,r\,r}$ are data points of the corrected experimental PRC and σ is the noise, estimated as the root mean square error of a Fourier fit to data (see Figure 8A). We use Markov chains to generate distributions of parameters and take the average values to plot the model limit cycle and PRC. The optimized parameter values are: ϵ=0.43, λ=0.53, $s_{*}=5.64$, α=1.51⁢π, β=1.15⁢π, $\phi_{0}=1.17\,\pi$.

#### Evidence for intrinsic period changes

The PRC shifts are interpreted as change of period of the intrinsic oscillator because of entrainment.

We first checked that such PRC shifts are really needed to explain data. To do this, we computed an average PRC by only taking into account entrainment periods between 130 and 150 min. If we assume there is no period change, we can compute the maximum entrainment period compatible with such PRC, and found it is equal to 170 min. However it is clear from our data that we can go beyond this entrainment period, confirming the shift of PRC, in agreement with a change of intrinsic period.

We further have two evidences for such changes from release experiments. In those experiments, we entrain the oscillators for three cycles at 170 min, then let it go, and measure both the phase and instantaneous periods of the oscillator (Figure 7—figure supplement 1). When we measure the period, we first find it takes several cycles to come back to the intrinsic period, showing a long lived effect, unlikely coming from some artifact of period computation (Figure 7—figure supplement 1). A more direct way to estimate period change is to compute the return maps after the last pulse of DAPT. In Figure 8—figure supplement 3A, we consider all release experiments, which were done with *zeitgeber* period of 170 min. We take the phase one full cycle after the last pulse, and compute the return map from this phase (so for the first full cycle with no pulses). From this, we can compute the length of the cycle to have an average phase difference of 0 (corresponding to the period two full cycles after the last pulse). We found a cycle length of 150 min, compatible with the instantaneous period measurement and incompatible with a fast return to a 140 min intrinsic period.

We further show the Arnold tongue computed numerically with a similar PRC (1:1 entrainment region) and corresponding isophases assuming a fixed intrinsic period of 140 min in Figure 8—figure supplement 3B. We see much narrower width clearly incompatible with the data, again confirming the need for an intrinsic period change.

The last evidence is both more indirect and more mathematically grounded. It comes from the sigmoidal shape of the experimental entrainment phase as a function of *zeitgeber* period (Figure 7B, Figure 8E). In a nutshell, flatness of this curve is incompatible with the classical PRC theory, but can be easily explained if the internal period $T_{o\,s\,c}$ changes linearly with $T_{z\,e\,i\,t}$.

To see this, we start with the classical condition for stability of the entrainment phase (see, e.g., Izhikevich, 2006),

(19) $$-1<{\frac{dPTC}{d\phi }|}_{\phi =\phi_{ent}}<1$$

or equivalently:

(20) $$-2<{\frac{dPRC}{d\phi }|}_{\phi =\phi_{ent}}<0$$

Now we also have by definition of the entrainment phase $\phi_{e\,n\,t}$:

(21) $$P\,R\,C\,\left(\phi_{e\,n\,t}\right)=\frac{2\,\pi }{T_{o\,s\,c}}\,\left(T_{o\,s\,c}-T_{z\,e\,i\,t}\right),$$

where the factor $\frac{2\,\pi }{T_{o\,s\,c}}$ is the unit conversion from time to phase. This equation defines in an implicit way a function $\phi_{e\,n\,t}\,\left(T_{z\,e\,i\,t}\right)$, and the corresponding experimental curve is plotted in Figure 8E for our data. Taking the derivative on both sides with respect to the *zeitgeber* period, we get:

(22) $${\frac{d\,P\,R\,C}{d\,\phi }|}_{\phi =\phi_{e\,n\,t}}\,\frac{d\,\phi_{e\,n\,t}}{d\,T_{z\,e\,i\,t}}=-\frac{2\,\pi }{T_{o\,s\,c}}$$

This allows to implicitly define the curve $\phi_{e\,n\,t}\,\left(T_{z\,e\,i\,t}\right)$ with the help of the P⁢R⁢C:

(23) $$\frac{d\,\phi_{e\,n\,t}}{d\,T_{z\,e\,i\,t}}=-\frac{2\,\pi }{T_{o\,s\,c}}\,{\left({\frac{d\,P\,R\,C}{d\,\phi }|}_{\phi =\phi_{e\,n\,t}}\right)}^{-1}$$

There is a geometric interpretation here: the curve $\phi_{e\,n\,t}\,\left(T_{z\,e\,i\,t}\right)$ is in fact a (rescaled) +π/2 rotation of the PRC! This can be seen here because Equation 23 combines a mirror image of the PRC along the y axis (minus sign) with a mirror image along the first diagonal (power −1 which corresponds to the inversion function exchanging ϕ and P⁢R⁢C(ϕ,P⁢R⁢C⁢(ϕ))→(P⁢R⁢C⁢(ϕ),ϕ)). We know from classical group theory that two mirror images give one rotation with an angle equal to twice the angle between the axis (so here 2⁢π/4=π/2). For instance, if the PRC is sinusoidal, $\phi_{e\,n\,t}\,\left(T_{z\,e\,i\,t}\right)$ looks itself like (half a period) a vertical sinusoidal, as can be clearly seen, for example, in Granada et al., 2009. In Figure 8—figure supplement 3C, we further illustrate this rotation using the PRC computed from the data: the PRC rotated by π/2 is compared with the curve $\phi_{e\,n\,t}\,\left(T_{z\,e\,i\,t}\right)$ computed numerically from it, showing perfect agreement.

Now, since the geometry of the PRC is constrained by Equation 20, this in turn imposes geometric constraints on $\phi_{e\,n\,t}\,\left(T_{z\,e\,i\,t}\right)$ , for example, combining Equations 20-23 and counting time in units of the intrinsic period $T_{o\,s\,c}$, $2\,\pi /T_{o\,s\,c}=1,$ we get

(24) $$\frac{d\phi_{ent}}{dT_{zeit}}>\frac{1}{2}$$

This defines an absolute, *minimum* slope of the curve $\phi_{e\,n\,t}\,\left(T_{z\,e\,i\,t}\right)$. However, we see experimentally in Figure 8E that the entrainment phase is becoming almost constant for low and high values of the entrainment period, indicating a zero slope, which is thus theoretically impossible from Equation 24.

Now the simplest way to reconcile this observation with this calculation is to assume that the intrinsic period $T_{o\,s\,c}$ in fact depends on the *zeitgeber*. We then get a generalized version of Equation 22 with changing intrinsic period

(25) $${\frac{d\,P\,R\,C}{d\,\phi }|}_{\phi =\phi_{e\,n\,t}}\,\frac{d\,\phi_{e\,n\,t}}{d\,T_{z\,e\,i\,t}}=\frac{2\,\pi }{T_{o\,s\,c}}\,\left(\frac{d\,T_{o\,s\,c}}{d\,T_{z\,e\,i\,t}}\,\frac{T_{z\,e\,i\,t}}{T_{o\,s\,c}}-1\right)$$

giving the new implicit definition

(26) $$\frac{d\,\phi_{e\,n\,t}}{d\,T_{z\,e\,i\,t}}=\frac{2\,\pi }{T_{o\,s\,c}}\,\left(\frac{d\,T_{o\,s\,c}}{d\,T_{z\,e\,i\,t}}\,\frac{T_{z\,e\,i\,t}}{T_{o\,s\,c}}-1\right)\,{\left({\frac{d\,P\,R\,C}{d\,\phi }|}_{\phi =\phi_{e\,n\,t}}\right)}^{-1}$$

Now we see that when $\frac{d\,T_{o\,s\,c}}{d\,T_{z\,e\,i\,t}}\,\frac{T_{z\,e\,i\,t}}{T_{o\,s\,c}}∼1$, the right-hand side of Equation 26 can be very small, so that one can get $\frac{d\,\phi_{e\,n\,t}}{d\,T_{z\,e\,i\,t}}∼0$, meaning that the entrainment phase barely changes as observed experimentally in Figure 8E. This effect will also come with a considerable enlargement of the Arnold tongue as discussed below. Also we notice that $\frac{d\,T_{o\,s\,c}}{d\,T_{z\,e\,i\,t}}\,\frac{T_{z\,e\,i\,t}}{T_{o\,s\,c}}=\frac{d\,\ln T_{o\,s\,c}}{d\,\ln T_{z\,e\,i\,t}}∼1$ could be indicative of a mechanism where the oscillator adapts its intrinsic period (here to the *zeitgeber* period).

Practically, we obtain the $T_{o\,s\,c}\,\left(T_{z\,e\,i\,t}\right)$ dependency by fitting the stroboscopic maps and entrainment phase from all experiments with 2.0 µM DAPT, using the PTC of our optimized model (Figure 8—figure supplement 3D-E). The values of $T_{o\,s\,c}$ are shown as data points in Figure 8D and Figure 8—figure supplement 3F.

#### Arnold tongue computations

To build the Arnold tongues, we first need to interpolate the period changes for period values not used in entrainment experiments. We used cubic spline interpolation to draw Figure 8D. For periods outside of the range of entrainment, in the absence of data some arbitrary choices have to be made. We know experimentally that entrainment does not occur below ∼120 min and above ∼200 min which indicates that $\frac{d\,T_{o\,s\,c}}{d\,T_{z\,e\,i\,t}}$ is becoming smaller around those *zeitgeber* periods. More biologically, this indicates that the system does not adjust to any entrainment period. Again some arbitrary choices have to be made, but based on those constraints and our own interpolation, a cut-off for $T_{o\,s\,c}$ of ±20% of the natural period of 140 min was assumed, and is further consistent with the relative period changes observed experimentally in zebrafish segmentation clock mutants (Herrgen et al., 2010) and theoretically derived from delayed coupled models (Moreno-Risueno et al., 2010). With such cut-off, the obtained entrainment range of *zeitgeber* periods is from 118 to 200 min.

Computation of Arnold tongue and isophases in Figure 8F was done by iterations of the return map Equation 2 and detection of fixed points, using as the intrinsic period the interpolation and extrapolation shown in Figure 8—figure supplement 3F.

Lastly, we can use this model to compute the entrainment phase for different DAPT concentrations. For the main concentration of 2.0 µM DAPT, we have the optimized amplitude of perturbation ϵ=0.43. Assuming that the DAPT concentration is reflected in the strength of perturbation, we can find cross-sections of the isophases at different values of ϵ to have an excellent agreement with experiments, as illustrated in Figure 8—figure supplement 3G. We take ϵ=0.55 for 3.0 µM, ϵ=0.31 for 1.0 µM, and ϵ=0.13 for 0.5 µM DAPT, as shown on the Arnold tongue in Figure 8F. We capture both the presence/absence of entrainment and the $\phi_{e\,n\,t}\,\left(T_{z\,e\,i\,t}\right)$ relation. In particular, we see that for lower DAPT concentrations, the range of entrainment is smaller but we still get plateaus in entrainment phase consistent with the change of period we computed.
