# Supplementary material for: Arnold tongue entrainment reveals dynamical principles of the embryonic segmentation clock
Source: eLife. 2022 Oct 12;11:e79575. doi: 10.7554/eLife.79575 (PMC9560162; doi:10.7554/eLife.79575)
Supplement: Peer review history

# Editor report: Editor's evaluation

**DOI:** 10.7554/eLife.79575.sa0

**Authors:** Sandeep Krishna

**Affiliations:** https://ror.org/03gf8rp76 National Centre for Biological Sciences­‐Tata Institute of Fundamental Research India

This manuscript undertakes a fundamental study of the entrainment of the mouse segmentation clock to an external oscillating signal. This provides insight into both the nonlinear properties of the clock as well as how it might integrate global signals. By combining experimental measurement of the finite pulse phase response curve with theoretical analysis using tools from nonlinear dynamics, this work produces a compelling coarse-grained model of the segmentation clock, albeit in an in vitro setting. In future, it will be of great interest to see how the predictions of this coarse-grained view, in particular the consequences of operating near a bifurcation, hold up in an in vivo system. This work is likely to be of interest to developmental biologists, synthetic biology experts, theoreticians and others interested in the emergence of complex patterning in living systems.

# Decision letter

**DOI:** 10.7554/eLife.79575.sa1

**Affiliations:** https://ror.org/03gf8rp76 National Centre for Biological Sciences­‐Tata Institute of Fundamental Research India

In the interests of transparency, eLife publishes the most substantive revision requests and the accompanying author responses.

[Editors' note: this paper was reviewed by Review Commons.]

# Reply: Author response

**DOI:** 10.7554/eLife.79575.sa2

Reviewer #1 (Evidence, reproducibility and clarity (Required)):

Major comments:

Are the key conclusions convincing?

We discuss 4 key conclusions.

1. A PRC of the segmentation clock was constructed.

Although the authors have produced an interesting phase map, the regulation function F(\phi) of the circle map does not give the phase response curve (PRC) (Hoppensteadt & Keener 1982, Guevara & Glass 1982). This holds only when the system is stimulated with very short pulses (ideally Dirac δ), but the experimental pulses here are a quarter of the intrinsic period.

There are several definitions of the PRC (Dirac pulses PRCs, linear PRCs, etc.). We use the general definition from Izhikievich, 2007: “In contrast to the common folklore, the function PRC (θ) can be measured for an arbitrary stimulus, not necessarily weak or brief. The only caveat is that to measure the new phase of oscillation perturbed by a stimulus, we must wait long enough for transients to subside“.

The corresponding equation from Izhikievich (section 10.1.3) is

$PRC(\theta )=\;\theta_{\text{new}}-\theta$

which is equivalent to our Equation 1.

Hence, the key assumption we make is that after perturbing the system, we are back on the limit cycle as pointed out by Izhikievich. We think this is a reasonable assumption, because the perturbation we impose is relatively weak, despite pulsing for almost one quarter of the intrinsic period. The concentrations of DAPT we used in this current study are just enough to elicit a measurable response, and further lowering the concentration does not result in entrainment within our experiment time (0.5uM, Figure S7B in submitted version of the manuscript). Additionally, we previously reported that periodic pulsing with 2uM DAPT did not result in change of the Notch signaling activity with respect to control samples (Sonnen et al., 2018). Along similar lines, the DAPT drug concentrations we used are much lower compared to what has been used in previous studies aiming to perturb signaling levels, e.g. 100uM and 50uM used in study of segmentation clock in zebrafish embryos (Özbudak and Lewis, 2008 and Liao et al., 2016, respectively), and 25uM used in study of the segmentation clock in mouse PSM cells (Hubaud et al., 2017). Combined, we reason that we apply weak perturbations that allow to extract the PRC of the segmentation clock during entrainment. Additional evidence that indeed we have revealed a meaningful PRC is provided below, please see our response to point #3.

2. Furthermore, in eq. 1 T_ext must be the winding number, and the modulus must be in units of phase, either one or two pi, for the circle map to be correct. Thus, calling the measured response of the system a PRC is not convincing.

We thank the reviewer for pointing this out. We indeed rescaled everything to express the PRC in units of phase. We made this more explicit and updated equations throughout the text.

3. The system is being entrained. Technically, It would also be easier to get the stroboscopic maps in the quasi-periodic regime since all the points in the circle will be sampled. Since no quasi-periodic response was demonstrated, the claim of entrainment is not convincing.

While, in principle, PRC can be indeed obtained from responses in the “quasi-periodic” regime, such an approach is, in practice, challenging due to the intrinsic noise. The closest approximation to this is the phase response after the first pulse, that we reproduce in Author response image 1 and compare to our inferred PRC, where we indeed clearly see a high noise level. Nevertheless, also the PRC based on the first pulse is in agreement with the PRC we derived from the entrainment data.

**Author response image 1.** 

In the entrained regime, one can get a much more reliable estimate of the phase response despite the noise. The level of noise in the stroboscopic map lowers as the samples approach entrainment (Figure S12), and the entrainment phase itself is a reliable statistical quantity that can be used to infer regions of the PRC as the detuning is varied.In addition, and maybe even more importantly, we identify several key features characteristics of entrainment, such as the change of entrainment phase as a function of detuning (Figure 7, Figure S6-S7 in submitted version of the manuscript) and the dependency of the time to entrainment as a function of initial phase (Figure 6). While additional features can be linked, in theory, with entrainment, i.e. period-doubling, higher harmonics (Figure 5), quasi-periodicity, we do not agree with the reviewer that all of these need, or in fact, can be found in the experimental data, in particular because of the influence of the noise. Conversely the positive experimental evidence that we provide for the presence of entrainment, combined with the theoretical framework we develop, justifies, in our view, the conclusions we make.

4**.** The response of the system to external pulses is compatible with a SNIC. This is compatible, but it is equally compatible with other explanations. Assuming that the PRC is the same as the regulation function F(\phi), the PRC in Kotani 2012 (PRL 2012 Figure 3C) would be a similar shape as that shown by the authors. Similar models to that in Kotani et al., have been studied, but a SNIC has not been found (an der Heiden & Mackey 1982). It is relatively straightforward to construct a phenomenological model with a SNIC, but having underlying biological insight is not guaranteed. No argument for choosing a SNIC is given, so this emphasis of the paper is not convincing.

It is true that the mapping of PRCs to oscillators is undetermined, in the sense that many systems could potentially give rise to similar PRCs. That said, there is value in parsimonious models, which often generalize very well despite their simplicity. This explains why in neuroscience, constant sign PRCs are generally associated with SNIC. There is a mathematical reason for this: 1-D oscillators with resetting (such as the quadratic fire-and-integrate model) are the simplest models displaying constant sign PRCs, and are the “normal” form for SNICs. In other words, SNIC bifurcations are among the simplest ones compatible with constant sign PRCs, and we think it is informative to point this out. In our manuscript, we go one step further by actually fitting the experimental PRC with a simple, analytical model that allows us to compute Arnold tongue for any values of the perturbation (contrary to more complex models).

Other models such as Kotani 2012 can display similar PRC shapes, but they are of mathematically higher complexity, and furthermore it is not clear how such systems might behave when entrained. For instance that model in particular uses delayed differential equations, and as such contains long term couplings, so that a perturbation might have effects over many cycles, which is not consistent with the hypothesis we here make of a relatively rapid return to the limit cycle. Furthermore, for more complex models, PRCs are analytical only in the linear regime, while our model is analytical for all perturbations. That said, we agree that other types of oscillators can be associated with constant sign PRCs, and we have given more details in this part, in particular we better emphasize the Class I vs Class II oscillators as a way to broaden our discussion on PRC, and emphasize the “infinite period” bifurcation category which is more intuitive and further includes saddle node homoclinic bifurcations.

5. The work demonstrates coarse graining of complex systems.

This conclusion is correct, but coarse graining theory-driven analysis and control of dynamical systems has been established for many years. What is new here is that it is applied specifically to the in vitro culture system of the mouse segmentation clock.

We agree it is new to successfully apply coarse-graining analysis and, importantly, control, to the in vitro culture system of the mouse segmentation clock. We also agree that such an approach has been pioneered and established for many years, especially in (theoretical) physics, but indeed, the key question is whether and how this can be applied to complex biological systems. Insights coming from theoretical considerations on idealized physical systems might not necessarily apply to biology, as already pointed out by Winfree.

There are still very few examples in biology with coarse graining similar to what we do here. We think there is immense value in demonstrating that quantitative insights, and control of the biological systems, can be obtained without precise knowledge of molecular details, which is still counter-intuitive to many biologists. In this sense, we think our report will be of interest to both colleagues within the field of the segmentation clock and also to anyone interested to in the question, how theory and physics guided approaches can enable novel insight into biological complexity.

Should the authors qualify some of their claims as preliminary or speculative, or remove them altogether?

Following on the points above, each of these needs to be corrected or re-done, and/or the conclusions need to be modified accordingly.

We have modified the manuscript in response to all those points.

6. Would additional experiments be essential to support the claims of the paper? Request additional experiments only where necessary for the paper as it is, and do not ask authors to open new lines of experimentation.

If the authors wish to make the strong claim of determining a true PRC, Dirac δ-like perturbation needs to be applied, or approximated by short time duration pulses compared to the intrinsic period.

Please refer to our response to point #1 and #3.

7. Are the suggested experiments realistic in terms of time and resources? It would help if you could add an estimated cost and time investment for substantial experiments.

It's not clear to this reviewer if it is feasible to deliver a very short pulse and record a response. But this may not be relevant, see above.

Please refer to our response to point #1 and #3.

Are the data and the methods presented in such a way that they can be reproduced?

Yes.

Are the experiments adequately replicated and statistical analysis adequate?

Yes.

Minor comments:

Specific experimental issues that are easily addressable.

No issues.

Are prior studies referenced appropriately?

Yes.

8. Are the text and figures clear and accurate?

Figure 1D illustrates how a PRC should be obtained, but doesn't show the experimental protocol applied in the paper.

Figure 1D is a general introduction on the phase description of oscillators and phase response. It demonstrates how a perturbation can change the phase and is not supposed to represent the experimental protocol. We describe how data are analyzed and how phases are extracted in Supplementary Note 1.

9. In Figure 5B, 10 μm DAPT, the traces are already synchronized before the pulse train starts, which makes the subsequent behavior difficult to interpret.

It appears here that by chance, the samples were already almost synchronized. We notice however that the establishment of a stable rhythm with the pulses (which here is not a multiple of the natural period) supports entrainment, and is already evident when looking at the timeseries with respect to the perturbation. The temporal evolution of the instantaneous period further confirms this, showing a change in period close to ½ *zeitgeber* period (which is very different from the natural period of ~140 mins). This also relates to point #35, in reply to both comments we have further expanded this figure to better show the 2:1 entrainment, adding statistics on the measured period and period evolution for a zeitgeber period of 300 mins.

10. Do you have suggestions that would help the authors improve the presentation of their data and Conclusions?

The text includes several paragraphs reviewing broad principles of coarse graining and making general conclusions. This is confusing, because, as mentioned above, there is no new general advance in this paper. The interesting contributions here are specific to the applications to the segmentation clock, and the text should be focused on this aspect.

As commented above for #3 , we respectfully disagree that there is no “new general advance” in this paper. It is far from obvious that a complex ensemble of coupled oscillators implicated in embryonic development would be amenable to such coarse-graining theory. Of note, we still do not have a full understanding of neither the core oscillators in individual cells, nor what slows these down and eventually stops the oscillations, and multiple recent works suggest that both phenomena are under transient nonlinear control (e.g. our own work in Lauschke 2013). It is remarkable that despite this lack of detailed mechanistic insight, general entrainment theory can be applied to the segmentation process at the tissue level. We further show that classical entrainment theory alone is not sufficient to account for the experimental findings. Specifically, we need to account for a period change that we interpret as an internal feedback, an insight that would be impossible without our coarse-graining approach. While the results might of course be specific to the segmentation process, we think our approach motivated by coarse-graining theory and leading to new insights into the process is of general interest. We tried to make these points explicit in our conclusion.

Reviewer #1 (Significance (Required)):

Describe the nature and significance of the advance (e.g. conceptual, technical, clinical) for the field.

Description of the complex mouse segmentation clock in terms of a simple model and its PRC is an interesting, original and non-trivial result. The proposal that the segmentation clock is close to a SNIC bifurcation provides a consistent dynamical explanation of slowing behavior that has been recognized for some time, but not fully understood. This proposal also raises a hypothesis about the behavior of the underlying molecular regulatory networks, which may be tested in the future. The increase or decrease of the intrinsic period due to the zeitgeber period is not expected from theory, pointing to structures in internal biochemical feedback loops, an idea which again may be tested in the future. Also surprising from a theoretical perspective, the spatial gradient of period in the system persisted after entrainment. Although the categorization of the generic behavior is interesting, by its nature there is little from this that might give a typical developmental biologist any conclusions about pathways or molecules. The successes and limits of the theoretical description do nevertheless focus future attention on interesting behaviors.

11**.** Place the work in the context of the existing literature (provide references, where appropriate).

Such an analysis of the segmentation clock is based strongly on the experimental system and results in Sonnen et al., 2018, and goes well beyond it in terms of the dynamical analysis. It provisionally categorizes the mouse segmentation clock as a Class I excitable system, allowing its dynamics at a coarse grained level to be compared to other oscillatory systems. In this aspect of simplification, it is similar to approach of Riedel-Kruse et al., 2007 who used a mean-field model of oscillator coupling to explain the synchrony dynamics observed in the zebrafish segmentation clock in response to blockade of coupling pathways, thereby allowing a high-level comparison to other synchronizing systems.

It is interesting the reviewer sees similarities with the work of Riedel-Kruse et al., which uses a mean-field variable Z that corresponds to a classical approach, as described in Pikovsky’s textbook, to quantify synchronization of oscillators. In our view, while of course we work in the same context of coupled oscillators in the PSM, our approach based on perturbing and monitoring the system’s PRC in real-time provides a novel strategy to gain insight. This is evidenced by the fact that our quantifications of synchronization and insight into the PRC is the basis to exert precise control of the pace and rhythm of segmentation.

State what audience might be interested in and influenced by the reported findings.

Developmental biologists, biophysicists.

Define your field of expertise with a few keywords to help the authors contextualize your point of view. Indicate if there are any parts of the paper that you do not have sufficient expertise to evaluate.

Developmental biology, somitogenesis, dynamical systems theory, biophysics, cell signaling.

Reviewer #2 (Evidence, reproducibility and clarity (Required)):

Summary:

This is a beautifully elegant study that tests how previously published theoretical predictions about entraining nonlinear oscillators applies to a biological oscillator, the segmentation clock. The authors use a combination of state of the art experimental techniques, signal processing and analytical theory to reach a series of interesting and novel conclusions.

They show that the segmentation clock period can be entrained through Notch inhibitor (DAPT) pulses acting as an external clock (referred to as zeitgeber) using a previously developed and sophisticated microfluidic perfusion system. Pulsing DAPT every 120 to 180min can change the internal clock period while entrainment beyond this range leads to higher order coupling to the zeitgeber period, i.e. entrainment of every other pulse. They then perform entrainment experiments where the concentration of DAPT is changed to elicit a change in the strength of interaction between the internal clock and the external stimulus (referred to as zeitgeber strength); interestingly at low strength response to entrainment is more variable leading to entrainment occurring in some samples while others remain unaffected (Figure 4A); overall, higher concentration leads to faster entrainment (Figure 4C). The experimental data is then analysed using stroboscopic maps to reveal that a stable entrainment phase shift is achieved between the internal clock and the external zeitgeber. Phase response curve (PRC) analysis indicates that the system response is not sinusoidal but predominantly characterised by negative PRC, a behaviour consistent with saddle-node on invariant cycle (SNIC); it also reveals that the intrinsic period changes in a non-linear way and that this effect is reversible when external stimulation stops. Finally, a theoretical model is proposed to represent the segmentation clock as a dynamical system; this is based upon Radial Isochron Cycle with Acceleration (ERICA), an extension motivated by the PRC analysis results which are incompatible with a Radial Isochron Cycle (RIC); this model has predictive capability and could be used to design new control strategies for entrainment of the segmentation clock.

This study makes a series of key conclusions which are of particular importance in understanding the dynamic response of a biological oscillators. Firstly, given it's the characteristics of the dynamic response to entrainment, the segmentation clock is likely close to a SNIC bifurcation and this can explain the tendency for relaxation of the period over time. Secondly, the clock period was changed in a non-linear way in the direction of the zeitgeber period, a finding which is interpreted to indicate the presence of feedback of the segmentation clock onto itself, potentially via Wnt. This makes an excellent prediction that if tested experimentally would greatly improve the impact of the study. It is also noted that the entrainment of the segmentation clock does not abolish spatial periodicity and phase wave emergence suggesting that single cell oscillators can adjust to periodic perturbation while maintaining emergent properties. This is also a significant result that would need to be followed up with experiments and computation however would be best suited to a separate study.

Major comments:

12. The coarse graining is a major point that would need to be clarified since the rest of the analysis and theoretical modelling in the paper flow from this. Firstly, the interpretation of the schematic in Figure 1A on experimental data collection is not immediately obvious to the reader, lacks a clear flow between the different panels or steps (which could be numbered for example) and does not have a legend to indicate the different colour mapping.

We are grateful to the reviewer for this comment. We have implemented in Figure 1A all the changes suggested by the reviewer: we numbered the different steps and have added a colour mapping. In addition we have rephrased the caption of Figure 1A to better connect the experimental steps.

13. Secondly, Figure 2A which explicitly addresses coarse graining is not clear enough. Is the message here that by excluding the inner parts of the sample with a radial ROI, a similar dynamic response is observed over time?

Yes, indeed this is the point and we have adjusted the figure and text to explain this better. Our goal is to focus on the quantification of segmentation pace and rhythm. This is best captured by reporters such as LuVeLu, which has maximum intensity in regions where segment forms, and which dynamics is known to be strongly correlated to segmentation (Aulehla et al., 2007; Lauschke and Tsiairis et al., 20132). The global ROI is thus expected to precisely capture these segmentation and clock dynamics and we have now included more validation data and have also edited the text to make this very important point clearer:

“To perform a systematic analysis of entrainment dynamics, we first introduced a single oscillator description of the segmentation clock. We used the segmentation clock reporter LuVeLu, which shows highest signal levels in regions where segments form Aulehla_A_2007}. Hence, we reasoned that a global ROI quantification, averaging LuVeLu intensities over the entire sample, should faithfully report on the segmentation rate and rhythm, essentially quantifying 'wave arrival' and segment formation in the periphery of the sample.”

Figure 2A indeed shows that the dynamics (from the timeseries) is very similar when considering the entire field of view (global ROI) or when considering only the periphery of the 2D-assay (excluding central regions). We modified Figure 2A to clarify this point by indicating each measurement as either global ROI or global ROI minus the diameter of the excluded circular region (e.g. global ROI – 50px). We also emphasized in the caption that timeseries are obtained using global ROI, unless otherwise specified. We included a link (https://youtu.be/fRHsHYU_H2Q) in the caption to a movie of 2D-assay subjected to periodic pulses of DAPT (or DMSO) and corresponding timeseries from global ROI.

Since the inner part of the sample corresponds to the posterior side how do we interpret similarities and differences between signals with different ROIs?

As stated above, the global ROI measurements essentially capture the signal at the periphery where segments form and faithfully mirrors segmentation rate and rhythm. We have now included a comparison to the center ROI, also in response to reviewer’s comments, see our response #34.

The result shows that the period and PRC in the center matches the one found in the periphery, i.e. global ROI. We have shown previously that center and periphery differ in their oscillation phase by 2pi, i.e., one full cycle (Lauschke et al., 2013). We interpret these findings as confirmation of our analysis strategy, i.e. the global ROI allows a very reproducible, unbiased quantification that reports on segmentation clock and period.

14. A quantitative analysis of essential coarse-grained properties such as period and amplitude should be performed for different ROIs and across multiple samples. As this effectively masks any spatial differences, limitations of this approach should be clearly stated in the Discussion. For example in lines 466-470 where it is difficult to interpret the slowing down tendency and relate back to single cell level.

As outlined in our response to comment #13 and also #34, we chose an analysis that allows to determine the segmentation pace and rhythm, i.e. segment formation, which is well captured by LuVeLu signal and a global ROI analysis. We agree that a spatially resolved analysis of dynamic behaviour is important (and indeed a gradient of amplitude might be relevant in such context), but we think this is beyond the scope of the current study focused on the system level segmentation clock behaviour. We have revised the discussion as suggested by the reviewer to make this point approach and the need for future studies clearer.

15. The functional characterisation of the sample using LFNG, AXIN2 and MESP2 is unclear. The images included in Figure 2D representing expression observed when tissue explants are grown within the microfluidic chip are difficult to interpret and would require a more detailed description of anterior-posterior, pillars etc; it is also difficult to view the bright-field since it is presented as a merged image.

It is particularly difficult to see the somite boundaries for the same reason. In lines 113-117 the authors state that the global oscillation period matches the periodic boundary formation. How do we reach this conclusion from these images? What is the variability between samples?

If these two issues would be addressed it would increase confidence in the coarse graining argument and thus would strengthen the importance of the findings in the study.

We thank the reviewer for this feedback, and we have added more quantifications to address this point directly in the modified Figure 2. Importantly, we added the quantification of the rate of segmentation in multiple samples based on segment boundary formation (new Figure 2D) and compared this to the global ROI quantifications using the reporter lines LuVeLu. This data provides clear evidence that the quantification of global ROI reporter intensities closely matches the rate of morphological segment boundary formation. In addition, we show that segment formation and also Wnt-signaling oscillations (Axin2-Achilles) and the segmentation marker Mesp2 (Mesp2-GFP) are all entrained to the *zeitgeber* period. We have also revised the text to clarify this important validation of our quantitative approach.

In addition, we provide, in the revised Figure Suppl. 2, details of entrained samples, focusing on the segmenting regions. The brightfield and reporter channels were separated, emphasizing the segment boundaries and the expression pattern of the reporters. For ease of visualization, these samples were also re-oriented so that the tissue periphery (corresponding to anterior PSM) is at the top while the tissue center (corresponding to the posterior PSM) is at the bottom. This now additionally better shows the localization of the different reporters with respect to the segment boundary. We also included supplementary movies showing timelapse of samples expressing either Axin2-GSAGS-Achilles or Mesp2-GFP that were subjected to periodic DAPT pulses, with their respective controls.

Several minor points could be addressed to improve the manuscript and are listed below:

16. Figure 1 A the colormap and axes for the oscillatory traces should be defined

We thank the reviewer, and we have modified the figure accordingly (related to point 12). A colormap and axes for the illustrated timeseries are now included.

17. Strength of zeitgeber is not defined and there is no analytical expression provided; how does it relate to DAPT concentration? Is the fact that low DAPT concentration corresponds to weak strength expected or is it a result?

Zeitgeber strength generally refers to the magnitude of the perturbation periodically applied to an oscillator. With DAPT pulses, our expectation was that both the duration of the pulse and the drug concentration could influence the strength. Practically, the pulse duration was kept constant for all experiments and the concentration was varied. We thus expected that DAPT concentration would indeed be correlated to zeitgeber strength. We have discussed multiple evidence supporting this assumption in the main text, and this is indeed a result. In particular, as explained in the section “The pace of segmentation clock can be locked to a wide range of entrainment periods”, higher DAPT concentration gives rise to faster and better entrainment, as expected from classical theory. In the context of Arnold tongue, weaker zeitgeber strength corresponds to narrower entrainment region, which is experimentally observed (Figure 8F, showing regions where the clock is entrained).

From a modelling standpoint, Zeitgeber strength corresponds to parameter A which is the amplitude of the perturbation. Possible zeitgeber strength was inferred from the model by matching the experimental entrainment phase $\phi_{ent\;}(T_{zeit})$ with that obtained from the model isophases. As explained in Supplementary Note 2, we tested four concentrations of DAPT (0.5, 1, 2, and 3 uM) respectively corresponding to A values of 0.13, 0.31,0.43, 0.55. As we can see, those A values are not linear in DAPT concentrations, which is expected since multiple effects (such as saturation) can occur.

18. In some figures it looks like the amplitude of oscillations may change with DAPT concentration and hence zeitgeber strength? Is this expected?

We have not systematically analyzed the amplitude effect and have, intentionally, focused on the period and phase readout as most robust and faithful parameters to be quantified. Regarding the amplitude of LuVeLu reporter, we are cautious given that it is influenced, potentially, by the (artificial) degradation system that we included in LuVeLu, i.e. a PEST domain. This effect concerns the amplitude, but not the phase and period, explaining our strategy.

That said, we agree with the referee that DAPT concentrations might change the amplitude of oscillations. Such change could even play a role in the change of intrinsic period (in fact a similar mechanism drives overdrive suppression for cardiac oscillators, Kunysz et al., 1995). But since the change of period can be more easily measured and inferred, we prefer to directly model it instead of introducing a new hypothesis on amplitude/period coupling, at least for this first study of entrainment.

19. Figure 2A including the black area creates confusion and it is unclear which ROI is used in the rest of the study; consider moving this to a supplementary figure perhaps

We thank the reviewer for this feedback (related to point #13), and we have modified the figure accordingly. As we responded to point 13: We modified Figure 2A, by indicating each measurement as either global ROI or global ROI minus the diameter of the excluded circular region (e.g. global ROI – 50px). We also emphasized in the caption that timeseries are obtained using global ROI, unless otherwise specified.

20. What type of detrending is used in Figure 2 and throughout (include info in the figure legend)?

We used sinc-filter detrending, described and validated in detail previously (Mönke et al., 2020), as specified in Supplementary Note 1: Materials and methods > H. Data analysis > Monitoring period-locking and phase-locking: In this workflow, timeseries was first detrended using a sinc filter and then subjected to continuous wavelet transform. We thank the reviewer for pointing out that this detail is lacking in the figure captions, and we have modified the captions accordingly.

21. Figure 2D merged images are difficult to read/interpret (see major comments)

We thank the reviewer for this comment, and we have modified the figure accordingly (please see response to related point #15).

22. Kuramoto order parameter is used to quantify the level of synchrony across the different samples however it is not defined in the text. Is it also possible to assess variability in each sample? For example how quickly does entrained occur in each sample? How faithfully the peaks of expression beyond 80min (to exclude initial unsynchronised state) match with zeitgeber time? This would help make the point that weak strength leads to a more variable response which is an interesting finding.

We have now added a mathematical definition of the Kuramoto parameter in Supplementary Note 1.

A high order parameter corresponds to coherence between samples, as also elaborated in respective figure captions (e.g. in the caption for polar plots in Figure 4D)*.*

In terms of variability in response to entrainment, we thank the reviewer for the comments, which has prompted us to perform an additional analysis, now included as Figure S13 in the Supplement.

Briefly, Author response images 2–6 and Figure 4—figure supplement 2 show how different samples get synchronized with the zeitgeber. To do this, we first represent the zeitgeber signal as a continuous uniformly increasing phase (“zeitgeber time”) with period $T_{zeit}:$${\dot{\phi }}_{zeit}=2\pi /T_{zeit}.$ The initial condition for $\phi_{zeit}$ is chosen so that the zeitgeber phase at the moment of last pulse is matching the experimental entrainment phase for each $T_{zeit}$. We plot cos ϕ(t) for each sample (dotted lines) and the zeitgeber phase (magenta line). To quantify how well each sample is following the zeitgeber time, we compute the Kuramoto parameter: $R_{j}(t)\;=\;|\frac{1}{2}(e^{i\phi_{j}(t)}+e^{i\phi_{zeit}(t)})\;|$. By the end of experiment most samples reach $R_{j}\;\approx 1$, indicating entrainment. Most samples need ∼3 zeitgeber cycles to become entrained. For $T_{zeit}=120$ min the entrainment takes much longer (edge of the Arnold tongue). For $T_{\text{zeit}}=140$ min there is much variability, which can be explained by the horizontal region in the PRC around the entrainment phase. As suggested by the referee, synchronization is faster for higher DAPT concentration. So those dynamics are indeed consistent with the expectation from classical PRC theory.

**Author response image 2.** T_zeit_ = 120.

**Author response image 3.** T_zeit_ = 140.

**Author response image 4.** T_zeit_ = 150.

**Author response image 5.** T_zeit_ = 160.

**Author response image 6.** T_zeit_ = 180.

23. Do samples change period to Tzeit in similar ways – i.e. patterns over time. It looks like the kuramoto order parameter and period drop initially – why?

We do not have a direct answer as to why the Kuramoto first order parameter and the period drop for the condition the reviewer specified. It has to be noted though that because of how wavelet analysis is done (cross-correlation of the timeseries with wavelets), the period and phase determination at the boundaries of the time series are less reliable (edge effects, see Mönke et al., 2020). Because of this, we should take caution when considering data to and from the first and last pulses, respectively. This was explicitly stated in the generation of stroboscopic maps: “As wavelets only partially overlap the signal at the edges of the timeseries, resulting in deviations from true phase values (Mönke et al., 2020), the first and last pulse pairs were not considered in the generation of stroboscopic maps.”

24. In Figure 4C why is the Kuramoto order parameter already higher in the 2uM DAPT conditions at the start of the experiment?

Samples can, by chance, start synchronously and this results in a high Kuramoto first order parameter. Because of this likelihood, it is thus important to interpret the entrainment behaviour of multiple samples using various readouts, in addition to a high Kuramoto first order parameter. We investigated entrainment of the samples based on several measures: multiple samples remaining (or becoming more) synchronous (because each sample actively synchronizes with the *zeitgeber*), period-locking (where the pace of the samples match the pace of the *zeitgeber,* which can be distinct from natural pace), and phase-locking (where there is an establishment of a stable phase relationship between the samples and the *zeitgeber*).

25. Figure 3C and Figure S2 require statistical testing between CTRL and DAPT in each condition.

p-values were calculated for the specified conditions and were added in the caption of the figures. These values are enumerated here:

– Figure 3C

170-min 2uM DAPT (vs DMSO control): p < 0.001

– Figure S2

120-min 2uM DAPT (vs DMSO control): p = 0.064

130-min 2uM DAPT (vs DMSO control): p = 0.003

140-min 2uM DAPT (vs DMSO control): p = 0.272

150-min 2uM DAPT (vs DMSO control): p = 0.001

160-min 2uM DAPT (vs DMSO control): p < 0.001

170-min 2uM DAPT (vs DMSO control): p < 0.001

180-min 2uM DAPT (vs DMSO control): p = 0.001

To calculate p-values, two-tailed test for absolute difference between medians was done via a randomization method (Goedhart, 2019). This confirms that the period of samples subjected to pulses of DAPT is not equal to the controls, except for the 140-min condition (where the *zeitgeber* period is equal to the natural period, i.e. 140 mins).

26. Figure 3A gray shaded area not clearly visible on the graph.

We have decided to remove the interquartile range (IQR) in the specified figure as it does not serve a crucial purpose in this case. By removing it in Figure 3A, the timeseries of individual samples are now clearer.

27. Figure 6C colour maping of time progression is not clearly visible on the graph; the interpretation of this observation is unclear in the text and the figure.

We agree that the low quality of the image is unfortunate, and it seems that our file was greatly compressed upon submission. We have checked the proper quality of figures in the resubmitted version of the manuscript.

Regarding the interpretation of Figure 6C, we conclude that in our experiments the entrainment phase is an attractor or stable fixed point, in line with theory (Granada and Herzel, 2009; Granada et al., 2009),. We had elaborated this in the text (lines 248-252 of the submitted version of the manuscript): “at the same zeitgeber strength and zeitgeber period, faster (or slower) convergence towards this fixed point (i.e. entrainment) was achieved when the initial phase of the endogenous oscillation (φinit) was closer or farther to φent.”

28. Figure 7A circular spread not clearly visible on the graph.

Similar to point #27, we have provided a high resolution graph for the re-submission and hopefully resolved this issue.

29. Figure S7A difficult to see the difference between colours

See point #28.

30. Is it possible to compare the PRC and the plots of period over time during entrainment? The PRC is mainly negative (Figure 8A1,A2), in my understanding this means a delay, however the periods seem to decrease over time before entraining to the Tzeit (Figure 3B). Is this reflective of a decrease in Kuramoto parameter and potential de-synchronisation of single cells before re-synchronisation at Tzeit?

To address this question, we now plot the Phase response with colors indicating pulse number in new Supplementary Figure S13. While capturing the entire PRC as a function of time would require many more experiments (in particular to sample the phases far from entrainment phase), we still clearly see that the PRCs appear to translate vertically as the oscillator is being entrained, i.e. the latter time points are shifted up (down) for T_zeit = 120 (170) min, respectively.

31. Figure 8A What is the importance/meaning of the PRC being similar shape between different entrainment periods? Does this reflect that the underlying gene network is the same?

If one single gene network is responsible for oscillations, we expect from dynamical systems theory that the PRC are not only of similar shape but actually the same, independent of the entrainment period. What is surprising is that the PRC for different entrainment periods do not overlap, and the simplest explanation for this is that the intrinsic period changes with entrainment, all things being kept equal (including the underlying gene networks). This relates to the previous point since we indeed observe that the PRC “translates” vertically with the pulse number for longer periods. The change of period might be due to a long-term regulation as detailed in the discussion.

32. The spatial period gradient and wave propagation under DAPT (Figure S8) should be included in the results and not just the discussion.

We fully agree with the reviewer that both the establishment and the maintenance of a spatial phase gradient is of great interest. However, many more experiments would be required to fully quantify and understand the processes at play here, which we believe to be out of the scope of the current manuscript. To keep the focus of the paper on the global segmentation clock itself, we prefer to keep this figure in Supplement.

Reviewer #2 (Significance (Required)):

We currently do not have a detailed understanding of how biological oscillators integrate local signals from their neighbours as well global external signals to give rise to complex patterning that is important for embryonic development. Main bottlenecks that hinder our understanding are lack of real-time endogenous dynamic response together with known global inputs as well as comprehensive models that can explain emergent behaviour in a variety of tissues.

This study goes a long way in addressing these bottlenecks in the embryonic tissue responsible for somite formation, a dynamical and oscillatory system also known as the segmentation clock. Firstly, they rely on a state-of-the-art previously developed system to entrain endogenous response in live tissue explants using precise microfluidic control. They test the complete range of exogenous perturbation periods and use an existing live reporter (LuVeLu) to monitor endogenous response. They also identify higher order coupling relationships whereby every other LuVeLu peak is entrained through external stimulation.

As the stimulation system does not control but rather perturb the endogenous response, the observations from LuVeLu provide a unique opportunity in understanding input-output relationships and thus describing the dynamic response of the segmentation clock. Authors propose to study dynamic behaviour of the clock using coarse-graining and focus on describing the overall response over time while amalgamating spatial information. Appropriate coarse-graining is an important strategy in addressing complex problems and is widely used. They use sophisticated methodology such as phase response curves and Arnold tongue mapping to make several important observations. For example the nonlinear shortening and elongation of the period in response to stimulation is particularly interesting since this may indicates a feedback of the clock onto itself potentially via Wnt. Another key observation is that the spatial periodicity and phase wave activity persists in the perturbed conditions suggesting that individual single cell oscillators can adjust their behaviour to external input while retaining coordination with their neighbours. Finally, the authors go on to construct a general dynamical model of the segmentation clock and use this to conclude that the intrinsic period of the oscillator is altered and that the oscillator can be considered excitable.

This work sheds light onto mechanisms of coordination of Notch activity in assemblies of cells observed in living tissue, an area of research that is important not only for somitogenesis but also for understanding gene expression patterning in many other tissues where Notch plays a critical role, for example in the development of the neural system and organs. As a study of a real-world nonlinear oscillator this work is directly of interest to theoreticians and synthetic biology experts interested in understanding complex patterning and emergence.

Reviewer #3 (Evidence, reproducibility and clarity (Required)):

In this manuscript, authors studied the system-level responses of the somite segmentation clock by the coarse-grained theoretical-experimental approach, applying the theory of entrainment to understanding the phase responses of mouse pre-somitic mesoderm (PSM) tissues in the presence of periodic perturbation of Notch inhibitor DAPT generated by micro-fluidics technique. It was demonstrated that the segmentation clock is responsive to diverse range of the perturbation-periods from 120 to 180 min, can be period- and phase-locked, and the efficiency is dependent of the DAPT concentration (input-strength). The authors also observed two cycles of the segmentation-clock ticking in single cycles of 300 or 350 min period-perturbation, suggesting that higher order (2:1 mode) entrainment. They also applied stroboscopic maps to analysis and found that entrainment-phases are dependent of period of DAPT pulses, which is recapitulating theoretical predictions. The estimation of the phase response curve (PRC) of the segmentation clock revealed that the inferred PRC is an asymmetrical and mainly negative function, which represents characteristic features in oscillators that emerge after saddle-node on invariant cycle (SNIC) bifurcation. These results also indicated that the segmentation clock changed the intrinsic period during entrainment.

Major comments:

33. I have major concerns about the relevance of the global time-series analysis proposed in Figure 2 and conclusion about the changes of the intrinsic period during entrainment. The validity of the global time-series analysis should be carefully analyzed, because it could bring artifacts in estimated values of the intrinsic period. The authors concluded (page 3, line 172) that the period calculated by the global analysis represents similar values with the rate of segment formation, but there is no data about the quantification of the periods of segmentation, such as the frequency of Mesp2 reporter expression.

We thank the reviewer for this feedback. We have now added the quantification of the period of segment formation (new Figure 2E) and show its strong correspondence to the dynamics of reporters used (Lfng, Axin2, and Mesp2). Please see also our response to point #15 with additional comments regarding the validation of the global time-series analysis.

34. Another related issue is the presence of spatial period gradient as mentioned (page 13, line 524). One possible approach to circumvent this issue would be "local" time-series analysis; for instance, just focusing on the "putative posterior" regions that are close to source-positions of waves. Authors can re-compute and estimate PRCs by using such a method.

We thank the reviewer for this suggestion and have accordingly now included the analysis of a localized ROI at the center (center ROI) of the 2D-assays (new Figures S5-S6). We also computed the PRC from center ROIs as shown in Author response image 7. We note strong correspondence between the global ROI and the center ROI.

**Author response image 7.** Comparison of the PRC obtained with global ROI and center ROI, T_zeit = 130 min (left) and 170 min (right).

35. I have another major concern about the evidence of higher order entrainment shown in Figure 5. If the 1:2 entrainment is successful, we can expect that the values of observed period is close to the half of the period of pulses; However, the period shown in Figure 5B looks like 185 min longer than the half of 350 min. Is this gap due to the temporal accuracy of time-lapse movies?

We do not think the discrepancy comes from a problem of temporal accuracy as the temporal accuracy is the same for all movies and there is no reason why there would be a specific issue for this set of experiments. In addition, we have re-analyzed the data to calculate the period from the stroboscopic maps. Mathematically speaking, we take the stroboscopic map as $\phi_{new\;}=\;(\phi_{old\;}+\frac{T_{zeit}}{T_{observed}}2\pi )\;mod\;2\pi \;,$ and use this to estimate the period of oscillation in entrained samples $T_{observed}$ , in particular inverting the formula for 1:2 entrainment we have :

$\frac{T_{zeit}}{T_{observed}}$=${(\phi }_{new\;}$-$\phi_{old\;})/2\pi$ + 2

The advantage of this method is that it gives a more ``instantaneous” estimation of the period.

The results are as follows:

350 10uM: 187 +- 8 min (average across entrained samples from the last zeitgeber period)

350 5uM: 193 +- 13 min (average across entrained samples from the last zeitgeber period)

300 2uM: 148 +- 8 min (averaged across entrained samples and from two last periods)

This additional analysis is in agreement with the wavelet analysis.

The reviewer is right that for 350 minutes, entrained samples show an observed period that is higher than expected, also based on this new additional analysis. The reason for this is not known. One explanation is the relatively short observation time, especially considering for pulses separated by as much as 350-minutes, i.e. only 3 pulses are applied. [We notice that for 300 minutes pulses, the period converges to 150 mins between the 3rd and the 4th pulse]. We have adjusted the text in the Results section to reflect that for 350min entrained samples, the observed period ‘approaches’ the predicted value, while for 300min entrained samples, the observed period is very close to it, i.e. 147mins In addition, we comment that the phase distribution narrows with time, another indication supporting higher order entrainment.

36. Also, authors showed the period evolution towards 1:2 locking with just one condition (350 min). Authors can show the data for multiple conditions as in Figure 3D, at least for 300 min and 325 min pulses and add the data about final entrained period with statistic analysis that supports the difference between the entrained period and the natural period (140 min).

We thank the reviewer for this feedback and have modified the figure accordingly. In particular, in Figure 5A, we have added the period evolution plot for samples subjected to 300-min periodic pulses of 2uM DAPT (or DMSO for control). Additionally, we have added Figure 5D, which plots the average period in the 300-min and 350-min conditions. We summarize the median average period here with computed p-values:

– 300-min pulses of 2uM DAPT (or DMSO for control): p-value = 0.191

CTRL: 130.39 mins

DAPT: 146.45 mins

– 350-min pulses of 5uM DAPT (or DMSO for control): p-value = 0.049

CTRL: 127 mins

DAPT: 174.86 mins

– 350-min pulses of 10uM DAPT (or DMSO for control): p-value = 0.016

CTRL: 142.82 mins

DAPT: 185.12 mins

Minor comments:

37. The authors can draw vertical lines indicating the T_zeit in Figure 3B, Figure 4B and Figure 5B in order to help comparisons between T_zeit and patterns of period (solid lines).

We thank the reviewer for this comment. We have accordingly added a horizontal line indicating Tzeit in Figures 3B, 4B, S4A, and S5A (figure panel numbers based on the submitted version of the manuscript). We similarly added a horizontal line indicating 0.5Tzeit in the period evolution plots of 300-min and 350-min conditions in Figures 5A and 5B, respectively.

38. In Figure 5A, the authors can show period evolution in the case of 300 min DAPT-pulses as shown in Figure 5B.

We thank the reviewer for this feedback (related to point #36), and we have modified the figure accordingly.

39. In Figure 6B DAPT panel, the authors can draw the points of phi_ent as shown in Figure 7A.

We thank the reviewer for this comment, and we have modified the figure accordingly.

40. In Figure 8F, authors can put the information about DAPT concentration at the right y-axis.

This is a similar comment as point #17, see above. In brief, we do not know the precise relation between the strength of the perturbation in our model and DAPT concentration, zeitgeber strength was inferred from the model by matching the experimental entrainment phase $\phi_{ent\;}(T_{zeit})$ with that obtained from the model isophases.

41. In Figure 8G, the PRC in the panel "170 mins" does not have any fixed point (cross sections with horizontal lines of "0" phase response). If entrainment is successful, there should be stable and unstable fixed points, but those are absent, although 170 min pulses succeeded in the entrainment as shown in Figure 3D. Authors can explain where the fixed points are.

The fixed points are indeed defined by the intersection with a horizontal line, but not with the ‘0’ line. They are found where the phase response compensates for the detuning/period mismatch, not at ‘0’ phase response. More precisely, starting with the stroboscopic map:$\phi_{\text{new\textbackslash{} }}=\;(\phi_{\text{old\textbackslash{} }}+PRC(\phi_{\text{old}})+\;\frac{T_{\text{zeit}}}{T_{\text{osc}}}2\pi )\;mod\;2\pi \;,$ a fixed point requires that $\phi_{new\;}=\;\phi_{old\;}\equiv \;\phi_{ent}$.

For 1:1 entrainment we then have:

$PRC(\phi_{ent})+\;\frac{T_{zeit}}{T_{osc}}2\pi \;=\;2\pi$,

$PRC(\phi_{ent})\;=\frac{2\pi }{T_{osc}}(T_{osc}-T_{zeit})$ - period mismatch with the zeitgeber, measured in units of phase.

Note however on Figure 8G that we further observe a vertical shift of the PRC, which prompted us to propose a change of the intrinsic period with $T_{zeit}$ (as explained in the text when we introduce Figures8A1-2).

Another way to visualize fixed points is offered in Figure 16 D-E, where we plot the inferred corrected PTC and the stroboscopic maps: there, fixed points correspond to intersections with the diagonal.

Reviewer #3 (Significance (Required)):

Although the phase-analysis has been widely applied to various biological systems, such as circadian clocks, cardiac tissues and neurons, this paper represents the first detailed experimental analysis of the segmentation clock based on the theory of phase dynamics. The major results are inline with theoretical predictions, whereas the suggestion about the SNIC bifurcation is attractive not only to the theoretical researchers but also to the experimental biologists; it has been believed that the segmentation clock consists of negative-feedback oscillator that emerge by Hopf bifurcation, whereas this paper proposes another possibility of the molecular network structure for the clockwork. This issue is related to recently proposed hypothesis about the excitable system in the segmentation clock based on the Yap signaling (Hubaud et al. Cell 171, 668 (2017)). However, unfortunately, discussion about detailed molecular networks are not abundant.

42. Thus, maybe the main readers are computational biologists and systems biologists.

We thank the reviewer for his/her significance comment. We have added comments on the bifurcation structure of the segmentation clock and on excitable systems in the discussion. While our focus is on coarse-graining so that we do not and cannot infer precise molecular details, we can still infer some properties of the underlying networks. In particular we now cite several papers explaining how systems with tunable periods/excitable are indicative of the interplay between positive and negative feedbacks. We think those considerations are of interest to a broad range of biologists interested in connecting experiments to theory.
